# Kynurenine pathway dysregulation in cognitive impairment and dementia: a systematic review and meta-analysis

**DOI:** 10.1007/s11357-025-01636-3

**Published:** 2025-05-08

**Authors:** Kyonghwan Choe, Lieke Bakker, Daniel L. A. van den Hove, Simone J. P. M. Eussen, Gunter Kenis, Inez H. G. B. Ramakers, Frans R. J. Verhey, Bart P. F. Rutten, Sebastian Köhler

**Affiliations:** 1 Department of Psychiatry and Neuropsychology, European Graduate School of Neuroscience (EURON), Faculty of Health, Medicine and Life Sciences (FHML), Mental Health and Neuroscience Research Institute (Mhens), Maastricht University, Maastricht, the Netherlands; 2 Alzheimer Center Limburg, Maastricht University, Maastricht, the Netherlands; 3 Department of Epidemiology, Maastricht University, Maastricht, the Netherlands; 4 School for Cardiovascular Diseases (CARIM) and Care and Public Health Research Institute (CAPHRI), Maastricht University, Maastricht, the Netherlands

**Keywords:** Kynurenine pathway, Kynurenines, Dementia, Alzheimer’s disease, Cognitive impairment, Cognitive functioning

## Abstract

The kynurenine pathway (KP) might be involved in pathophysiological processes associated with dementia, but clinical studies reported contradictory results. This systematic review and meta-analysis summarized the available evidence for (i) differences in KP metabolites in patients with cognitive impairment compared to cognitively healthy individuals and (ii) associations between KP metabolites and cognitive functioning. English, full-length articles with prospective, cross-sectional, or case–control study designs, published in Pubmed, Embase, PsychINFO, or the Cochrane Database of Systematic Reviews up to October 2023, were included. Random-effects meta-analyses of standardized mean differences (SMD) were performed. Heterogeneity, meta-regression, small study bias, and study quality assessments were carried out. Of 8797 retrieved studies, 98 were eligible for the systematic review. Meta-analyses comparing Alzheimer’s disease (AD) dementia patients to controls (n = 27 studies) indicated lower CSF levels of tryptophan (SMD = − 0.26 [95% CI − 0.41, − 0.12]), 3-hydroxykynurenine (− 0.21 [− 0.39, − 0.04]), anthranilic acid (− 0.28 [− 0.48, − 0.08]), and quinolinic acid (− 0.38 [− 0.56, − 0.21]) in AD dementia, while CSF levels of kynurenic acid were higher (0.18 [0.01, 0.35]). Blood levels of tryptophan (− 0.39 [− 0.51, − 0.28]), kynurenic acid (− 0.31 [− 0.47, − 0.15]), xanthurenic acid (− 0.34 [− 0.54, − 0.15]), and 3-hydroxyanthranilic acid (− 0.42 [− 0.61, − 0.22]) were lower in AD dementia. For some of these metabolites, similar directions were observed in meta-analyses comparing individuals with mild cognitive impairment with controls, although the number of included studies in these analyses was relatively small (n = 11). Associations with cognitive test scores were inconclusive and generally non-significant. These results suggest that AD dementia is associated with lower blood levels of several KP metabolites. Findings challenge current assumptions of neurotoxic quinolinic acid levels being associated with dementia.

**Supplementary Information:**

The online version contains supplementary material available at 10.1007/s11357-025-01636-3.

## Introduction

Dementia is a syndrome characterized by cognitive decline in multiple domains, resulting in dysfunction in activities of daily living, and has a considerable impact on patients, caregivers, and society overall [1,2]. According to the World Health Organization (WHO), around 55 million people worldwide were living with dementia in 2019, with 10 million new cases every year [3]. Alzheimer’s disease (AD) dementia is the most common type of dementia.

**Fig. 1:**
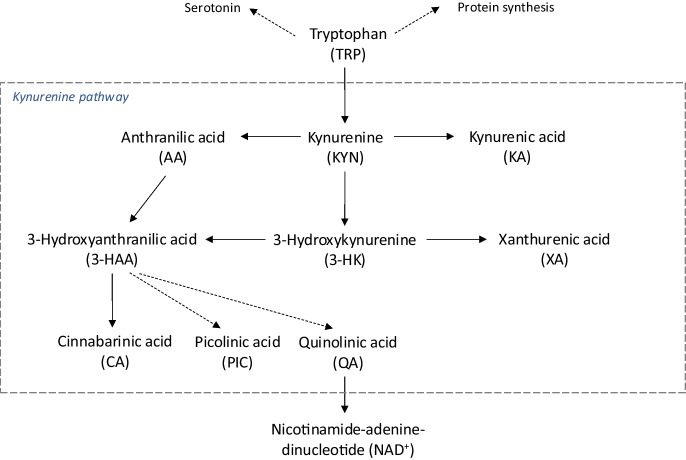
The kynurenine pathway. Kynurenine (KYN) is the central KP metabolite which can be further degraded into downstream metabolites, i.e., 3-hydroxykynurenine (3-HK), kynurenic acid (KA), and anthranilic acid (AA), by different enzymes. 3-HK can be further metabolized to xanthurenic acid (XA), or to 3-hydroxyanthranilic acid (3-HAA). 3-HAA, in turn, is synthesized to cinnabarinic acid (CA) or indirectly to quinolinic acid (QA) and picolinic acid (PIC). Finally, QA is metabolized into nicotinamide adenine dinucleotide (NAD⁺)

Previous studies point towards a role for the kynurenine pathway (KP; Fig. 1) as a common metabolic pathway involved in the pathophysiology of several neurological and psychiatric disorders, including AD, Parkinson’s disease, Huntington’s disease, schizophrenia, and major depressive disorder [4–7]. Furthermore, the KP has been implicated in autoimmune diseases such as multiple sclerosis, rheumatoid arthritis, and psoriasis [8–10], as well as in various cancers, including breast, lung, and colorectal cancer [11,12] and metabolic conditions such as type-2 diabetes [13].

Tryptophan (TRP) is an essential amino acid, supplied only through diet or supplements. The KP is the dominant pathway of TRP degradation, accounting for over 90% of TRP metabolism both in the periphery and the central nervous system [14]. Its downstream metabolites, collectively called kynurenines, have been associated with dementia-related pathophysiological processes such as immunoregulation, mitochondrial dysfunction, and oxidative stress [15–19], due to either neurotoxic (e.g., quinolinic acid) or neuroprotective (e.g., kynurenic acid) properties and interactions with N-methyl-d-aspartate (NMDA) receptors [20].

Studies in patients with dementia suggest that kynurenines are altered in blood [21–24] and cerebrospinal fluid (CSF) [5,22,25,26], with a tendency towards higher neurotoxic load (e.g., quinolinic levels), compared to cognitively healthy individuals. Recently, meta-analyses were published that consistently showed lower blood levels of TRP in patients with AD compared to healthy controls, while levels of kynurenine were not significantly different [27–29]. With respect to the other metabolites, results were mostly inconsistent or not enough studies were available to do a meta-analysis. Although these meta-analyses offer valuable insights into changes in kynurenines, they also do not specifically address the prodromal stage of AD, namely mild cognitive impairment (MCI), nor do they explore association of the KP with cognitive performance. MCI patients exhibit symptoms similar to those of AD, with the key distinction that these symptoms do not yet significantly impact daily functioning. As such, the role of the KP in cognitive impairment and dementia remains largely unclear. In this systematic review and meta-analysis, we investigated (1) differences in levels of TRP and kynurenines in patients with evident cognitive impairment compared to cognitively healthy controls, and (2) associations between metabolite levels and cognitive test scores.

## Methods

The current study is part of a larger systematic review in which associations of the KP with (a) age and (b) age-related neurodegenerative and neurocognitive disorders were investigated. Here, we report on the latter part of the study.

### Literature search

This systematic review and meta-analysis was performed according to the Preferred Reporting Items for Systematic reviews and Meta-Analyses (PRISMA) 2020 guidelines (Supplementary Table S11) [30,31]. A comprehensive systematic literature search was done including studies published until October 2023 using PubMed, Embase, PsychINFO, and the Cochrane Database of Systematic Reviews. The final search term was as follows: (tryptophan OR kynuren* OR anthranil* OR xanthurenic OR cinnabar* OR picolinic OR quinolinic) AND (Alzheim* OR dementia OR demented OR cogn* OR neurocogn* OR memory OR amnestic OR amnesia OR neuropsychol* OR aging). No filters were used during the search. Additionally, as part of a snowballing approach, reference lists of selected studies were screened, and some authors were contacted to provided extra references [32]. This study was registered in the International Prospective Register of Systematic Reviews (PROSPERO) database on March 11, 2020 (ID: CRD42020159274).

### Study selection

Two main reviewers (K.C. and L.B.) independently read and assessed the eligibility of the articles based on title and abstract using Endnote × 9 software, followed by assessment of the full text, where applicable. Differences in opinion were resolved through structured discussion or by consulting a third reviewer (S.K.). Data was extracted using a pre-specified data extraction form (Supplementary Appendix S1). Missing data were handled by contacting the corresponding author, and if the corresponding author did not reply after two reminders or could not provide the information, the paper was excluded from the systematic review and/ or meta-analysis. Out of 41 data requests, data for 10 articles (24.4%) were received (see acknowledgements).

### Study inclusion and exclusion

English full-length articles were included if they: 

1. Measured TRP or one of the KP metabolite(s): N-formylkynurenine, kynurenine (KYN), 3-hydroxykynurenine (3-HK), kynurenic acid (KA), xanthurenic acid (XA), anthranilic acid (AA), 3-hydroxyanthranilic acid (3-HAA), cinnabarinic acid (CA), picolinic acid (PIC), or quinolinic acid (QA);
2. Represented either a prospective cohort study, a cross-sectional study, or a case–control study;
3. Involved human participants and their biomaterials (CSF, plasma, serum, red blood cells, saliva, urine, feces, post-mortem tissues);
4. Included patients with subjective cognitive decline, MCI, or dementia (AD, vascular dementia, Creutzfeldt–Jakob disease, Lewy body dementia, frontotemporal dementia, Huntington’s disease, Parkinson’s disease, Korsakoff syndrome, mixed dementia), with or without neuropsychiatric symptoms (e.g., depression);
5. Compared patients to cognitively healthy control participants, compared patients with different levels of cognitive decline or different types of dementia, or investigated associations between metabolites and cognitive test scores in patients or controls.

Studies were excluded in dementia cases where a clinical dementia diagnosis was absent.

### Quality assessment

Study quality was assessed using the Newcastle–Ottawa scale (NOS) for case–control studies or cohort studies (Table S10) [33]. An adapted version was used for cross-sectional data and data comparing different patient groups (Appendix S2 and S3) [34]. The scales consist of three categories (selection, exposure, and comparability), of which a maximum of one star for each numbered item within the category (selection *n* = 4, exposure *n* = 2, comparability *n* = 3) can be awarded, nine in total.

### Meta-analysis

Meta-analyses were done in STATA 17.0 with the *metan* package. To investigate differences in metabolite levels between cases and controls, a random effect model was used that produced a standardized mean difference (SMD) and 95% confidence interval (CI). For this, the mean, standard deviation (SD), and sample size (*n*) were used. If a study reported standard error of the mean (SEM) instead, the SD was calculated using the following equation: SD = SEM $\sqrt[\ast]n$. Additionally, if a study reported the metabolites in weight/volume (e.g., µg/mL), the unit was converted to the correct molarity (µM or nM) using the molecular weight. Meta-analyses were only performed on metabolites reported in at least three independent articles. Effect size was interpreted using the guidelines from Cohen: small, SMD = 0.2; moderate, SMD = 0.5; and large, SMD = 0.8, with positive values indicating elevated metabolite levels in cases [35]. A *p* value < 0.05 was considered statistically significant in two-sided tests.

### Heterogeneity and publication bias

Heterogeneity of the included studies was assessed by *I*^2^ and Cochrane’s *Q* tests [36–38]. Since there is no golden standard in interpreting the *I*^2^ value, the Cochrane Handbook for Systematic Reviews of Interventions version 6.2, 2021 (Sect. 10.10.2) was used as a rough guideline [39]:

- 0 to 40%: might not be important;
- 30 to 60%: may represent moderate heterogeneity;
- 50 to 90%: may represent substantial heterogeneity;
- 75 to 100%: considerable heterogeneity

Small study bias was examined by funnel plots and Egger’s regression tests. A conservative *p* value < 0.1 was considered statistically significant, as previously described [40]. Bi- and multivariable meta-regressions were performed with metabolites reported in at least three independent studies with pooled estimates showing an *I*^2^ > 50%.

### Meta-regression

A Knapping–Hartung modification analysis was done to investigate potential sources of heterogeneity, including year of publication, sample size, and overall sample’s mean age (if not provided; this was calculated by formula =$\frac{n1\times1+n2\times2}{n1+n2}$, where ni is subsample (e.g., AD, controls) size and xi is the subsample’s mean age), sex (percentage female), biomaterial (CSF/blood, CSF/plasma/serum, or plasma/serum), analytical technique (high performance liquid chromatography (HPLC)/enzyme-linked immunosorbent assay (ELISA)/liquid chromatography with tandem mass spectrometry (LC–MS/MS)/others), dementia severity (mini-mental-state examination), recruitment of controls (volunteers/hospital controls/memory clinic or psychiatry department/community controls or partners/unknown or not reported), and, in plasma samples, type of anticoagulant tube (heparin/ethylenediaminetetraacetic acid (EDTA)/others). As insufficient information was available about educational level, kidney functioning, medication use, or comorbidities, assessment of these variables was not possible. Residual variation (*I*^2^_res_), adjusted (adj.) *R*^2^, tau^2^ (*τ*^2^), 2-tailed *p* value, *F* test *p* value (Prob. > *F*), and parameter estimates were reported.

### Certainty assessment

This review did not perform a certainty assessment using the Grading of Recommendations, Assessment, Development and Evaluations (GRADE) guideline because it does not represent frameworks for developing and presenting summaries for making clinical practice recommendations. Nevertheless, this study provides class II (moderately low risk) evidence.

## Results

### Characteristics of the included studies

**Fig. 2:**
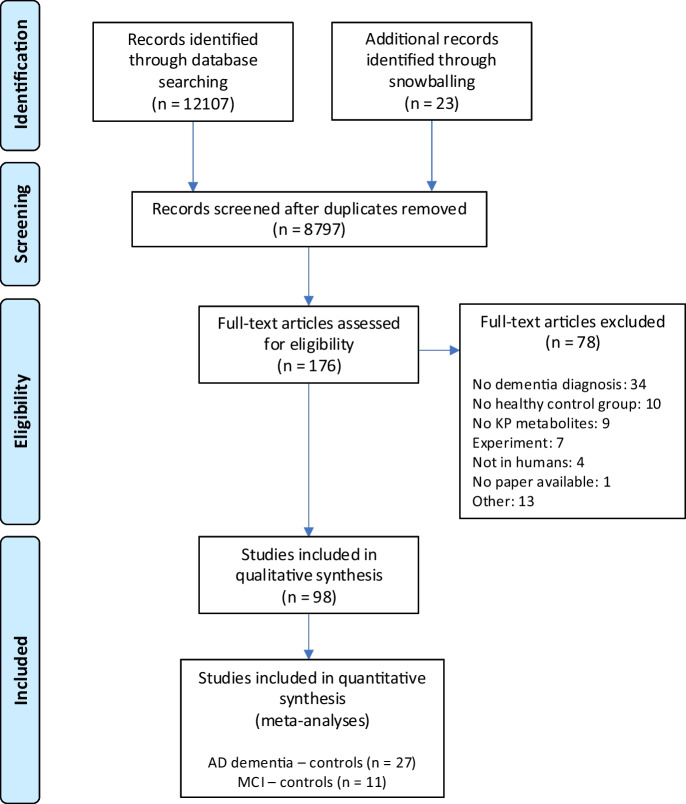
PRISMA flow diagram

After de-duplication, 8774 abstracts were retrieved from the search and an additional 23 studies were found through snowballing. A total of 176 full-text studies were assessed for eligibility, yielding 98 studies for the qualitative synthesis, of which 33 studies met the meta-analysis criteria (Fig. 2). Of these 33 studies, 27 were included in meta-analyses comparing individuals with AD dementia to controls, and 11 were included in meta-analyses comparing individuals with MCI to controls. Due to a broad search strategy, numerous studies were excluded. For instance, we excluded studies that centered on preclinical research, examined metabolites other than the KP, or constituted narrative reviews. Of the 98 studies included in the systematic review, 83 studies compared metabolite levels between patients with cognitive impairment or dementia and cognitively healthy controls, and 29 studies reported associations between kynurenines and cognitive test scores. These studies were not mutually exclusive.

Data included in this systematic review consisted of case–control data (*n* = 74), data comparing different patient groups (*n* = 3), cross-sectional data (*n* = 12), and prospective data (*n* = 9). In most studies, TRP or KYN were determined, with some studies investigating the downstream metabolites. A detailed overview of all the included studies in this systematic review is provided in supplementary Table S1.

### Systematic review

#### Systematic review of studies comparing different patient populations

Four studies compared metabolite levels between patients with different types of dementia or different levels of cognitive impairment, without including a cognitively healthy control group [41–44]. Two of these studies compared serum levels of patients with AD and Lewy body dementia using data from the same cohort [41,42]. Here, serum levels of 3-HK were higher in patients with Lewy body dementia, whereas levels of TRP or other metabolites (KYN, KA, XA, AA, 3-HAA, PIC, QA) or KTR were not significantly different [41,42]. Studies investigating CSF levels of TRP between patients with AD and vascular dementia [43], or between patients with AD and MCI [44], also observed no statistically significant differences.

#### Systematic review of prospective studies on dementia risk

There were six prospective studies investigating associations between kynurenines and risk of MCI and/or dementia [45–50] of which two studies both used data from the Alzheimer’s Disease Neuroimaging Initiative (ADNI) database [46,47]. Most of these studies investigated associations between metabolite levels and the risk for AD. In one study [45], an increase of one SD in AA plasma levels was associated with a 40% increased risk of incident all-type dementia and a 35% increased risk of AD dementia after a mean follow-up of 15.8 ± 5.2 years, while correcting for age, gender, education, APOE ε4 status, and total homocysteine levels. Higher levels of plasma KTR were associated with a greater risk of all-type dementia, but not of AD dementia. 3-HAA showed a tendency for an association with a lower risk of all-type dementia and AD dementia but did not reach statistical significance. No associations were found for TRP, KYN, KA, XA, or QA [45]. In another study, participants were followed-up for up to 12 years [49]. Here, after adjusting for age, gender, and APOE ε4 status, plasma levels of TRP, 3-HK, KA, 3-HAA, 3-HK/KYN, and 3-HAA/AA were higher in participants progressing to MCI or AD, whereas levels of PIC, QA, QA/KA, and PIC/QA were lower. The 3-HAA/AA ratio in particular had a large effect on the odds of a participant progressing, with one unit increase corresponding to a progression to MCI or AD being 35 times more likely. No associations were found with KYN, AA, or KTR in this study [49]. Other studies reported no significant associations between serum levels of TRP [46,48] and KYN [46,47] with risk of conversion to AD dementia after an average follow-up of 2 to 4.5 years [46–48]. No other KP metabolites were measured in these studies. Lastly, one study investigated associations between plasma kynurenines and post-stroke cognitive impairment (including cases of vascular dementia) in stroke patients who were followed up over a period of 3 years after stroke, but found no associations [50].

#### Systematic review of case–control studies in CSF and blood

##### Case–control studies measuring kynurenines in CSF

Case–control studies with kynurenines measured in CSF were found for AD dementia (*n* = 21 studies [5,22,25,26,51–67]), vascular dementia (*n* = 2 [52,64]), Lewy body dementia (*n* = 1 [53]), frontotemporal dementia (*n* = 3 [25,52,68]), and MCI (*n* = 4 [25,52,55,69]) (Tables 1 and S2). In studies comparing kynurenines between patients with AD dementia and cognitively healthy controls, TRP (*n* = 17 [5,22,25,26,51,52,54–59,61–65]), KYN (*n* = 9 [5,22,51,55,57,58,62,63,65]) and KA (*n* = 9 [5,22,25,26,51,53,60,63,66]) were, respectively in chronological order, the most commonly measured metabolites. The majority of studies with TRP and KYN reported non-significant differences [5,22,25,26,51,54–59,61–64], while only two studies reported significant lower levels of TRP [52,65] and KYN [65] in AD dementia. KA showed inconsistent associations when comparing AD dementia patients to controls, with most studies showing higher (*n* = 5 [22,25,26,51,60]) concentrations in patients, but other studies showing lower (*n* = 2 [5,63]) or no differences (*n* = 2 [53,66]). Other downstream kynurenines and ratios were less frequently studied. 3-HK levels were either found to be lower or not significantly different in AD dementia [5,22,51,62,65]. QA levels were higher in one study [26], lower in another study [51] and not significantly different from controls in all others [5,22,63,67]. One study reported lower levels of AA and higher levels of PIC [51]. All other studies investigating downstream kynurenines (XA, AA, 3-HAA, PIC) and KTR reported no significant differences between AD dementia patients and controls [5,22,26,55,58,65].

**Table 1:** Differences in CSF tryptophan and kynurenine metabolites between cases and controls

Study	Cases			Controls			Covariates in analyses	Metabolites									
	***N***	Age	% female	***N***	Age	% female		TRP	KYN	3-HK	KA	XA	AA	3-HAA	QA	PIC	KTR
***Alzheimer’s dementia***																	
Aquilani (2023)^[52]^	44	72.3 ± 7.6	47.7	15	73.6 ± 6.3	26.7	None	↓	-	-	-	-	-	-	-	-	-
Knapskog (2023)^[51]^	252	71 (66–75)^a^	59.1	105	71 (67–76)^a^	44.8	Age, sex, APOE ε4 status, Aβ_42,_ P-tau_181_	ns	ns	ns	↑	-	↓	-	↓	↑	-
González-Sánchez (2020)^[25]^	Moderate (20)	73.3 ± 7.2	65.0	23	64.7 ± 10.8	34.8	None	ns	-	-	↑	-	-	-	-	-	-
	Mild (41)	71.9 ± 8.1	53.7					ns	-	-	↑	-	-	-	-	-	-
van der Velpen (2019)^[26]^	40	74.9 ± 6.4	60.0	34	65.4 ± 6.2	67.7	None	ns	-	-	↑	-	ns	-	↑	-	-
Jacobs (2019)^[22]^	20	77.9 ± 7.5	55.0	18	73.1 ± 7.9	16.7	Sex	ns	ns	ns	↑	-	ns	ns	ns	ns	ns
Sorgdrager (2019)^[5]^	33	73.7 ± 6.0	54.5	39	71.3 ± 10.7	53.8	Age, sex	ns	ns	ns	↓	ns	-	-	ns	-	-
Wennström (2014)^[53]^	19	75.0	52.6	20	76	50.0	None	-	-	-	ns	-	-	-	-	-	-
Ibanez (2013)^[54]^	21	69 ± 9.6	71.4	21	58 ± 8.9	57.1	None	ns	-	-	-	-	-	-	-	-	-
Kaddurah-Daouk (2013)^[55]^	40	69.0	75.0	38	69.5	66.8	unk	ns	ns	-	-	-	-	-	-	-	ns
Trushina (2013)^[56]^	15	82.7 ± 4.2	20.0	15	78.6 ± 3.5	33.3	unk	ns	-	-	-	-	-	-	-	-	-
Czech (2012)^[57]^	Light to mild (MMSE > 22) (53)	69.7 ± 9.5	56.6	51	63.1 ± 7.7	52.9	Age, sex	ns	ns	-	-	-	-	-	-	-	-
	Moderate to strong (MMSE 14–22) (26)	69.6 ± 10.1	53.8					ns	ns	-	-	-	-	-	-	-	-
Kaddurah-Daouk (2011)^[58]^	15	80.0 ± 1.1	73.0	15	82.0 ± 8.8	73.0	Clinical/demographic measures, compound ratios	ns	ns	-	-	-	-	ns	-	-	-
Fonteh (2007)^[59]^	8	77.9 ± 7.4	50.0	8	79.5 ± 5.5	50.0	None	ns	-	-	-	-	-	-	-	-	-
Baran (1999)^[60]^	2	73.2	unk	5	72.3 ± 7.8	unk	None	-	-	-	↑	-	-	-	-	-	-
Molina (1998)^[61]^	37	70.9 ± 8.5	54.1	32	67.9 ± 9.2	53.1	None	ns	-	-	-	-	-	-	-	-	-
Tohgi (1995)^[62]^	15	68.0 ± 6.0	unk	10	68.5 ± 6.1	unk	None	ns	ns	↓	-	-	-	-	-	-	-
Heyes (1992)^[63]^	39	63.8 ± 1.2	unk	30	59.1 ± 14.2	unk	None	ns	ns	-	↓	-	-	-	ns	-	-
Martinez (1993)^[64]^	13	68.0 ± 6.0	69.2	15	66.0 ± 8.0	46.7	None	ns	-	-	-	-	-	-	-	-	-
Tohgi (1992)^[65]^	14	68.4 ± 10.1	unk	10	68.5 ± 6.1	unk	None	↓	↓	↓	-	-	-	-	-	-	ns
Beal (1990)^[66]^	9	76.7 ± 7.2	unk	50	43.8 ± 3.2	unk	None	-	-	-	ns	-	-	-	-	-	-
Mourdian (1989)^[67]^	35	64.0 ± 5.9	unk	23	65.0 ± 9.6	unk	None	-	-	-	-	-	-	-	ns	-	-
***Vascular dementia***																	
Aquilani (2023)^[52]^	8	75.5 ± 9.3	37.5	15	73.6 ± 6.3	26.7	None	↓	-	-	-	-	-	-	-	-	-
Martinez (1993)^[64]^	13	71.0 ± 6.0	46.2	15	66.0 ± 8.0	46.7	None	ns	-	-	-	-	-	-	-	-	-
***Lewy body dementia***																	
Wennström (2014)^[53]^	18	77.0	55.6	20	76	50.0	None	-	-	-	ns	-	-	-	-	-	-
***Frontotemporal dementia***																	
Aquilani (2023)^[52]^	13	69.9 ± 8.9	46.2	15	73.6 ± 6.3	26.7	None	↓	-	-	-	-	-	-	-	-	-
Janssens (2020)^[68]^	39	67.4 ± 11.6	48.7	25	67.3 ± 8.1	44.0	None	ns	ns	ns	ns	ns	ns	-	ns	ns	ns
González-Sánchez (2020)^[25]^	8	66.4 ± 5.2	37.4	23	64.7 ± 10.8	34.8	None	ns	-	-	ns	-	-	-	-	-	-
***All type dementia***																	
Bakker (2023)^[69]^	24	67.2 ± 6.4	50.0	SCD (66)	60.4 ± 8.6	22.7	Age, sex, educational level, eGFR	ns	ns	ns	ns	-	ns	ns	ns	ns	ns
***Mild cognitive impairment***																	
Aquilani (2023)^[52]^	10	71.0 ± 7.0	50.0	15	73.6 ± 6.3	26.7	None	↓	-	-	-	-	-	-	-	-	-
Bakker (2023)^[69]^	47	65.8 ± 8.0	34.0	SCD (66)	60.4 ± 8.6	22.7	Age, sex, educational level, eGFR	ns	ns	ns	ns	-	ns	ns	ns	ns	ns
González-Sánchez (2020)^[25]^	24	72.0 ± 7.1	58.4	23	64.7 ± 10.8	34.8	None	ns	-	-	↑	-	-	-	-	-	-
Ibanez (2013)^[54]^	MCI at follow-up (21)	60 ± 8.9	33.3	21	58 ± 8.9	57.1	None	↓	-	-	-	-	-	-	-	-	-
	AD at follow-up (12)	63 ± 9.4	50.0					↓	-	-	-	-	-	-	-	-	-
Kaddurah-Daouk (2013)^[55]^	36	69.9	52.8	38	69.5	66.8	unk	↓	↑	-	-	-	-	-	-	-	↑

↑Sig. higher in cases, ↓Sig. lower in cases, *ns* non-significant, *unk* unknown, - metabolite not measured. ^a^Age in median (IQR). *SCD* subjective cognitive decline, *TRP* tryptophan, *KYN* kynurenine, *KA* kynurenic acid, *AA* anthranilic acid, *3-HK* 3-hydroxykynurenine, *3-HAA* 3-hydroxyanthranilic acid, *XA* xanthurenic acid, *QA* quinolinic acid, *PIC* picolinic acid, *KTR* kynurenine-tryptophan ratio

Most studies in patients with other dementia disorders (vascular dementia, frontotemporal dementia, and Lewy body dementia) reported no significant differences in any of the measured metabolites compared to controls [25,52,53,64,68,69]. Some studies in MCI patients reported an increase in KYN [55], KA [25], or KTR [55] levels, as well as a decrease or no significant difference in TRP levels compared to controls [25,52,54,55,69]. Another study reported no significant difference in any of the measured metabolites [69].

Studies investigating differences in ratios of metabolites or neopterin between cases and controls were mostly non-significant [5,22,25,58,62,63,65,68,69] (Table S2).

##### Case–control studies measuring kynurenines in blood

Case–control studies investigating metabolite levels in blood (plasma/serum) consisted of patients with AD dementia (*n* = 36 [5,21–26,46,52,56,59–61,64,70–91]), vascular dementia (*n* = 2 [52,64]), Lewy body dementia (*n* = 1 [91]), frontotemporal dementia (*n* = 4 [52,68,91,92]), all type dementia (*n* = 5 [69,93–96]), MCI and AD dementia combined (*n* = 2 [97,98]), MCI (*n* = 14 [23–25,46,52,56,69,72,75,90,99–101]), and post-stroke cognitive impairment (*n* = 3 [50,102,103]) (Tables 2 and S3).

**Table 2:** Differences in plasma and serum kynurenine pathway metabolites between cases and controls

Study	Cases			Controls			Blood type	Covariates in analyses	Metabolites										
	***N***	Age	% female	***N***	Age	% female			TRP	N-f-KYN	KYN	3-HK	KA	XA	AA	3-HAA	QA	PIC	KTR
***Alzheimer’s dementia***																			
Liu (2023)^[74]^	37	83.1 ± 7.0	70.3	34	75.8 ± 7.4	50.0	S	Age	ns	-	-	-	-	-	-	-	-	-	-
Klatt (2021)^[73]^	28	Men 75.5^a^ ± 7.9 Women 73^a^ ± 8.5	71.4	93	Men 76^a^ ± 9.5 Women 74^a^ ± 8.6	47.3	S	Age, sex	ns	-	ns	ns	-	-	-	ns	-	-	-
Whiley (2021)^[23]^	103	76.5 ± 6.0	51.5	86	75.9 ± 5.2	48.8	S	None	↓	-	↓	ns	ns	↓	-	ns	ns	ns	ns
Willette (2021)^[24]^	112	74.8 ± 8.1	42.0	58	75.1 ± 5.8	48.3	S	Age, sex	↓	-	ns	-	-	-	-	-	-	-	ns
Sorgdrager (2019)^[5]^	33	73.7 ± 6.0	54.5	39	71.3 ± 10.7	53.8	S	Age, sex	ns	-	ns	ns	ns	↓	-	-	ns	-	-
Atukeren (2017)^[76]^	14	78.9 ± 8.0	42.9	32	77.3 ± 6.7	56.3	S	None	-	↑	↑	-	-	-	-	-	-	-	-
Oxenkrug (2017)^[77]^	20	unk	60.0	24	unk	50.0	S	None	ns	-	ns	↑	ns	ns	↓	-	-	-	ns
Toledo (2017)^[46]^	175	75.6	51.4	199	75.3	50.3	S	Age, gender, education, APOE ε4 status	ns	-	ns	-	-	-	-	-	-	-	-
González-Dominguez (2015a)^[78]^	23	79.2 ± 5.9	65.2	21	72.1 ± 5.4	57.1	S	None	↓	-	-	-	-	-	-	-	-	-	-
González-Dominguez (2015b)^[79]^	30	80.3 ± 5.0	60.0	30	73.5 ± 5.9	66.7	S	None	-	-	-	-	-	-	-	-	-	↑	-
González-Dominguez (2014)^[80]^	22	78.5 ± 5.0	54.6	18	70.7 ± 4.1	61.1	S	None	-	-	↑	-	-	-	-	-	-	-	-
Tsuruoka (2013)^[91]^	3	64.3 ± 16.9	0.0	9	68.1 ± 13.7	100	S	None	ns	-	-	-	-	-	-	-	-	-	-
Widner (2000)^[83]^	21	74.4 ± 5.4	71.4	20	73.4 ± 7.4	50.0	S	None	ns	-	ns	-	-	-	-	-	-	-	↑
Baran (1999)^[60]^	1	73.2	unk	4	72.3 ± 7.8	unk	S	None	-	-	-	-	↑	-	-	-	-	-	-
Widner (1999)^[84]^	24	unk	unk	unk	unk	unk	S	None	↓	-	ns	-	-	-	-	-	-	-	ns
Martinez (1993)^[64]^	13	68.0 ± 6.0	69.2	15	66.0 ± 8.0	46.7	S	None	ns	-	-	-	-	-	-	-	-	-	-
Aquilani (2023)^[52]^	44	72.3 ± 7.6	47.7	15	73.6 ± 6.3	26.7	P	None	ns	-	-	-	-	-	-	-	-	-	-
Xu (2021)^[72]^	Men (56)	unk	0.0	Men (128)	unk	0.0	P	Age, education, batch, APOE ε4 status	-	-	-	-	↓	↓	-	-	-	-	-
	Women (81)	unk	100	Women (155)	unk	100			-	-	-	-	ns	ns	-	-	-	-	-
González-Sánchez (2020)^[25]^	Moderate (20)	73.3 ± 7.2	65.0	23	64.7 ± 10.8	34.8	P	None	ns	-	-	-	ns	-	-	-	-	-	-
	Mild (41)	71.9 ± 8.1	53.7						ns	-	-	-	ns	-	-	-	-	-	-
Shao (2020)^[90]^	30	71.6 ± 8.8	66.7	43	65.5 ± 7.9	41.9	P	None	↓	-	-	-	-	-	-	-	-	-	-
Jacobs (2019)^[22]^	20	77.9 ± 7.5	55.0	18	73.1 ± 7.9	16.7	P	Sex	ns	-	ns	ns	ns	-	ns	↓	ns	ns	ns
Lin (2019)^[75]^	15	76.9 ± 8.0	unk	15	66.8 ± 6.5	unk	P	None	-	-	-	-	-	-	-	-	-	-	ns
van der Velpen (2019)^[26]^	40	74.9 ± 6.4	60.0	34	65.4 ± 6.2	67.7	P	None	↓	-	ns	ns	ns	-	-	-	ns	-	-
Giil (2017)^[21]^	42	78.5 ± 6.3	unk	42	78.6 ± 6.8	unk	P	Age, sex, creatinine	↓	-	↓	ns	ns	↓	ns	↓	↓	-	ns
Trushina (2013)^[56]^	15	82.7 ± 4.2	20.0	15	78.6 ± 3.5	33.3	P	unk	↓	-	-	-	-	-	-	-	-	-	-
Gulaj (2010)^[81]^	34	78.8 ± 5.7	70.6	18	76.2 ± 7.3	72.2	P	None	↓	-	ns	ns	↓	-	ns	-	↑	-	↑
Li, (2010)^[89]^	20	68 ± 10	50.0	20	70 ± 9	50.0	P	None	↓	-	-	-	-	-	-	-	-	-	-
Fonteh (2007)^[59]^	8	77.9 ± 7.4	50.0	8	79.5 ± 5.5	50.0	P	None	ns	-	-	-	-	-	-	-	-	-	-
Hartai (2007)^[82]^	28	77.0 ± 6.3	78.6	31	73 ± 8.3	67.7	P	None	-	-	ns	-	↓	-	-	-	-	-	-
Bonaccorso (1998)^[85]^	15	78.4 ± 10.3	80.0	15	75.6 ± 9.1	46.7	P	Age, sex	ns	-	-	-	-	-	-	-	-	-	-
Fekkes (1998)^[86]^	14	73.6 ± 6.3	71.4	17	70.1 ± 1.3	0	P	None	↓	-	-	-	-	-	-	-	-	-	-
Molina (1998)^[61]^	37	70.9 ± 8.5	54.1	32	67.9 ± 9.2	53.1	P	None	ns	-	-	-	-	-	-	-	-	-	-
Basun (1990)^[87]^	22	74.0 ± 9.0	59.1	11	79.0 ± 2.0	54.5	P	None	ns	-	-	-	-	-	-	-	-	-	-
Watkins (1989)^[88]^	22	77.3	68.2	22	76.0	68.2	P	None	↓	-	-	-	-	-	-	-	-	-	-
Schwarz (2013)^[71]^	20	74 ± 7.6	80.0	SCD (19)	59.5 ± 10.2	42.1	S	Age, sex	ns	-	ns	↑	ns	-	-	-	ns	ns	-
de Leeuw (2017)^[70]^	127	65.1 (9.1)^a^	50	SCD (121)	62.7 (8.0)^a^	46	P	Age, sex, clinical characteristics	ns	-	ns	-	-	-	-	-	-	-	-
***Vascular dementia***																			
Martinez (1993)^[64]^	13	71.0 ± 6.0	46.2	15	66.0 ± 8.0	46.7	S	None	↑	-	-	-	-	-	-	-	-	-	-
Aquilani (2023)^[52]^	8	75.5 ± 9.3	37.5	15	73.6 ± 6.3	26.7	P	None	ns	-	-	-	-	-	-	-	-	-	-
***Lewy body dementia***																			
Tsuruoka (2013)^[91]^	3	75.3 ± 4.9	33.3	9	68.1 ± 13.7	100	S	None	ns	-	-	-	-	-	-	-	-	-	-
***Frontotemporal dementia***																			
Janssens (2020)^[68]^	39	67.4 ± 11.6	48.7	26	67.0 ± 8.0	46.2	S	None	ns	-	ns	ns	ns	ns	ns	-	ns	ns	ns
Tsuruoka (2013)^[91]^	4	72.0 ± 2.9	0.0	9	68.1 ± 13.7	100	S	None	ns	-	-	-	-	-	-	-	-	-	-
Aquilani (2023)^[52]^	13	69.9 ± 8.9	46.2	15	73.6 ± 6.3	26.7	P	None	ns	-	-	-	-	-	-	-	-	-	-
Santos (2020)^[92]^	9	65.5 ± 9.5	33.3	15	67.7 ± 8.4	66.7	P	None	↓	-	-	-	-	-	-	-	-	-	-
***All type dementia***																			
Bakker (2023)^[69]^	24	67.2 ± 6.4	50.0	SCD (66)	60.4 ± 8.6	22.7	P	Age, sex, educational level, eGFR	ns	-	ns	ns	ns	ns	ns	ns	ns	ns	ns
Teruya (2021)^[93]^	8	84.6 ± 4.3	50.0	8	74.4 ± 4.5	50.0	P	None	↓	-	↑	-	-	-	-	-	↑	-	-
Rudman (1989)^[94]^	17	73.0	0.0	21	75.0	0.0	P	None	ns	-	-	-	-	-	-	-	-	-	-
Thomas (1986)^[95]^	23	77.2	60.9	23	76.1	60.9	P	None	↓	-	-	-	-	-	-	-	-	-	-
Shaw (1981)^[96]^	32	77.1	unk	70	70.1	unk	P	Sex	↓	-	-	-	-	-	-	-	-	-	-
***Mild cognitive impairment* + *Alzheimer’s dementia***																			
Rommer (2016)^[97]^	16	63.3 ± 13.7	56.3	15	62.8 ± 3.6	73.3	P	None	↓	-	ns	-	-	-	-	-	-	-	↑
Greilberger (2010)^[98]^	16	63.3 ± 13.7	56.3	15	62.8 ± 3.6	73.3	P	None	↓	-	ns	-	-	-	-	-	-	-	↑
***Mild cognitive impairment***																			
Whiley (2021)^[23]^	165	76.3 ± 6.0	57.0	86	75.9 ± 5.2	48.8	S	None	↓	-	↓	ns	ns	↓	-	ns	ns	ns	ns
Willette (2021)^[24]^	396	74.7 ± 7.4	35.4	58	75.1 ± 5.8	48.3	S	Age, sex	↓	-	ns	-	-	-	-	-	-	-	ns
Ramos-Chavez (2018)^[100]^	23	unk	unk	54	unk	unk	S	Age, TRP	↓	-	-	-	-	-	-	-	-	-	ns
Toledo (2017)^[46]^	356	75.1	64.6	199	75.3	50.3	S	Age, gender, education, APOE ε4 status	ns	-	ns	-	-	-	-	-	-	-	-
Aquilani (2023)^[52]^	10	71.0 ± 7.0	50.0	15	73.6 ± 6.3	26.7	P	None	ns	-	-	-	-	-	-	-	-	-	-
Bakker (2023)^[69]^	47	65.8 ± 8.0	34.0	SCD (66)	60.4 ± 8.6	22.7	P	Age, sex, educational level, eGFR	ns	-	ns	ns	ns	ns	ns	ns	ns	ns	ns
Ikeuchi (2022)^[99]^	219	79.5 ± 5.7	66.2	220	76.3 ± 6.6	53.2	P	None	ns	-	-	-	-	-	-	-	-	-	-
Xu (2021)^[72]^	Men (134)	unk	0.0	Men (128)	unk	0.0	P	Age, education, batch, APOE ε4 status	-	-	-	-	ns	ns	-	-	-	-	-
	Women (141)	unk	100	Women (155)	unk	100			-	-	-	-	ns	ns	-	-	-	-	-
González-Sánchez (2020)^[25]^	24	72.0 ± 7.1	58.4	23	64.7 ± 10.8	34.8	P	None	ns	-	-	-	ns	-	-	-	-	-	-
Peña-Bautista (2020)^[101]^	25	70 (67–73)^a^	60.0	25	66 (62–70)^a^	36.0	P	Age, gender	ns	-	-	-	-	-	-	-	-	-	-
Shao (2020)^[90]^	13	67.9 ± 7.2	38.5	43	65.5 ± 7.9	41.9	P	None	ns	-	-	-	-	-	-	-	-	-	-
Lin (2019)^[75]^	10	74.6 ± 8.5	unk	15	66.8 ± 6.5	unk	P	None	-	-	-	-	-	-	-	-	-	-	ns
Graham (2015)^[157]^	16	72.4 ± 7.3	50.0	37	73.1 ± 8.9	51.4	P	None	unk	unk	-	unk	-	-	-	-	-	-	-
Trushina (2013)^[56]^	15	80.4 ± 4.2	27.0	15	78.6 ± 3.5	33.3	P	unk	↑	-	-	-	-	-	-	-	-	-	-
***Post-stroke cognitive impairment***																			
Cogo (2021)^[102]^	13	69.4 ± 17.8	38.5	PSNCI (10)	64.7 ± 13.3	40.0	S	None	ns	↑	-	-	ns	-	-	-	↑	-	↑
Liu (2015)^[103]^	30	unk	unk	PSNCI (30)	unk	unk	S	None	↓	-	↑	-	-	-	-	-	-	-	-
Bakker (2023)^[50]^	127	65.0 ± 10.6	31.5	PSNCI (67)	66.0 ± 11.4	29.8	P	None	ns	-	ns	ns	ns	ns	ns	ns	ns	ns	ns

↑Sig. higher in cases, ↓Sig. lower in cases, *ns* non-significant, *unk* unknown, - metabolite not measured. ^a^Age in median (IQR). *SCD* subjective cognitive decline, *PSNCI* post-stroke no cognitive impairment, *P* plasma, *S* Serum, *TRP* tryptophan, *N-f-KYN* N-formyl-kynurenine, *KYN* kynurenine, *KA* kynurenic acid, *AA* anthranilic acid, *3-HK* 3-hydroxykynurenine, *3-HAA* 3-hydroxyanthranilic acid, *XA* xanthurenic acid, *QA* quinolinic acid, *PIC* picolinic acid, *KTR* kynurenine-tryptophan ratio

In studies comparing patients with AD dementia to healthy controls, TRP (*n* = 27 [5,21–26,46,52,56,59,61,64,73,74,77,78,81,83–91]) and KYN (*n* = 15 [5,21–24,26,46,73,76,77,80–84]) were again the most and second most commonly measured metabolites, respectively. Studies on TRP either reported no difference (*n* = 15 [5,22,25,46,52,59,61,64,73,74,77,83,85,87,91]) or lower levels (*n* = 12 [21,23,24,26,56,78,81,84,86,88–90]) in AD dementia. Results for KYN were inconsistent, with most studies reporting no significant difference (*n* = 11 [5,22,24,26,46,73,77,81–84]), whereas some reported higher (*n* = 2 [76,80]) or lower (*n* = 2 [21,23]) levels in AD dementia. Studies investigating differences in KA (*n* = 11 [5,21–23,25,26,60,71,77,81,82]) and QA (*n* = 6 [5,21–23,26,81]), the more commonly studied downstream metabolites, were inconclusive, although most studies reported no differences [5,22,23,25,26,71,77]. Interestingly, more consistent findings were obtained for many other downstream kynurenines (Tables 2 and S3). 3-HK, PIC, and KTR showed either higher levels in AD dementia or no significant difference [5,21–24,26,73,75,77,79,81,83,84], whereas for XA and AA, lower levels in AD dementia or also no significant difference were reported [5,21–23,77,81]. In two studies, 3-HAA levels were lower in AD dementia [21–23], whereas another study reported no differences [73]. One study investigated differences in levels of KA and XA separately for men and women and reported lower levels of these metabolites in men with AD dementia compared to controls, whereas no significant differences were reported for women [72].

Most of the studies in patients with other dementia disorders (vascular dementia, Frontotemporal dementia and Lewy body dementia) reported no significant differences in any of the measured metabolites compared to controls [52,64,68,69,91–96]. Some studies in MCI patients reported a decrease in TRP levels [23,24,100], one study reported an increase [56], and others reported no differences [25,46,52,69,90,99,101]. Studies investigating other metabolite levels or KTR generally reported no differences [23–25,46,69,72,75,100]. Lastly, studies investigating differences in ratios of metabolites or neopterin between cases and controls were mostly non-significant [5,13,21,22,25,50,68,69,71,73,81,84,97,98,100,102] (Table S3).

#### Systematic review of case–control studies measuring kynurenines in other tissues

Although KP metabolites have been studied the most in CSF or blood, other tissues have been investigated as well, including post-mortem brain tissue [67,104–111], red blood cells [82], saliva [91,112], urine [23,59,113,114], and fecal samples [115] (Table S4). A meta-analysis with these studies was not possible due to the limited number of studies.

##### Kynurenines in post-mortem brain tissue

Studies in post-mortem brain tissue mostly investigated differences in TRP levels between AD dementia patients and controls [104–110]. One study investigated differences in TRP, KYN, 3-HK, KA, XA, AA, PIC, QA, and KTR levels between individuals with AD dementia, frontotemporal dementia, and controls [111]. In this study, levels of 3-HK were lower in the medial and prefrontal cortex and posterior superior temporal cortex of individuals with AD dementia compared to controls. Levels of AA were higher in the premotor area and supplementary motor area, in the medial and prefrontal cortex, and in the substantia nigra of individuals with AD dementia compared to controls. These differences were no longer significant after Bonferroni correction. No differences were observed between individuals with frontotemporal dementia and controls [111]. Another study reported higher TRP levels in the hippocampus, entorhinal cortex, middle temporal gyrus, motor cortex, and cingulate gyrus of patients with AD dementia compared to controls [110], whereas other studies reported no differences in TRP levels in any of the examined brain areas [67,104–109,111]. Only one other study investigated differences in QA levels, but also did not find any differences [67].

##### Kynurenines in saliva

The two studies investigating differences in TRP levels in saliva were inconclusive [91,112]. One study reported that TRP levels were higher in AD dementia patients compared to MCI [112], while the other study reported no difference in TRP levels when comparing patients with AD dementia, frontotemporal dementia, or Lewy body dementia with controls [91].

##### Kynurenines in urine and fecal samples

Studies investigating urine levels of TRP in patients with AD dementia or MCI either report lower levels or no differences compared to controls [23,59,113,114]. One study also investigated downstream kynurenines and reported lower concentrations of KA and XA, and higher KTR in both AD dementia and MCI patients compared to controls, while urine levels of KYN, 3-HK and 3-HAA were not significantly different [23]. Lastly, one study investigated KP metabolites in fecal samples of patients with AD dementia or MCI and reported no differences in KYN or KA compared to controls [115].

#### Systematic review on associations of the kynurenine pathway with cognitive test scores

Associations between kynurenines and cognitive test scores have mostly been investigated in cross-sectional analyses (Tables S7-S9). Fifteen studies investigated associations between kynurenines and scores on the Mini-Mental State Examination (MMSE) [5,23,24,53,65,81,83,84,87,90,116–120], whereas 16 studies investigated associations with other cognitive tests or domains [24,46,47,67,81,87,118,119,121–128]. Most of these associations were non-significant. Several studies have examined associations between kynurenines and cognitive test scores over time as well, with follow-up periods ranging from 8 months to 5 years [41,42,48,50,125,129].

In healthy adults aged 18 to 65, no associations were found between plasma 3-HK, KA, QA, or the 3-HK/KA ratio and a global cognitive task score, which was derived from a combination of five standardized cognitive tasks administered at three time points (baseline, 4 and 8 months later) [129]. Among older adults, plasma TRP levels were associated with an increasing Montreal Cognitive Assessment (MoCA) score over 2 years, whereas higher concentrations of KYN were associated with a decline in the MoCA score after adjusting for age and sex [125]. No associations were observed with KTR or the KA/KYN ratio.

In participants without cognitive impairment, MCI and AD, serum TRP levels were not associated with changes in cognitive functioning over time (including global cognitive functioning, episodic memory, working memory, semantic member, perceptual speed, and visuospatial ability) during an average follow-up time of 4.5 years, independent of age, sex, education, and batch [48]

Two studies used data from patients with AD and Lewy body dementia from the same cohort [41,42]. In these studies, both low and high serum levels of KYN were non-linearly associated with lower MMSE scores over 5 years [41]. No associations were found with TRP, 3-HK, KA, XA, AA, 3-HAA, PIC, QA, KTR, or the KA/KYN ratio [41,42].

Lastly, in participants after stroke, higher plasma levels of AA were associated with better episodic memory at baseline. Additionally, a linear-quadratic association was found for KA/QA with episodic memory. Higher levels of KA were associated with better processing speed in women only. These associations did not change during the 3 year follow-up [50]. No associations were found with TRP, KYN, 3-HK, XA, 3-HAA, PIC, QA, or KTR in this study, nor with the executive functioning domain [50].

### Meta-analysis

#### Meta-analysis of case–control studies

Since the amount of cross-sectional and prospective data was too small, meta-analyses were only performed on case–control data with kynurenines measured in CSF and/or blood. Some studies ([97,98] and [88,95]) used data from the same participants; therefore, studies were included in the meta-analysis based on year of publication [98] and availability of overall estimates for patients and controls [88].

#### Meta-analyses on differences in metabolite levels between individuals with AD dementia and controls

**Fig. 3:**
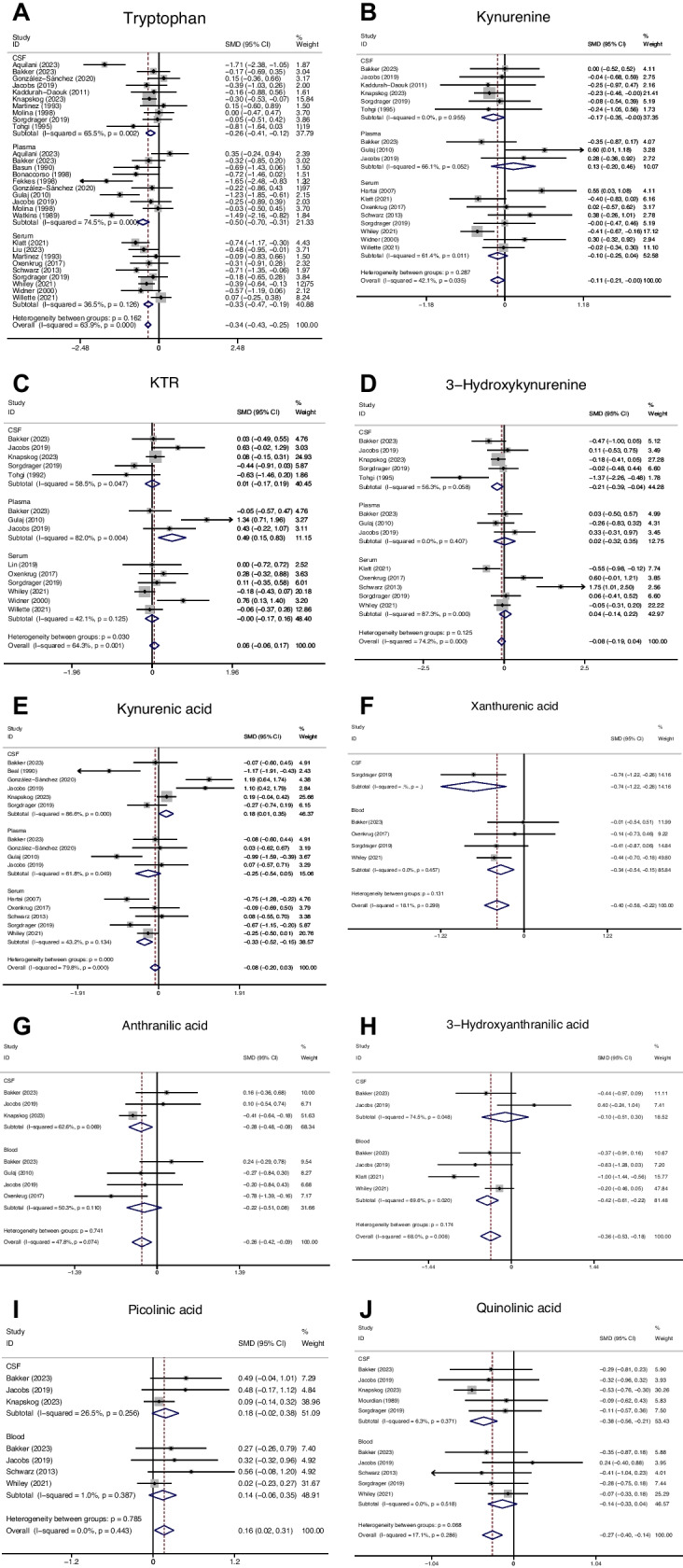
Forest plots of AD-control studies

**Fig. 4:**
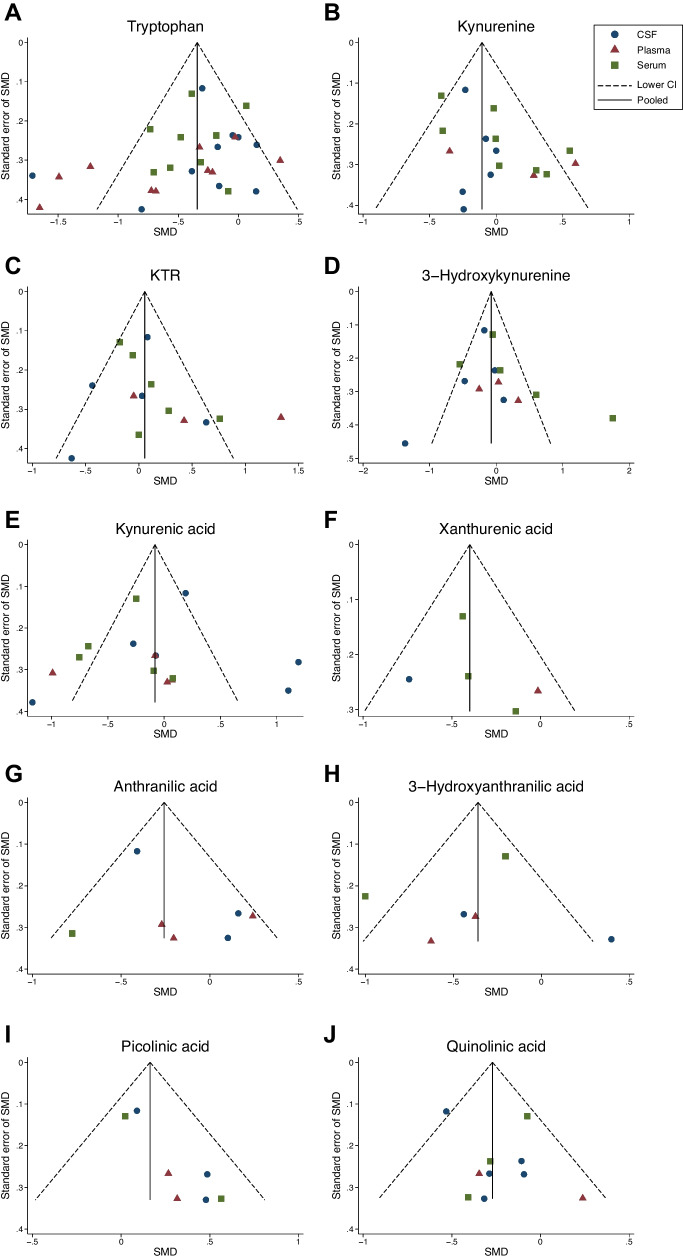
Funnel plots of AD-control studies

Twenty-seven studies were included in meta-analyses investigating differences in kynurenines between patients with AD dementia and controls (Table 3; Figs. 3 and 4). In the overall analyses (CSF and blood combined), TRP, KYN, XA, AA, 3-HAA, and QA were all lower in AD dementia patients compared to controls, while PIC was higher. Overall levels of 3-HK, KA, and KTR were not different between AD dementia patients and controls.

**Table 3:** Effect sizes and Egger’s bias coefficients of studies investigating differences between patients with AD and controls

		**Effect size**		**Heterogeneity**		**Publication bias**	
	***N***	**SMD (95% CI)**^**a**^	*p *value	***I***^***2***^** (%)**	*p *value	**Egger’s bias coefficient**	*p *value
**Tryptophan**							
Overall	29	− 0.34 (− 0.43, − 0.25)	** < 0.001**	63.9	** < 0.001**	− 1.40	0.103
CSF	10	− 0.26 (− 0.41, − 0.12)	** < 0.001**	65.5	**0.002**	− 0.56	0.699
Blood	19	− 0.39 (− 0.51, − 0.28)	** < 0.001**	63.9	** < 0.001**	− 1.95	**0.092**
Plasma	10	− 0.50 (− 0.70, − 0.31)	** < 0.001**	74.5	** < 0.001**	− 7.38	**0.051**
Serum	9	− 0.33 (− 0.47, − 0.19)	** < 0.001**	36.5	0.126	− 0.90	0.505
**Kynurenine**							
Overall	17	− 0.11 (− 0.21, − 0.00)	**0.048**	42.1	**0.035**	1.92	**0.019**
CSF	6	− 0.17 (− 0.35, 0.00)	0.050	0.0	0.955	0.46	0.330
Blood	11	− 0.07 (− 0.20, 0.07)	0.325	61.0	**0.004**	3.42	**0.014**
Plasma	3	0.13 (− 0.20, 0.46)	0.447	66.1	0.052	12.19	0.496
Serum	8	− 0.10 (− 0.25, 0.04)	0.159	61.4	**0.011**	3.50	**0.033**
**KTR**							
Overall	14	0.06 (− 0.06, 0.17)	0.335	64.3	**0.001**	1.50	0.189
CSF	5	0.01 (− 0.17, 0.19)	0.928	58.5	**0.047**	− 0.78	0.683
Blood	9	0.09 (− 0.06, 0.24)	0.240	69.6	**0.001**	3.37	**0.030**
Plasma	3	0.49 (0.15, 0.83)	**0.005**	82.0	**0.004**	14.62	0.473
Serum	6	− 0.00 (− 0.17, 0.16)	0.960	42.1	0.125	2.55	**0.060**
**3-Hydroxykynurenine**							
Overall	13	− 0.08 (− 0.19, 0.04)	0.213	74.2	** < 0.001**	1.05	0.466
CSF	5	− 0.21 (− 0.39, − 0.04)	**0.019**	56.3	0.058	− 1.33	0.448
Blood	8	0.04 (− 0.13, 0.19)	0.670	78.9	** < 0.001**	3.10	0.178
Plasma	3	0.02 (− 0.32, 0.35)	0.922	0.0	0.407	5.75	0.624
Serum	5	0.04 (− 0.14, 0.22)	0.666	87.3	** < 0.001**	4.37	0.243
**Kynurenic acid**							
Overall	15	− 0.08 (− 0.20, 0.03)	0.167	79.8	** < 0.001**	− 0.60	0.704
CSF	6	0.18 (0.01, 0.35)	**0.037**	86.6	** < 0.001**	− 0.11	0.973
Blood	9	− 0.31 (− 0.47, − 0.15)	** < 0.001**	47.1	0.057	0.03	0.986
Plasma	4	− 0.25 (− 0.54, 0.05)	0.104	61.8	**0.049**	0.27	0.983
Serum	5	− 0.33 (− 0.52, − 0.15)	** < 0.001**	43.2	0.134	− 0.40	0.847
**Xanthurenic acid**							
Overall	5	− 0.40 (− 0.58, − 0.22)	** < 0.001**	18.1	0.299	1.07	0.563
CSF	1	-	-	-	-	-	-
Blood	4	− 0.34 (− 0.54, − 0.15)	**0.001**	0.0	0.457	1.89	0.196
Plasma	1	-	-	-	-	-	-
Serum	3	− 0.40 (− 0.61, − 0.19)	** < 0.001**	0.0	0.658	1.26	0.372
**Anthranilic acid**							
Overall	7	− 0.26 (− 0.42, − 0.09)	**0.002**	47.8	0.074	1.44	0.301
CSF	3	− 0.28 (− 0.48, − 0.08)	**0.007**	62.6	0.069	3.02	0.160
Blood	4	− 0.22 (− 0.51, 0.08)	0.148	50.3	0.110	− 12.81	0.283
Plasma	3	− 0.05 (− 0.39, 0.28)	0.757	0.0	0.379	− 8.10	0.482
Serum	1	-	-	-	-	-	-
**3-Hydroxyanthranilic acid**							
Overall	6	− 0.36 (− 0.53, − 0.18)	** < 0.001**	68.0	**0.008**	− 0.64	0.789
CSF	2	-	-	-	-	-	-
Blood	4	− 0.42 (− 0.61, − 0.22)	** < 0.001**	69.6	**0.020**	− 2.70	0.380
Plasma	2	-	-	-	-	-	-
Serum	2	-	-	-	-	-	-
**Picolinic acid**							
Overall	7	0.16 (0.02, 0.31)	**0.024**	0.0	0.443	1.96	**0.004**
CSF	3	0.18 (− 0.02, 0.38)	0.072	26.5	0.256	2.14	0.129
Blood	4	0.14 (− 0.06, 0.35)	0.167	1.0	0.387	2.03	**0.052**
Plasma	2	-	-	-	-	-	-
Serum	2	-	-	-	-	-	-
**Quinolinic acid**							
Overall	10	− 0.27 (− 0.40, − 0.14)	** < 0.001**	17.1	0.286	0.87	0.369
CSF	5	− 0.38 (− 0.56, − 0.21)	** < 0.001**	6.3	0.371	2.00	**0.075**
Blood	5	− 0.14 (− 0.33, 0.04)	0.130	0.0	0.518	− 0.64	0.629
Plasma	2	-	-	-	-	-	-
Serum	3	− 0.15 (− 0.36, 0.06)	0.153	0.0	0.534	− 1.78	**0.040**

^a^SMD > 0 trend towards higher in AD, SMD < 0 trend towards lower in AD compared to controls. - Insufficient observations. Overall presents CSF and blood combined, and Blood presents plasma and serum combined. Statistically significant *p*-values are in bold. *SMD* standardized mean difference, *KTR* kynurenine-tryptophan ratio, *CSF* cerebrospinal fluid, *CI* confidence interval

Subsequently, subgroup analyses were done while stratifying for biofluid type (CSF and blood). CSF levels were lower for TRP, 3-HK, AA, and QA, while levels of KA were higher in AD dementia patients. For some metabolites (KYN, PIC), and KTR CSF levels were not different, while for other metabolites (XA, 3-HAA) not enough independent articles were available to conduct a meta-analysis.

Blood levels of TRP, KA, XA, and 3-HAA were all reported to be lower in patients with AD dementia. Blood levels of other metabolites (KYN, 3-HK, AA, PIC, QA) and KTR were not different. Separate analyses for plasma and serum consistently showed that levels of TRP were lower in patients with AD dementia in both plasma and serum. Additionally, serum levels of KA and XA were lower in patients with AD dementia as well. Plasma levels of KTR were higher. No differences were found for KYN or 3-HK in either plasma or serum. Additionally, no differences were found for KA or AA in plasma or for QA or KTR in serum. Not enough studies were available to do a separate analysis for XA, 3-HAA, PIC, and QA in plasma or for AA, 3-HAA, and PIC in serum.

#### Meta-analyses on differences in metabolite levels between individuals with MCI and controls

Eleven studies were included in meta-analyses investigating differences in kynurenines between patients with AD dementia and controls (Table S5 and Figures S4-S7). Overall levels of TRP were lower in individuals with MCI compared to controls, while levels of KTR and KA were higher. No differences were found for KYN, 3-HK, AA, 3-HAA, PIC, or QA. CSF levels of KA and KTR were higher, while CSF levels of TRP and KYN were not significantly different. Blood levels of TRP were lower, while levels of KTR were higher. Blood levels of KYN and 3-HK were not different. Other metabolites had insufficient or no articles to conduct a separate meta-analysis for CSF and/or blood.

### Meta-regression

Studies comparing levels of TRP, KYN, KTR, 3-HK, KA, AA, and 3-HAA between patients with AD and controls were included as these showed considerable heterogeneity (*I*^2^ > 50%) (Table 4). Sources of heterogeneity were found in studies investigating overall metabolite levels and in blood and serum specifically (Table 4). The covariate that most frequently showed significance was “year of publication”. Results from the meta-regression showed that the publication year had an influence on the estimates for KA (overall), for TRP, KYN, KTR (in blood) and for KYN, 3-HK (in serum). Analytical technique (HPLC versus ELISA and LC–MS versus ELISA) had an influence on the estimates for 3-HK (overall and in blood). Lastly, sample size (in blood and serum) and sex (in blood) were sources of heterogeneity for KYN. With respect to studies comparing patients with MCI and controls, levels of TRP, KYN, KTR, KA, AA, and 3-HAA were included in a meta-regression, but no sources of heterogeneity could be identified (Table S6).

**Table 4:** Summary of Meta-regression on studies investigating differences between patients with AD and controls

	**Residual variation (*I***^**2**^_**res**_**) (%)**	**Adj. *R***^**2**^** (%)**	***τ***^**2**^	***P* >|*t*|**	**Prob. > *F***
**Tryptophan**					
Overall (*n* = 29)					
Year of publication	63.66	2.91	0.143	0.217	-
Sample size	64.58	− 4.25	0.154	0.373	-
Age *(n* = *28)*	64.53	5.30	0.149	0.188	-
Sex (% female) *(n* = *28)*	64.59	− 0.63	0.152	0.473	-
Dementia severity *(n* = *18)*	66.69	− 12.62	0.162	0.905	-
Recruitment of controls	65.83	− 7.73	0.159	-	0.569
Biomaterial					
CSF/blood	64.44	− 1.84	0.150	0.393	-
CSF/plasma/serum	64.88	− 4.95	0.155	-	0.475
Analytical technique	63.29	2.08	0.145	-	0.361
CSF (*n* = 10)					
Year of publication	67.57	− 11.86	0.199	0.541	-
Sample size	69.23	− 28.83	0.229	0.904	-
Age	68.51	− 16.48	0.207	0.690	-
Sex (% female) *(n* = *9)*	69.69	− 9.13	0.200	0.452	-
Dementia severity *(n* = *7)*	80.32	− 26.29	0.429	0.741	-
Recruitment of controls	72.70	− 43.82	0.256	-	0.739
Analytical technique	62.50	9.62	0.161	-	0.415
Blood (*n* = 19)					
Year of publication	58.63	25.03	0.104	**0.048**	-
Sample size	62.45	4.61	0.132	0.201	-
Age *(n* = *18)*	66.28	0.74	0.151	0.276	-
Sex (% female)	57.91	24.69	0.104	0.125	-
Dementia severity *(n* = *11)*	59.92	− 28.78	0.096	0.909	-
Recruitment of controls	58.63	28.60	0.099	-	0.124
Biomaterial					
Plasma/serum	64.56	− 4.48	0.144	0.378	-
Analytical technique	66.69	− 17.92	0.163	-	0.681
Plasma (*n* = 10)					
Year of publication	68.58	33.22	0.209	0.073	-
Sample size	68.97	25.18	0.234	0.115	-
Age	72.08	10.01	0.281	0.236	-
Sex (% female)	72.76	6.35	0.292	0.310	-
Dementia severity *(n* = *5)*	75.27	− 21.21	0.288	0.520	-
Recruitment of controls	43.19	74.22	0.080	-	0.092
Analytical technique	82.24	− 56.38	0.488	-	0.916
Anticoagulant	78.62	− 33.11	0.416	-	0.917
**Kynurenine**					
Blood (*n* = 11)					
Year of publication	11.67	83.42	0.014	**0.008**	-
Sample size	26.42	68.48	0.026	**0.020**	-
Age *(n* = *10)*	68.69	− 16.06	0.110	0.590	-
Sex (% female)	46.95	44.55	0.046	**0.049**	-
Dementia severity *(n* = *7)*	74.45	− 13.16	0.128	0.469	-
Recruitment of controls	66.90	− 24.88	0.103	**-**	0.692
Biomaterial					
Plasma/serum	62.58	− 8.50	0.089	0.597	-
Analytical technique	65.05	− 2.35	0.084	-	0.317
Plasma (*n* = 3)					
Year of publication	0.00	100.00	0.000	0.265	-
Sample size	42.65	57.45	0.069	0.382	-
Age	0.00	100.00	0.000	0.252	-
Sex (% female)	15.38	89.60	0.017	0.300	-
Dementia severity	-	-	-	-	-
Recruitment of controls	-	-	-	-	-
Analytical technique	-	-	-	-	-
Anticoagulant	-	-	-	-	-
Serum (*n* = 8)					
Year of publication	17.16	78.91	0.016	**0.031**	-
Sample size	24.62	70.42	0.022	**0.045**	-
Age *(n* = *7)*	57.48	27.85	0.066	0.245	-
Sex (% female)	42.61	48.80	0.038	0.068	-
Dementia severity *(n* = *5)*	79.09	− 46.32	0.150	0.902	-
Recruitment of controls	68.16	− 39.72	0.105	-	0.795
Analytical technique	68.32	− 26.64	0.095	-	0.584
**KTR**					
Overall (*n* = 14)					
Year of publication	63.97	2.09	0.123	0.530	-
Sample size	65.98	− 8.40	0.136	0.441	-
Age *(n* = *13)*	68.44	0.90	0.141	0.183	-
Sex (% female) *(n* = *12)*	67.55	5.52	0.125	0.217	-
Dementia severity *(n* = *8)*	75.12	− 20.14	0.237	0.582	-
Recruitment of controls	70.43	− 30.71	0.165	-	0.557
Biomaterial					
CSF/blood	66.62	− 5.61	0.133	0.295	-
CSF/plasma/serum	62.54	8.35	0.115	-	0.274
Analytical technique	-	-	-	-	-
HPLC/ELISA	64.98	17.35	0.104	0.114	-
CSF (*n* = 5)					
Year of publication	56.41	19.46	0.075	0.336	-
Sample size	65.53	− 94.50	0.181	0.777	-
Age	67.70	− 68.86	0.157	0.658	-
Sex (% female) *(n* = *4)*	69.53	− 65.06	0.132	0.555	-
Dementia severity *(n* = *3)*	0.00	100.00	0.000	0.361	-
Recruitment of controls	-	-	-	-	-
Analytical technique	-	-	-	-	-
LC–MS/ ELISA	67.73	− 54.44	0.144	0.753	-
Blood (*n* = 9)					
Year of publication	26.85	93.61	0.009	**0.011**	-
Sample size	49.00	56.13	0.065	0.083	-
Age *(n* = *8)*	76.50	− 9.13	0.195	0.364	-
Sex (% female) *(n* = *8)*	66.01	43.69	0.097	0.075	-
Dementia severity *(n* = *5)*	83.76	− 33.12	0.357	0.645	-
Recruitment of controls	75.15	− 31.30	0.195	-	0.693
Biomaterial					
Plasma/serum	64.53	15.32	0.126	0.238	-
Analytical technique	-	-	-	-	-
HPLC/ELISA	71.96	7.49	0.138	0.184	-
Plasma (*n* = 3)					
Year of publication	0.00	100.00	0.000	0.186	-
Sample size	85.47	− 47.71	0.603	0.631	-
Age	63.48	55.13	0.183	0.352	-
Sex (% female)	0.00	100.00	0.000	0.187	-
Dementia severity	-	-	-	-	-
Recruitment of controls	-	-	-	-	-
Analytical technique	-	-	-	-	-
Anticoagulant	-	-	-	-	-
**3-Hydroxykynurenine**					
Overall (*n* = 13)					
Year of publication	76.34	− 9.63	0.312	0.446	-
Sample size	75.15	− 11.52	0.317	0.584	-
Age *(n* = *12)*	75.96	− 18.35	0.333	0.905	-
Sex (% female) *(n* = *12)*	73.63	− 12.02	0.226	0.525	-
Dementia severity *(n* = *8)*	82.31	− 25.47	0.633	0.979	-
Recruitment of controls	76.79	− 10.31	0.314	-	0.375
Biomaterial					
CSF/blood	74.04	7.34	0.264	0.175	-
CSF/plasma/serum	76.39	− 5.67	0.301	-	0.356
Analytical technique	52.33	84.60	0.044	-	**0.017**
HPLC/ELISA	-	-	-	**0.010**	-
LC–MS/ELISA	-	-	-	**0.005**	-
Other/ELISA	-	-	-	-	-
CSF (*n* = 5)					
Year of publication	34.92	79.18	0.014	0.162	-
Sample size	66.07	− 223.51	0.217	0.713	-
Age	49.22	100.00	0.000	0.290	-
Sex (% female) *(n* = *4)*	0.10	-	0.000	0.579	-
Dementia severity *(n* = *3)*	85.09	− 172.84	0.795	0.964	-
Recruitment of controls	-	-	-	-	-
Analytical technique	-	-	-	-	-
LC–MS/ELISA	65.33	− 147.51	0.166	0.622	-
Blood (*n* = 8)					
Year of publication	79.19	− 1.43	0.346	0.362	-
Sample size	78.14	12.25	0.299	0.218	-
Age *(n* = *7)*	79.98	3.24	0.370	0.321	-
Sex (% female)	81.93	− 22.18	0.417	0.791	-
Dementia severity *(n* = *5)*	86.07	− 44.24	0.697	0.835	-
Recruitment of controls	-	-	-	-	-
Biomaterial					
Plasma/serum	81.94	− 19.45	0.407	0.628	-
Analytical technique	50.39	81.67	0.063	-	0.032
HPLC/ELISA	-	-	-	**0.020**	-
LC–MS/ELISA	-	-	-	**0.012**	-
Other/ELISA	-	-	-	-	-
Serum (*n* = 5)					
Year of publication	36.39	94.51	0.034	**0.020**	-
Sample size	87.01	23.49	0.480	0.237	-
Age *(n* = *4)*	84.29	73.48	0.223	0.118	-
Sex (% female)	79.26	71.92	0.176	0.065	-
Dementia severity *(n* = *3)*	95.00	− 114.47	1.892	0.898	-
Recruitment of controls	-	-	-	-	-
Analytical technique	72.77	77.00	0.144	-	0.161
**Kynurenic acid**					
Overall (*n* = 15)					
Year of publication	72.83	37.09	0.195	**0.023**	-
Sample size	79.74	− 9.75	0.341	0.689	-
Age *(n* = *14)*	82.68	− 6.32	0.364	0.469	-
Sex (% female) *(n* = *14)*	77.54	15.96	0.225	0.112	-
Dementia severity *(n* = *10)*	78.15	− 2.25	0.278	0.384	-
Recruitment of controls	75.69	18.60	0.253	-	0.194
Biomaterial					
CSF/blood	75.15	13.68	0.268	0.162	-
CSF/plasma/serum	76.96	3.66	0.299	-	0.385
Analytical technique	78.00	7.59	0.287	-	0.372
CSF (*n* = 6)					
Year of publication	84.93	33.36	0.428	0.153	-
Sample size	89.24	− 33.08	0.854	0.993	-
Age	82.04	52.44	0.305	0.080	-
Sex (% female) *(n* = *5)*	87.32	− 42.14	0.522	0.973	-
Dementia severity *(n* = *3)*	93.73	− 101.24	1.119	0.844	-
Recruitment of controls	86.91	9.96	0.578	-	0.387
Analytical technique	86.91	− 10.69	0.711	-	0.545
Plasma (*n* = 4)					
Year of publication	0.00	100.00	0.000	0.128	-
Sample size	71.81	− 60.53	0.246	0.717	-
Age	64.80	− 20.39	0.185	0.489	-
Sex (% female)	25.47	77.27	0.035	0.191	-
Dementia severity *(n* = *3)*	70.48	− 13.86	0.244	0.527	
Recruitment of controls	-	-	-	-	-
Analytical technique	-	-	-	-	-
Anticoagulant	64.65	− 15.94	0.178	0.490	-
**Anthranilic acid**					
CSF (*n* = 3)					
Year of publication	74.05	− 51.15	0.121	0.704	-
Sample size	0.00	100.00	0.000	0.262	-
Age	76.07	− 74.10	0.139	0.787	-
Sex (% female	0.00	100.00	0.000	0.263	-
Dementia severity	-	-	-	-	-
Recruitment of controls	-	-	-	-	-
Analytical technique	-	-	-	-	-
Blood (*n* = 4)					
Year of publication	54.92	− 26.44	0.115	0.515	-
Sample size	9.67	96.06	0.004	0.206	-
Age *(n* = *3)*	0.00	100.00	0.000	0.396	-
Sex (% female)	44.92	14.92	0.078	0.386	-
Dementia severity	-	-	-	-	-
Recruitment of controls	-	-	-	**-**	-
Biomaterial					
Plasma/serum	0.00	94.72	0.005	0.187	-
Analytical technique	-	-	-	-	-
**3-Hydroxyanthranilic acid**					
Overall (*n* = 6)					
Year of publication	73.05	− 24.78	0.160	0.615	-
Sample size	74.05	− 45.01	0.186	0.757	-
Age	73.85	− 40.22	0.180	0.873	-
Sex (% female)	73.83	− 30.36	0.167	0.596	-
Dementia severity *(n* = *3)*	0.00	-	0.000	0.539	-
Recruitment of controls	-	-	-	-	-
Biomaterial					
CSF/blood	70.97	2.92	0.124	0.290	-
CSF/plasma/serum	78.07	− 42.87	0.183	-	0.596
Analytical technique	-	-	-	-	-
HPLC/ELISA	55.48	34.90	0.083	0.228	-
Blood (*n* = 4)					
Year of publication	79.14	− 40.02	0.136	0.739	-
Sample size	67.89	− 22.72	0.119	0.592	-
Age	79.56	− 58.83	0.155	0.839	-
Sex (% female)	79.63	− 53.19	0.149	0.735	-
Dementia severity	-	-	-	-	-
Recruitment of controls	-	-	-	-	-
Biomaterial					
Plasma/serum	79.52	− 61.25	0.157	0.887	-
Analytical technique	-	-	-	-	-

^- ^Insufficient observations or not applicable. Meta regression with Knapp–Hartung modification was done on metabolites with higher than 50% heterogeneity (*I*^*2*^). Overall presents kynurenines in CSF and blood combined, and blood presents kynurenines in plasma and serum combined. MMSE scores were used as a proxy for dementia severity. Statistically significant *p* values (2-tailed or *F* test) are in bold *CSF* Cerebrospinal fluid, *LC–MS/MS* liquid chromatography with tandem mass spectrometry, *ELISA* enzyme-linked immunosorbent assay, *HPLC* high performance liquid chromatography, *CSF* cerebrospinal fluid, *adj.* adjusted, *τ*^*2*^*,* tau squared

## Discussion

This systematic review and meta-analysis identified and summarized findings from 98 studies that investigated KP metabolites in patients with cognitive impairment or dementia, or examined associations of KP metabolites with cognitive test scores. Twenty-seven articles were included in meta-analyses comparing TRP and kynurenines between patients with AD dementia and cognitively healthy controls. Results showed that patients with AD dementia had lower CSF levels of TRP, 3-HK, QA, and AA, while CSF levels of KA were higher. Blood levels of TRP, KA, XA, and 3-HAA were lower, whereas CSF levels of KYN and PIC, as well as blood levels of KYN, 3-HK, QA, AA, and PIC showed no significant differences. Similar trends were observed in meta-analyses comparing individuals with MCI and controls, although fewer studies were available. Studies investigating associations between kynurenines and cognitive tests generally reported non-significant findings. These findings strengthen previous observations of changes in various kynurenines in cognitive impairment and AD, but also challenge existing hypotheses regarding the role of several others.

### Lower central levels of QA in patients with AD dementia

We observed lower CSF levels of QA in patients with AD dementia, with similar but non-significant trends for blood QA levels. Multiple lines of evidence have suggested QA’s involvement in AD pathophysiology, linking it to processes such as oxidative stress, glutamatergic excitotoxicity, neuroinflammation, and mitochondrial dysfunction [130–134]. In post-mortem brain tissue, QA has been associated with AD’s neuropathological features. For example, QA expression was found to be highest in the perimeter of senile plagues in the hippocampus [135,136], and QA was shown to increase tau phosphorylation in human primary neurons [137]. In turn, amyloid beta (Aβ)_1–42_ was shown to significantly increase the production of QA by microglia and human primary macrophages [138,139], at least in a cell culture model.

However, studies investigating QA levels in different brain regions of patients with AD pathology and controls generally found no differences [67,111,140]. Results from longitudinal observational studies also do not support a role for QA in the development of AD. One longitudinal study even reported lower plasma levels of QA and QA/KA in participants progressing to MCI or AD [49]. Another longitudinal study found no associations between plasma QA levels and risk of incident all-type dementia or AD dementia [45]. Furthermore, studies examining cognitive test scores generally report no statistically significant associations with QA levels [5,41,67,81,122,124].

It is important to note that AD-related brain lesions are microlocal and that investigating entire brain areas, as done in most studies, might lack the potential to detect subtle or regions-specific differences in QA levels. However, the findings from our meta-analysis together with previous observational studies contradict the assumption that QA levels are higher in AD and other dementias (which was previously linked to its presumed neurotoxic properties) [134,141]. These findings emphasize the need for further research to clarify the exact role of QA in AD and other neurodegenerative diseases.

### Higher central and lower peripheral levels of KA in patients with AD dementia

Results from our meta-analysis suggest that AD dementia is associated with higher CSF levels and lower blood levels of KA. When comparing individuals with MCI to controls, CSF levels of KA were higher as well, while blood levels were not significantly different.

In line with our findings, previous studies with post-mortem brain tissue reported increased levels of KA in several areas of individuals with AD [60,142] and Down syndrome (DS) [60,142], whereas other studies reported no differences [111,143]. Similarly, KA levels measured in red blood cells of patients with AD dementia [82] and in urine of patients with AD dementia and MCI were lower compared to controls [23]. Moreover, higher concentrations of KA in CSF were positively associated with tau pathology and with a slower progression of dementia [51]. These results, together with those from our meta-analyses, suggest that central levels of KA are higher in individuals with evident cognitive decline, whereas peripheral levels of KA are lower.

In contrast to QA, KA is considered a neuroprotective metabolite through its role as an antagonist of the NMDA receptor and α7 nicotinic acetylcholine (α7nACh) receptor and has antioxidant and anti-inflammatory properties [141]. In in vivo studies, experimentally increasing KA levels in the brain provided neuroprotection in rodent models of AD-like pathology [144,145] and cerebral ischemia [146,147]. This suggests that during (neuro)inflammation, astrocytes may upregulate the production of KA to mitigate neurotoxicity [148]. In contrast, peripheral tissues may experience a shift toward a more neurotoxic environment. KA exhibits anti-inflammatory properties by modulating key cytokines, including tumor necrosis factor-alpha (TNF-α), interferon-gamma (IFN-γ), and interleukin-4 (IL-4) [149]. A reduction in KA levels may, therefore, reflect a diminished capacity to protect against tissue damage resulting from excessive inflammation.

### Central and peripheral levels of TRP in patients with AD dementia

Results from our meta-analysis suggest that TRP levels are lower in CSF and blood of patients with AD dementia, consistent with results from previously published meta-analyses [27–29]. However, TRP levels were also lower in blood of individuals with MCI compared to controls. In CSF, a similar non-significant trend was found, suggesting that reduced TRP availability may occur early in the disease process. The KP is initiated by indoleamine 2,3 dioxygenase (IDO), which converts TRP to KYN and which is stimulated by inflammatory stimuli such as IFN-γ and TNF-α. Since AD is associated with inflammatory activation of IDO this may contribute, at least in part, to the lower TRP concentrations observed in MCI and AD patients. However, on top of its known role as a biochemical precursor for the kynurenine and serotonin pathways, studies have shown that TRP has antioxidant properties, such as scavenging free radicals, reactive oxygen, and chlorine species, as well and displays the highest antiradical activity compared to other amino acids [150–152]. As such, lower central and peripheral levels of TRP, as demonstrated in our meta-analyses, potentially indicates less antioxidant activities in patients with MCI or AD dementia.

### Central and peripheral levels of other KP metabolites in patients with AD dementia

Our meta-analysis found no significant differences in KYN levels in CSF or blood of patients with MCI or AD dementia compared to controls. This is in line with results from longitudinal studies investigating associations between blood levels of KYN and dementia risk [45–49] and suggests that KYN levels may remain stable or vary in a region-specific manner in the brain, which broader analyses of CSF or blood might miss. Lower CSF levels of 3-HK and AA, and lower blood levels of XA and 3-HAA were found in patients with AD dementia. For AA, there was a similar but non-significant trend in blood. In contrast, overall levels of PIC were higher in AD dementia, with similar non-significant trends for CSF and blood. With respect to KTR, we found higher levels in individuals with MCI, but generally, no differences were found in AD dementia, which may indicate that alterations in KTR are more prominent in earlier stages of cognitive decline. For XA, AA, 3-HAA, and PIC, there were not enough studies investigating differences in metabolite levels between individuals with MCI and controls to conduct separate meta-analyses for CSF and blood. For some of these metabolites, findings were surprising given their presumed biological properties, and inconsistent evidence from observational studies further complicate understanding of their role in AD and MCI. For instance, one longitudinal study found that higher AA levels were associated with an increased dementia risk, while higher KTR levels predicted all-type dementia but not AD dementia and no associations were found for XA [45]. Another longitudinal study reported higher blood levels of 3-HK and 3-HAA, and lower levels of PIC in individuals progressing to MCI or AD, while no associations were found with AA or KTR [49]. These findings highlight the need for further research to clarify the role of these and other kynurenines in MCI and AD dementia progression.

### Findings from meta-regression analyses

Several factors could explain inter-study differences in metabolite levels between groups. Results from our meta-regression showed that “year of publication” explained heterogeneity of findings for KA overall, for TRP, KYN, and KTR in blood and for KYN and 3-HK in serum. Analytical technique had an influence on the estimates for 3-HK overall and in blood. While no previous studies have specifically investigated differences in KP metabolite levels as a result of the analytical technique used, some studies have reported no statistically significant differences between HPLC and LC–MS/MS. ELISA, HLPC, and MS-based techniques are commonly used to determine KP metabolites in human tissue, with MS-based techniques generally being more accurate and sensitive compared to HPLC from a technical perspective [153,154]. Therefore, technological limitations at the time of conducting the studies may explain this heterogeneity factor, since metabolite measurements in earlier studies were likely performed with less sensitive equipment. Lastly, “sample size” in blood and serum and “sex” in blood were sources of heterogeneity for KYN. As a result, these and other kynurenines should be examined in more detail, especially since most of them have been put forward as potential biomarkers.

With respect to studies comparing patients with MCI and controls, levels of TRP, KYN, KTR, KA, AA, and 3-HAA were included in a meta-regression, but no sources of heterogeneity could be identified.

### Limitations

A main limitation is the relatively small number of included studies in our meta-analysis despite our extensive and over-inclusive search strategy. We had to exclude articles reporting the metabolite levels in median and interquartile range, unless the corresponding author was able to provide additional data. Additionally, although we performed an extensive search without filters and included a downstream snowballing approach, we may have missed potential articles. Finally, we limited publications to articles written in English and as such might have excluded other articles that would otherwise have met inclusion criteria. There were also limitations in the original studies themselves. Most studies included in this systematic review and meta-analysis did not correct for important confounders such as age. Since age is also the biggest risk factor of AD dementia, and most patients with AD dementia were older compared to controls, this might also be a reason for the differences observed in this study. As such, including age as a confounder in clinical studies is essential. Another limitation is the use of hospital controls. These controls may be cognitively healthy but may present other disorders which may influence kynurenine levels and/or the risk to develop dementia. For instance, some control individuals were from the memory clinic. Although they were categorized as cognitively healthy controls, they may in fact have SCD or psychiatric disorders. Another limitation is that TRP is only available through food intake, thus fasting and non-fasting samples may influence the KP metabolite concentrations. While one study reported that both TRP and KYN levels showed no significant difference between fasting and non-fasting serum samples, overall reproducibility was lower in non-fasting samples [155]. There were also inconsistent procedures in collecting ([non]fasted samples), measuring (analytical techniques) and analyzing (covariates of) KP metabolites. Lastly, most studies included in this review focused on AD, underscoring the need for studies investigating different patient populations as well (e.g., vascular dementia, Lewy body dementia, frontotemporal dementia, and Huntington’s disease).

### Future directions

There is a need for clinical studies that better control for confounders that (in)directly affect TRP and KP metabolites and the disorder of interest. This includes demographics (e.g., age, sex, educational level), kidney function, somatic and lifestyle factors (e.g., body mass index, alcohol, and tobacco use), and other factors (e.g., cognitive and neuropsychiatric assessments, comorbidities, timing of the (non)fasted sampling and type of anticoagulation tubes). Likewise, future studies, if possible, should investigate different samples such as plasma, serum, and CSF simultaneously to better understand the dynamics of metabolite levels. Moreover, in order to better understand the role of the KP, studies should investigate more downstream metabolites and relevant ratios in addition to TRP and KYN and correlate them with pathology markers such as same-tissue amyloid or tau levels.

Furthermore, incorporating multi-omics approaches, such as transcriptomics, proteomics, and metabolomics, can provide a more comprehensive understanding of the KP’s role in AD and other disease states. These methodologies enable the simultaneous analysis of various molecular layers, facilitating the identification of key regulatory networks and potential therapeutic targets. For instance, our group recently uncovered distinct transcriptomic and epigenomic alterations in the postmortem brains and blood of AD patients and matched controls within the TRP catabolic pathway, notably in the KP-associated genes (i.e., *TDO2*, *HAAO*, *GOT2*, *IDO2*, and *KYAT3*) [156]. Hence, future investigations should employ broader multi-omics strategies to provide a comprehensive profile of the KP.

Additionally, dysregulation of the KP extends beyond cognitive disorders, with emerging evidence suggesting system-wide alterations in KP activity across a broad range of diseases, including autoimmune diseases, cancer, and metabolic conditions [4–13]. This underscores the pathway’s role in both neurodegenerative and systemic health conditions. Therefore, investigating the KP in greater detail across a wider array of diseases is important for understanding the broader implications of KP alterations and their potential as therapeutic targets.

Finally, although cross-sectional studies are informative, in order to fully understand potential specific and common pathophysiological mechanisms, it is vital to investigate kynurenines at different time points in longitudinal studies in individuals with different levels of cognitive impairment, different patient populations and patients with other comorbidities such as affective symptomatology.

## Conclusion

Despite a large heterogeneity among clinical studies and a partial inconsistency in metabolite levels, the current review suggests that TRP and kynurenines are dysregulated in patients with dementia and cognitive impairment. Yet, findings also challenge current thinking about the roles of some metabolites, in particular of QA.

## Supplementary Information

Below is the link to the electronic supplementary material. 

ESM1 (DOCX 2.64 MB)

## Data Availability

This systematic and meta-analysis review has been registered in the international prospective register of systematic reviews (PROSPERO) under the registration number: CRD42020159274 on March 11, 2020. All systematic and meta-analytic data are presented in the article and in the supplementary materials. Additional inquiries can be requested through the corresponding author.
