# Supplementary material for: Kynurenine pathway dysregulation in cognitive impairment and dementia: a systematic review and meta-analysis
Source: GeroScience. 2025 May 8;48(3):4585–619. doi: 10.1007/s11357-025-01636-3 (PMC13356250; doi:10.1007/s11357-025-01636-3)
Supplement: Supplementary file 1 — (DOCX 2.64 MB) [file 11357_2025_1636_MOESM1_ESM.docx]

**Supplemental material**

**Tables**

**Table S1** Study details of all studies included in systematic review.

**Table S2** Differences in CSF kynurenine ratios and neopterin between cases and controls.

**Table S3** Differences in plasma and serum kynurenine ratios and neopterin between cases and controls.

**Table S4** Differences in kynurenines between cases and controls in other biological materials.

**Table S5** Effect sizes and Egger’s bias coefficients of MCI-control studies.

**Table S6** Summary of Meta-regression on studies investigating differences between patients with MCI and controls.

**Table S7** Cross-sectional associations between kynurenines and MMSE scores in cases and controls.

**Table S8** Cross-sectional associations between kynurenines and other cognitive scores in cases and controls.

**Table S9** Cross-sectional associations between kynurenine ratios or neopterin and cognitive scores in samples of patients and controls.

**Table S10** Quality assessment of data included in systematic review.

**Table S11** PRISMA 2020 Checklist

**Figures**

**Figure S1** Results of meta-analyses comparing kynurenines between cases and controls.

**Figure S2** Funnel plots of AD-Control studies, separately in CSF.

**Figure S3** Funnel plots of AD-Control studies, separately in blood.

**Figure S4** Forest plots of MCI-control studies.

**Figure S5** Funnel plots of MCI-control studies.

**Figure S6** Funnel plots of MCI-control studies, separately in CSF.

**Figure S7** Funnel plots of MCI-control studies, separately in blood.

**Forms**

**Appendix S1** Data extraction form.

**Appendix S2** Newcastle-Ottawa quality assessment scale adapted for studies with different patient populations.

**Appendix S3** Newcastle-Ottawa quality assessment scale adapted for cross-sectional studies.

**Table S1** Study details of all studies included in systematic review.

| **Study** | **Design** ^a^ | **Country** | **Biol. sample** | **Groups (n)** | **Age** | **Fem.(%)** | **Med. free patients (spec.)** | **In/ outpatients** | **Diagnostic**  **criteria** | **Measured kyns** | **Method** | **Fasting** | **Storage**  **Temp.** |
| --- | --- | --- | --- | --- | --- | --- | --- | --- | --- | --- | --- | --- | --- |
| Anderson (2021)^[1]^ | Cross | US | Serum | CTRL (74) | 36.2 ± 9.4 | 100 | n/a | n/a | n/a | TRP  KYN  KA  QA  KTR  QA/KA | HPLC-MS/MS | unk | unk |
| Aquilani (2023)^[2]^ | C-C | Italy | CSF  Plasma | AD (44)  FTD (13)  VaD (8)  MCI (10)  CTRL (15) | 72.3 ± 7.6  69.9 ± 8.9  75.5 ± 9.3  71.0 ± 7.0  73.6 ± 6.3 | 47.7  46.2  37.5  50.0  26.7 | unk | unk | MMSE  CDR  Etiological diagnosis | TRP | HPLC | Yes | -80 |
| Arai (1984)^[3]^ | C-C | Japan | Post-mortem brain tissue | AD (4)  CTRL (8) | 59.5 ± 17.7  69.6 ± 11.8 | unk | Yes *(neuroleptic treatment)* | unk | unk | TRP | Hitachi 835 high-speed amino analyser | n/a | -80 |
| Arai (1985)^[4]^ | C-C | Japan | Post-mortem brain tissue | AD (5)  CTRL (8) | 61.6 ± 16.1  69.6 ± 11.8 | unk | Yes (neuroleptics, antidepressants, opiates) | unk | unk | TRP | Hitachi 835 high-speed amino analyser | n/a | -80 |
| Atukeren (2017)^[5]^ | C-C | Turkey | Serum | AD (14)  CTRL (32) | 78.9 ± 8.0  77.3 ± 6.7 | 42.9  56.3 | Yes  *(antibiotic NSAIDS, steroids)* | unk | NINCDS ADRDA | N-f-KYN  KYN | Spectrofluorometric | Yes | -80 |
| Baker (1989)^[6]^ | C-C | UK | Post-mortem brain tissue | AD (12)  CTRL (13)  AD (24)  CTRL (21) | 80.6 ± 7.3  82.5 ± 7.1  81.0 ± 7.5  80.0 ± 7.4 | 66.67  69.23  70.83  76.19 | Yes *(neuroactive drugs)* | unk | unk | TRP | HPLC | unk | unk |
| Bakker (2021)^[7]^ | C-C | NL | Plasma | MCI (440)  CTRL (1854) | 61.2 ± 7.9  60.4 ± 8.0 | 38.4  47.2 | No | n/a | >1.5 standard deviations below general population mean in one or more cognitive domains | TRP  KYN  3-HK  KA  XA  AA  3-HAA  QA  KTR  KA/QA  Neopterin | LC-MS/MS | Yes | -80 |
| Bakker (2023)^[8]^ | C-C | NL | CSF  Plasma | Dementia all (24)  AD (18)  MCI (47)  SCD (66) | 67.2 ± 6.4  68.3 ± 6.6  65.8 ± 8.0  60.4 ± 8.6 | 50.0  44.4  34.0  22.7 | No | Outpatients | DSM IV or DSM5 | TRP  KYN  3-HK  KA  XA  AA  3-HAA  PIC  QA  KTR  KA/QA  Neopterin | LC-MS/MS | Yes | -80 |
| Bakker (2023)^[9]^ | Pros. | NL | Plasma | PSCI (127)  PSNCI (67) | 65.0 ± 10.6  66.0 ± 11.4 | 31.5  29.8 | No | Outpatients | >1.5 standard deviations below general population mean in one or more cognitive domains | TRP  KYN  3-HK  KA  XA  AA  3-HAA  PIC  QA  KTR  KA/QA  Neopterin | LC-MS/MS | Yes | -80 |
| Baran (1999)^[10]^ | C-C | Austria | CSF  Serum | AD (2)  CTRL (5) | 73.2  72.3 ± 7.8 | unk | unk | unk | unk | KYN  KA  3-HK | unk | unk | unk |
| Basun (1990)^[11]^ | C-C | Sweden | Plasma | AD (22)  CTRL (11) | 74.0 ± 9.0  79.0 ± 2.0 | 59.1  54.5 | unk | Outpatients | DSM-III | TRP | unk | Yes | -70 |
| Beal (1990)^[12]^ | C-C | US | CSF | AD (9)  CTRL (50) | 76.7 ± 7.2  43.8 ± 3.2 | unk | unk | Both | unk | KA | HPLC | unk | unk |
| Bonaccorso (1998)^[13]^ | C-C | unk | Plasma | AD (15)  CTRL (15) | 78.4 ± 10.3  75.6 ± 9.1 | 80.0  46.7 | Yes *(psychotropic)* | unk | DSM-III-R | TRP | HPLC | Yes | -75 |
| Cespedes (2022)^[14]^ | Pros. | Australia | Plasma | *At follow up:*  Progressors to MCI/ AD (166)  Non-progressors – CTRL/ MCI stable (73) | 74.9 ± 7.2  75.7 ± 5.8 | 50.6  52.1 | No | Outpatients | NINCDS-ADRDA  Winblad  Petersen | TRP  KYN  3-HK  KA  AA  3-HAA  PIC  QA  KTR  3-HK/KYN  QA/KA  PIC/QA  3  -HAA/AA | UHPLC  HPLC  GC-MS | unk | -80 |
| Chatterjee (2020)^[15]^ | Cross. | Australia | Plasma | 100 | 78.2 ± 5.5 | 68.0 | n/a | n/a | n/a | TRP  KYN | UHPLC HRAM-MS | Yes | -80 |
| Chouraki (2017)^[16]^ | Pros. | US | Plasma | *At follow up:*  Dem (93)  AD (68)  CTRL (1974) | 55.3 ± 9.5  67.8 ± 6.1  unk | 52.4  51.6  unk | unk | unk | DSM-IV  NINCDS ADRDA | TRP  KYN  KA  XA  AA  3-HAA  QA | LC-MS | Yes | -80 |
| Cogo (2021)^[17]^ | C-C | France | Serum | PSCI (13)  PSNCI (10) | 69.4 ± 17.8  64.7 ± 13.3 | 38.5  40.0 | unk | Inpatients | Test battery (MMSE, MoCA, WAIS-IV, FAB, TMT, Rey figure, Stroop) | TRP  KYN  KA  QA  KTR  QA/KA | HPLC  Mass fragmentography | n/a | n/a |
| Czech (2012)^[18]^ | C-C | Europe (Germany, France, Switzerland, Sweden) | CSF | AD (79)  CTRL (51) | 69.7  63.1 ± 7.7 | 55.7  52.9 | Yes *(anticoagulants, anti-inflammatory, anti-depressives, medication for cognitive disorders, schizophrenia and anxiety)* | unk | DSM-IV  NINCDS ADRDA | TRP  KYN | GC-MS  LC-MS/MS | unk | unk |
| de Leeuw (2017)^[19]^ | C-C | NL | Plasma | AD (127)^a^  SCD (121)^a^ | 65.1 (9.1)  62.7 (8.0) | 50  46 | No | Outpatients | NINCDS-ADRDA  NIA-AA | TRP  KYN | UPLC-MS/MS | No | -80 |
| Fakhruddin (2020)^[20]^ | C-C | Malaysia | Urine | MCI (9)  CTRL (9) | unk | unk | unk | Outpatients | unk | TRP  KYN | unk | unk | -80 |
| Fekkes (1998)^[21]^ | C-C | NL | Plasma | AD (14)  CTRL (17) | 73.6 ± 6.3  70.1 ± 1.3 | 71.4  0.0 | Yes *(neuroleptics, anticonvulsive)* | Outpatients | DSM-III-R  NINCDS ADRDA | TRP | HPLC | unk | -80 |
| Fonteh (2007)^[22]^ | C-C | US | CSF  Plasma  Urine | AD (8)  CTRL (8) | 77.9 ± 7.4  79.5 ± 5.5 | 50.0  50.0 | No | unk | NINCDS-ADRDA | TRP | LC-MS/MS | unk | -80 |
| Giil (2017)^[23]^ | C-C | UK | Plasma | AD (42)  CTRL (42) | 78.5 ± 6.3  78.6 ± 6.8 | unk | unk | unk | CamCog, CERAD | TRP  KYN  3-HK  KA  XA  AA  3-HAA  QA  KTR  Neopterin | LC-MS/MS | No | -80 |
| Gold (2011)^[24]^ | Cross. | Canada | Plasma | IS (41) | 72.3 ± 12.2 | 46.3 | No | unk | NINCDS  WHO-MONICA | TRP  KYN  KTR | HPLC | Yes | -80 |
| Gonzalez-Dominguez (2014)^[25]^ | C-C | Spain | Serum | AD (22)  CTRL (18) | 78.5 ± 5.0  70.7 ± 4.1 | 54.6  61.1 | unk | unk | NINCDS ADRDA | KYN | DI-MS | Yes | -80 |
| Gonzalez-Dominguez (2015a)^[26]^ | C-C | Spain | Serum | AD (23)  CTRL (21) | 79.2 ± 5.9  72.1 ± 5.4 | 65.2  57.1 | Yes  *(all)* | Outpatients | NINCDS ADRDA | TRP | GC-MS | Yes | -80 |
| Gonzalez-Dominguez (2015b)^[27]^ | C-C | Spain | Serum | AD (30)  CTRL (30) | 80.3 ± 5.0  73.5 ± 5.9 | 60.0  66.7 | Yes  *(all)* | Outpatients | NINCDS ADRDA | PIC | FIA-APPI-QTOF-MS | unk | -80 |
| González-Sánchez (2020)^[28]^ | C-C | Spain | CSF  Plasma | Mod AD (20)  Mild AD (41)  MCI (24)  FTD (8)  CTRL (23) | 73.3 ± 7.2  71.9 ± 8.1  72.0 ± 7.1  66.4 ± 5.2  64.7 ± 10.8 | 65.0  53.7  58.4  37.4  34.8 | Yes  *(med affecting cognition or motor function)* | unk | NIA-AA, Biomarker profile, imaging | TRP  KA  KA/TRP | ELISA | unk | -80 |
| Graham (2015)^[29]^ | C-C | Ireland | Plasma | *At follow up:*  AD (19)  MCI (16)  CTRL (37) | 77.9 ± 4.4  72.4 ± 7.3  73.1 ± 8.9 | 63.2  50.0  51.4 | unk | unk | NINCDS-ADRDA  Petersen | TRP  N-f-KYN  3-HK | LC-QTOF-MS | unk | -80 |
| Greilberger (2010)^[30]^ | C-C | Austria | Plasma | AD/MCI (16)  CTRL (15) | 63.3 ± 13.7  62.8 ± 3.6 | 56.3  73.3 | unk | unk | NINCDS ADRDA | TRP  KYN  KTR  Neopterin | RP-HPLC | Yes | -70 |
| Gulaj (2010)^[31]^ | C-C | Poland | Plasma | AD (34)  CTRL (18) | 78.8 ± 5.7  76.2 ± 7.3 | 70.6  72.2 | unk | unk | DSM-IV | TRP  KYN  3-HK  KA  AA  QA  KTR  3-HK/KYN  KA/KYN  AA/KYN  QA/3-HK | HPLC | unk | -40 |
| Hafstad Solvang (2019)^[32]^ | C-C | Norway | Serum | AD (90)  LBD (65) | 75.1 ± 7.8  75.1 ± 6.3 | 67.8  40.0 | unk | unk | NINCDS ADRDA | TRP  KYN  3-HK  KA  XA  AA  PIC  QA  KTR  KA/KYN  Neopterin | LC-MS/MS | No | -80 |
| Hafstad Solvang (2019)^[33]^ | Cross. | Norway | Plasma | 2174 | 71^b^ | 55.2 | n/a | n/a | n/a | TRP  KYN  3-HK  KA  XA  AA  3-HAA  PIC  QA  KTR  Neopterin | LC-MS/MS | No | -80 |
| Hartai (2007)^[34]^ | C-C | Hungary | Plasma  Red blood cells | AD (28)  CTRL (31) | 77.0 ± 6.3  73.0 ± 8.3 | 78.6  67.7 | Yes  *(med. Influencing dopaminergic system or KP)* | unk | DSM-IV  NINCDS ADRDA | KYN  KA | HPLC | unk | unk |
| Hebbrecht (2022)^[35]^ | Pros. | Belgium | Plasma | CTRL (29) | 42.7 ± 11.6 | 45.7 | n/a | n/a | n/a | 3-HK  KA  QA  3-HK/KA | UPLC-MS/MS | unk | -70 |
| Heyes (1992)^[36]^ | C-C | US  Canada | CSF | AD (39)  CTRL (30) | 63.8 ± 1.2  59.1 ± 14.2 | unk | Yes *(all)* | unk | unk | TRP  KYN  KA  QA  QA/KA | unk | No | unk |
| Heylen (2023)^[37]^ | C-C | Belgium  Spain | Post-mortem brain tissue | Early onset AD (23)  FTD (24)  CTRL (20) | 56.4 ± 6.4  50.6 ± 8.5  56.0 ± 8.7 | 39.1  41.7  40.0 | No | unk | CERAD  Braak  Thal  Brettschneider  Mackenzie | TRP  KYN  3-HK  KA  XA  AA  PIC  QA  KTR | SPE-LC-MS/MS | unk | -80 |
| Huang (2021)^[38]^ | Cross | China | Serum | CTRL (70) | 39.7 ± 11.8 | 47.1 | n/a | n/a | n/a | KA  QA  QA/KA | LC-MS/MS | Yes | -80 |
| Huo (2020)^[39]^ | Pros. | US | Serum | *At follow up:*  AD (85)  CTRL/MCI (436) | 86 ± 5.9  81 ± 7.3 | 82.4  77.3 | No | Outpatients | CERAD  Braak  NIA-Reagan | TRP | UPLC-MS/MS | unk | -80 |
| Ibáñez (2013)^[40]^ | C-C | Sweden | CSF | AD (21)  CTRL (21)  MCI-AD (12)  MCI-Stable (21) | 69 ± 9.6  58 ± 8.9  63 ± 9.4  60 ± 8.9 | 71  57  50  33 | unk | unk | DSM-IV  NINCDS ADRDA | TRP | RP-UHPLC-MS  HILIC  UHPLC-MS | Yes | -80 |
| Ikeuchi (2022)^[41]^ | C-C | Japan | Plasma | MCI (219)  CTRL (220) | 79.5 ± 5.7  76.3 ± 6.6 | 66.2  53.2 | No | Outpatients | WMS-R LM II  CDR | TRP | HPLC-ESI-MS | Yes | unk |
| Jacobs (2019)^[42]^ | C-C | Sweden | CSF  Plasma | AD (20)  SCD (18) | 77.9 ± 7.5  73.1 ± 7.9 | 55.0  16.7 | unk | Outpatients | unk | TRP  KYN  3-HK  KA  AA  3-HAA  PIC  QA  KTR  3-HK/KYN  3-HAA/AA  PIC/QA  QA/KA  Neopterin | UHPLC  HPLC, GC-MS | unk | -80 |
| Janssens (2020)^[43]^ | C-C | Belgium | CSF  Serum | FTD (39)  CTRL (26) | 67.4 ± 11.6  67.0 ± 8.0 | 48.7  46.2 | unk | Outpatients | n/a | TRP  KYN  3-HK  KA  XA  AA  QA  PIC  KTR  3-HK/XA | LC-MS/MS | n/a | -80 |
| Kaddurah-Daouk (2011)^[44]^ | C-C | US | CSF | AD (15)  CTRL (15) | 80.0 ± 1.1  82.0 ± 8.8 | 73.0  73.0 | unk | Outpatients | CERAD | TRP  KYN  3-HAA  TRP/KYN | LC-ECA | unk | -80 |
| Kaddurah-Daouk (2013)^[45]^ | C-C | US | CSF | AD (40)  MCI (36)  CTRL (38) | 69.0  69.9  69.5 | 75.0  52.8  66.8 | unk | Outpatients | NINCDS ADRDA | TRP  KYN  KTR | LC-ECA | unk | unk |
| Kaiser (2010)^[46]^ | C-C | Germany | CSF | AD (14)  MCI (13) | 71.6 ± 8.8  67.2 ± 7.4 | 57.1  53.9 | Yes  *(all for* ≥ *3 months)* | Outpatients | NINCDS ADRDA | TRP | HPLC | Yes | -80 |
| Kindler (2020)^[47]^ | Cross | Australia | Plasma | CTRL (81) | 31.7 | 50.6 | n/a | n/a | n/a | KTR | UHPLC  GCMS (QA) | unk | -80 |
| Klatt (2021)^[48]^ | C-C | Australia | Serum | AD (28)  CTRL (93) | AD – men  75.5^b^ ± 7.9  AD – women  73^b^ ± 8.5  CTRL – men  76^b^ ± 9.5  CTRL – women  74^b^ ± 8.6 | 71.4  47.3 | Yes (*anticonvulsant, antiparkinsonian, anticoagulant, narcotic, or immunosuppressive)* | Outpatients | NINCDS ADRDA | TRP  KYN  3-HK  3-HAA  KYN/3-HK | LC-MS | Yes | -178 |
| Knapskog (2023)^[49]^ | C-C | Norway | CSF | AD (252)  MCI (59)  CTRL (105) | 71 (66 – 75)^b^  71 (68 – 75)^b^  71 (67 – 76)^b^ | 59.1  52.5  44.8 | Yes *(Symptomatic anti-dementia drugs)* | Outpatients | NIA-AA  Biomarker profile | TRP  KYN  3-HK  KA  AA  PIC  QA  KTR | LC-MS/MS | unk | -80 |
| Küster (2017)^[50]^ | Cross. | Germany | Serum | DEM (4)  MCI (32)  SCD (11) | 71.2 ± 6.0 | 57.5 | No | Outpatients | unk | TRP  KYN  3-HK  KA  QA | LC-MS/MS | No | -80 |
| Leblhuber (1998)^[51]^ | Cross. | Austria | Serum | HD (12) | 42.4 ± 11.7 | 33.3 | Yes *(neuroleptics)* | unk | Molecular genetics, autopsy | TRP  KYN  KTR  Neopterin | HPLC  ELISA | unk | -20 |
| Li (2010)^[52]^ | C-C | China | Plasma | AD (20)  CTRL (20) | 68 ± 10  70 ± 9 | 50.0  50.0 | Yes *(all)* | Outpatients | unk | TRP | UPLC-MS | unk | -80 |
| Liang (2016)^[53]^ | C-C | China | Saliva | AD (660)  MCI (583) | 78.6 ± 5.7  78.9 ± 4.9 | 50.3  50.4 | unk | Outpatients | unk | TRP | FUPLC-MS | unk | -80 |
| Lin (2019)^[54]^ | C-C | Taiwan | Plasma | AD (15)  MCI (10)  CTRL (15) | 76.9 ± 8.0  74.6 ± 8.5  66.8 ± 6.5 | unk | unk | unk | DSM-IV  NINCDS ADRDA | KTR | LC-MS/MS | Yes | unk |
| Liu (2015)^[55]^ | C-C | China | Serum | PSCI (30)  PSNCI (30) | unk | unk | unk | Inpatients | MoCA | TRP  KYN | UHPLC-QTOF-MS | Yes | -80 |
| Liu (2023)^[56]^ | C-C | China | Serum | AD (37)  CTRL (34) | 83.1 ± 7.0  75.8 ± 7.4 | 70.3  50.0 | unk | unk | NINCDS ADRDA | TRP | HPLC-MS/MS | unk | -80 |
| Martinez (1993)^[57]^ | C-C | Spain | CSF Serum | AD (13)  VaD (13)  CTRL (15) | 68.0 ± 6.0  71.0 ± 6.0  66.0 ± 8.0 | 69.2  46.2  46.7 | Yes *(neuroleptics, antidepressants)* | unk | NINCDS ADRDA | TRP | HPLC | Yes | -40 |
| Mashige (1993)^[58]^ | C-C | Japan | CSF | AD (8)  VaD (3) | 67.3 ± 15.1  75.0 ± 7.2 | 37.5  33.3 | unk | unk | unk | TRP | HPLC | unk | -80 |
| McCann (2021)^[59]^ | Pros. | Norway | Serum | AD (89)  LBD (65) | 75.0 ± 7.8  74.6 ± 6.8 | 67.4  41.5 | No | Outpatients | NINCDS ADRDA | TRP | GC-MS/MS | Yes | -80 |
| Molina (1998)^[60]^ | C-C | Spain | CSF  Plasma | AD (37)  CTRL (32) | 70.9 ± 8.5  67.9 ± 9.2 | 54.1  53.1 | unk | unk | DSM-IV  NINCDS ADRDA | TRP | Ion-exchange chromatography | Yes | -30 |
| Mourdian (1989)^[61]^ | C-C | US | Post-mortem brain tissue  CSF | AD (22)  CTRL (21)  AD (35)  CTRL (23) | 75.0 ± 9.4  73.0 ± 9.2  64.0 ± 5.9  65.0 ± 9.6 | unk | unk | unk | NINCDS ADRDA | KTR  QA | NCI  GC-MS | Yes | -70 |
| Nho (2021)^[62]^ | Pros. | US | Serum | AD (304)  Late MCI (491)  Early MCI (271)  SCD (95)  CTRL (370) | 74.8 ± 7.8  74.0 ± 7.6  71.3 ± 7.6  72.3 ± 5.7  74.6 ± 5.8 | 45.4  39.1  45.0  57.9  51.1 | No | Outpatients | WMS-R LM II  CDR  MMSE | KYN | UPLC-MS/MS | Yes | -20/ -80 |
| Oxenkrug (2017)^[63]^ | C-C | US | Serum | AD (20)  CTRL (24) | unk | 60.0  50.0 | No | unk | MMSE | TRP  KYN  3-HK  KA  XA  AA  KTR | HPLC-MS | unk | -80 |
| Paglia (2016)^[64]^ | C-C | US | Post-mortem brain tissue | AD (21)  CTRL (19) | 82.4 ± 6.7  83.5 ± 6.4 | 57.1  36.8 | unk | unk | NIA-Reagan | TRP | UPLC-HILIC-MS | n/a | unk |
| Park (2020)^[65]^ | Cross | South Korea | Serum | CTRL (40) | 73.9 ± 5.1 | 65.0 | n/a | n/a | n/a | TRP  KYN  AA | GC-TOF-MS | Yes | -80 |
| Parker (2023)^[66]^ | Pros. | US | Plasma | CTRL (301) | 74.8 ± 8.7 | 55.1 | n/a | n/a | n/a | TRP  KYN  KTR  KA/KYN | LC-MS/MS | No | unk |
| Peña-Bautista (2020)^[67]^ | C-C | Spain | Plasma | MCI due to AD (25)  CTRL (25) | 70 (67-73)^a^  66 (62-70)^a^ | 60.0  36.0 | unk | Outpatients | NIA-AA, Biomarker profile | TRP | UPLC-MS/MS | unk | -80 |
| Platzer (2017)^[68]^ | Cross | Austria | Serum | CTRL  Men (36)  Women (57) | 38.1 ± 15.1  39.4 ± 16.9 | 0.0  100 | n/a | n/a | n/a | 3-HK/KA  KYN/3-HK  KYN/KA | LC-MS/MS | Yes | unk |
| Ramos-Chavez (2018)^[69]^ | C-C | Mexico | Serum | MCI (23)  CTRL (54) | unk | unk | Yes *(immunosuppressive and immunomodulatory med)* | Outpatients | Test battery | TRP  KYN  3-HK  KA  KTR  3-HK/TRP  KA/TRP | HPLC/ fluorometric analyser | unk | -70 |
| Rommer (2016)^[70]^ | C-C | Austria | Plasma | AD/MCI (16)  CTRL (15) | 63.3 ± 13.7  62.8 ± 3.6 | 56.3  73.3 | unk | unk | NINCDS ADRDA | TRP  KYM  KTR  Neopterin | RP-HPLC | Yes | -70 |
| Rudman (1989)^[71]^ | C-C | US | Plasma | Dem (17)  CTRL (21) | 73.0  75.0 | 0.0  0.0 | unk | Inpatients | unk | TRP | HPLC | Yes | -20 |
| Santos (2020)^[72]^ | C-C | Brazil | Plasma | FTD (9)  CTRL (15) | 65.5 ± 9.5  67.7 ± 8.4 | 33.3  66.7 | Yes *(anticoagulants, anti-inflammatory)* | Outpatients | NIA-AA  Biomarker profile | TRP | GC-MS | Yes | -80 |
| Schwarz (2013)^[73]^ | C-C | Germany | Serum | AD (20)  SCD (19) | 74.0 ± 7.6  59.5 ± 10.2 | 80.0  42.1 | Yes  *(anti-inflammatory)* | Outpatients | NINCDS ADRDA | TRP  KYN  3-HK  KA  PIC  QA  3-HK/TRP  PIC/TRP | HPLC  GC-MS | unk | -80 |
| Shao (2020)^[74]^ | C-C | China | Plasma | AD (30)  MCI (13)  CTRL (43) | 71.6 ± 8.8  67.9 ± 7.2  65.5 ± 7.9 | 66.7  38.5  41.9 | unk | unk | NINCDS ADRDA  Petersen | TRP | UPLC | Yes | -80 |
| Shaw (1981)^[75]^ | C-C | UK | Plasma | Dem (32)  CTRL (70) | 77.1  70.1 | unk | unk | Inpatients | Hare (1978) scale | TRP | unk | Yes | unk |
| Sorgdrager (2019)^[76]^ | C-C | Belgium | CSF  Serum | AD (33)  CTRL (39) | 73.7 ± 6.0  71.3 ± 10.7 | 54.5  53.8 | No | Outpatients | NINCDS ADRDA | TRP  KYN  3-HK  KA  XA  QA  KTR  KA/QA  XA/3-HK | LC-MS/MS | unk | -80 |
| Storga (1996)^[77]^ | C-C | Austria | Post-mortem brain tissue | AD (8)  CTRL (6) | 61.8 ± 12.9  69.8 ± 5.4 | 37.5  50.0 | No | unk | unk | TRP | HPLC | n/a | -70 |
| Tarbit (1980)^[78]^ | C-C | UK | Post-mortem brain tissue | AD (8)  CTRL (7) | 75 ± 22.6  78 ± 29.1 | unk | unk | Inpatients | unk | TRP | Rank-Hilger  Chromospek amino acid analyser | n/a | -20 |
| Teruya (2021)^[79]^ | C-C | Japan | Plasma | Dem (8)  CTRL (8) | 84.6 ± 4.3  74.4 ± 4.5 | 50.0  50.0 | No | Inpatients | DSM-IV | TRP  KYN  QA | LC-MS | Yes | unk |
| Thomas (1986)^[80]^ | C-C | UK | Plasma | Dem (23)  CTRL (23) | 77.2  76.1 | 60.9  60.9 | Yes  *(meds interfering with vitamin metabolism or intestinal absorption)* | Inpatients | History, clinical characteristics and test performance | TRP | Ultra-filtration method | Yes | -20 |
| Tohgi (1992)^[81]^ | C-C | Japan | CSF | AD (14)  CTRL (10) | 68.4 ± 10.1  68.5 ± 6.1 | unk | unk | unk | DSM III-R  Hachinski  NINCDS ADRDA  CT/ MRI | TRP  KYN  3-HK  KTR  3-HK/TRP | HPLC | Yes | -80 |
| Tohgi (1995)^[82]^ | C-C | Japan | CSF | AD (15)  CTRL (10) | 68.0 ± 6.0  68.5 ± 6.1 | unk | unk | unk | DSM III-R  Hachinski  NINCDS ADRDA  CT/ MRI | TRP  KYN  3-HK  KYN/3-HK | HPLC | Yes | -80 |
| Toledo (2017)^[83]^ | Pros. | US  Canada | Serum | *At baseline:*  AD (175)  MCI (356)  CTRL (199) | 75.6  75.1  75.3 | 51.4  64.6  50.3 | No | Outpatients | NINCDS ADRDA  PET/ MRI | TRP  KYN | UPLC-MS/MS | Yes | -20/ -80 |
| Trushina (2013)^[84]^ | C-C | US | CSF Plasma | AD (15)  MCI (15)  CTRL (15) | 82.7 ± 4.2  80.4 ± 4.2  78.6 ± 3.5 | 20.0  27.0  33.3 | No | Outpatients | DSM-IV  NINCDS ADRDA | TRP | LC-MS | Yes | -80 |
| Tsuruoka (2013)^[85]^ | C-C | US | Serum  Saliva | AD (3)  FTD (4)  LBD (3)  CTRL (9) | 64.3 ± 16.9  72.0 ± 2.9  75.3 ± 4.9  68.1 ± 13.7 | 0.0  0.0  33.3  100 | unk | unk | NINCDS ADRDA | TRP | CE-TOF-MS | unk | -80 |
| Van der Velpen (2019)^[86]^ | C-C | Switzerland | CSF  Plasma | AD (40)  CTRL (34) | 74.9 ± 6.4  65.4 ± 6.2 | 60.0  67.7 | unk | Outpatients | CDR  Biomarker profile, CT/ MRI | TRP  KYN  3-HK  KA  AA  QA | UHPLC-MS | unk | unk |
| Vints (2022)^[87]^ | Cross | Lithuania | Serum | CTRL (74) | 69.4 ± 6.2 | 54.1 | n/a | n/a | n/a | KYN | ELISA | unk | -80 |
| Watkins (1989)^[88]^ | C-C | UK | Plasma | AD (22)  CTRL (22) | 77.3  76.0 | 68.2  68.2 | Yes  *(meds interfering with internal absorption)* | Inpatients | History, clinical characteristics and test performance | TRP | unk | Yes | -20 |
| Wennström (2014)^[89]^ | C-C | Sweden | CSF | AD (19)  LBD (18)  CTRL (20) | 75.0  77.0  76.0 | 52.6  55.6  50.0 | No | Outpatients | DSM-IV  NINCDS ADRDA | KA | RP-HPLC | unk | -80 |
| Whiley (2021)^[90]^ | C-C | Europe | Serum  Urine | AD (103)  MCI (165)  CTRL (86) | 76.5 ± 6.0  76.3 ± 6.0  75.9 ± 5.2 | 51.5  57.0  48.8 | No | Outpatients | DSM-IV  NINCDS ADRDA | TRP  KYN  3-HK  KA  XA  3-HAA  QA  PIC  KTR | UHPLC-MS/MS  UHPLC-QTOF-MS | unk | -80 |
| Widner (1999)^[91]^ | C-C | Austria | Serum | AD (24)  CTRL (unk) | unk | unk | Yes *(neuroleptics)* | unk | unk | TRP  KYN  KTR  Neopterin | HPLC  ELISA | unk | -20 |
| Widner (2000)^[92]^ | C-C | Austria | Serum | AD (21)  CTRL (20) | 74.4 ± 5.4  73.4 ± 7.4 | 71.4  50.0 | Yes *(nootropics)* | Outpatients | NINCDS ADRDA | TRP  KYN  KTR | HPLC | unk | unk |
| Willette (2021)^[93]^ | C-C | US  Canada | Serum | AD (112)  MCI (396)  CTRL (58) | 74.8 ± 8.1  74.7 ± 7.4  75.1 ± 5.8 | 42.0  35.4  48.3 | Yes  (*SSRIs, cholinesterase inhibitors, NMDA antagonists)* | Outpatients | NINCDS ADRDA | TRP  KYN  KTR | LC-MS | unk | unk |
| Wissmann (2013)^[94]^ | Cross. | Austria | Serum | AD (43) | 81.7 ± 10.5 | 60.5 | No | unk | NINCDS ADRDA  PET/ MRI | KTR  Neopterin | RP-HPLC  ELISA | unk | unk |
| Wu (2021)^[95]^ | C-C | China | Faeces | AD (27)  MCI (22)  CTRL (28) | 74.2 ± 11.2  70.0 ± 11.3  74.3 ± 9.0 | 44.4  59.1  50.0 | unk | Outpatients | DSM-IV  NINCDS ADRDA | KYN  KA | UPLC-MS | Yes | unk |
| Xu (2016)^[96]^ | C-C | New Zealand | Post-mortem brain tissue | AD (9)  CTRL (9) | 70.3 ± 7.1  70.1 ± 6.7 | 44.4  44.4 | unk | unk | CERAD  Braak | TRP | GC-MS | unk | -80 |
| Xu (2021)^[97]^ | C-C | Europe | Plasma | AD (137)  MCI (275)  CTRL (283) | 70 ± 8.5  70 ± 8.1  65 ± 7.9 | 59.1  51.3  54.8 | No | Outpatients | NINCDS ADRDA | KA  XA | UPLC-MS/MS | Yes | unk |
| Yilmaz (2020)^[98]^ | C-C | US | Urine | AD (20)  MCI (10)  CTRL (29) | 79.9 ± 9.1  76.6 ± 9.4  79.1 ± 6.3 | 55.0  50.0  55.2 | unk | Outpatients | NINCDS ADRDA | TRP | UPLC | Yes | -80 |

unk unknown, n/a not applicable. ^a^Indicates the design of the data included in this systematic review and does not necessarily correspond to the design of the original study to which these data belong. ^b^Age in median (IQR). Abbreviations: C-C Case-control, Cross. Cross-sectional, Pros. Prospective, AD Alzheimer’s dementia, CTRL Controls, Dem Dementia, IS Ischemic stroke patients, MCI Mild Cognitive Impairment, HD Huntington’s disease, PSCI Post-stroke cognitive impairment, PSNCI Post-stroke no cognitive impairment, SCD Subjective Cognitive Decline, LBD Lewy Body Dementia, Cent. Centenarians, NINCDS ADRDA National Institute of Neurological and Communicative Disorders and Stroke-Alzheimer’s Disease and Related Disorders Association, NIA-AA National Institute on Aging – Alzheimer’s Association, WMS-R LM Wechsler Memory Scale-Revised Logical Memory, CDR Clinical Dementia Rating, CERAD Consortium to Establish a Registry for Alzheimer’s Disease, WHO-MONICA WHO Multinational Monitoring of Trends and Determinants in Cardiovascular Disease, FAB Frontal Assessment Battery, MMSE Mini Mental State Examination, CamCog Cambridge Cognition examination, MoCA Montreal Cognitive Assessment, WAIS Wechsler Adult Intelligence Scale, ELISA Enzyme-linked immunosorbent assay, GC Gas chromatography, LC Liquid chromatography, HPLC High-performance liquid chromatography, UPLC/ UHPLC Ultra high performance liquid chromatography, MS mass spectrometry, MS/MS tandem mass spectrometry, HPLC-ESI-MS High-performance liquid chromatography electrospray ionization mass spectrometry, UHPLC-ESI-TQ-MS Ultra high-performance liquid chromatography electrospray ionization-triple quadrupole-mass spectrometry, QTOF Quadrupole time of flight, HILIC Hydrophilic interaction liquid chromatography, NCI Negative chemical ionization, FIA Flow injection analysis, ECA Electrochemical array, HRAM High resolution accurate mass, APPI Atmospheric pressure photo ionization.

**Table S2** Differences in CSF kynurenine ratios and neopterin between cases and controls.

| **Study** | **Cases** | | | **Controls** | | | **Cov. in analyses** | **Ratios** | | | | | | | | | | **Neop** |
| --- | --- | --- | --- | --- | --- | --- | --- | --- | --- | --- | --- | --- | --- | --- | --- | --- | --- | --- |
|  | **N** | **age** | **% female** | **N** | **age** | **% female** |  | **TRP/**  **KYN** | **HK/**  **TRP** | **KYN/**  **HK** | **PIC/**  **QA** | **QA/**  **KA** | **KA/QA** | **KA/**  **TRP** | **HAA/**  **AA** | **HK/XA** | **XA/HK** |  |
| ***Alzheimer’s dementia*** | | | | | | | | | | | | | | | | | | |
| González-Sánchez (2020)^[28]^ | Mod (20) | 73.3 ± 7.2 | 65.0 | 23 | 64.7 ± 10.8 | 34.8 | None | - | - | - | - | - | - | ns | - | - | - | - |
|  | Mild (41) | 71.9 ± 8.1 | 53.7 |  |  |  |  | - | - | - | - | - | - | ↑ | - | - | - | - |
| Jacobs (2019)^[42]^ | 20 | 77.9 ± 7.5 | 55.0 | 18 | 73.1 ± 7.9 | 16.7 | Sex | - | - | - | ­ ns | ↓ | - | - | ns | - | - | ns |
| Sorgdrager (2019)^[76]^ | 33 | 73.7 ± 6.0 | 54.5 | 39 | 71.3 ± 10.7 | 53.8 | Age, sex | - | - | - | - | - | ↓ | - | - | - | ns | - |
| Kaddurah-Daouk (2011)^[44]^ | 15 | 80.0 ± 1.1 | 73.0 | 15 | 82.0 ± 8.8 | 73.0 | clinical/ demographic measures, compound ratios | ns | - | - | - | - | - | - | - | - | - | - |
| Tohgi (1995)^[82]^ | 15 | 68.0 ± 6.0 | unk | 10 | 68.5 ± 6.1 | unk | None | - | - | ↑ | - | - | - | - | - | - | - | - |
| Heyes (1992)^[36]^ | 39 | 63.8 ± 1.2 | unk | 30 | 59.1 ± 14.2 | unk | None | - | - | - | - | ns | - | - | - | - | - | - |
| Tohgi (1992)^[81]^ | 14 | 68.4 ± 10.1 | unk | 10 | 68.5 ± 6.1 | unk | None | - | ↓ | - | - | - | - | - | - | - | - | - |
| ***Frontotemporal dementia*** | | | | | | | | | | | | | | | | | | |
| González-Sánchez (2020)^[28]^ | 8 | 66.4 ± 5.2 | 37.4 | 23 | 64.7 ± 10.8 | 34.8 | None | - | - | - | - | - | - | ns | - | - | - | - |
| Janssens (2020) ^[43]^ | 39 | 67.4 ± 11.6 | 48.7 | 25 | 67.3 ± 8.1 | 37.9 | None | - | - | - | - | - | - | - | - | ns | - | - |
| ***All type dementia*** | | | | | | | | | | | | | | | | | | |
| Bakker (2023)^[8]^ | 24 | 67.2 ± 6.4 | 50.0 | SCD (66) | 60.4 ± 8.6 | 22.7 | Age, sex, educational level. eGFR | - | - | - | - | - | ns | - | - | - | - | ns |
| ***Mild cognitive impairment*** | | | | | | | | | | | | | | | | | | |
| Bakker (2023)^[8]^ | 47 | 65.8 ± 8.0 | 34.0 | SCD (66) | 60.4 ± 8.6 | 22.7 | Age, sex, educational level, eGFR | - | - | - | - | - | ns | - | - | - | - | ns |
| González-Sánchez (2020)^[28]^ | 24 | 72.0 ± 7.1 | 58.4 | 23 | 64.7 ± 10.8 | 34.8 | None | - | - | - | - | - | - | ns | - | - | - | - |

↑ higher in cases, ↓ lower in cases, *ns* non-significant, *unk* unknown, - metabolite not measured. If not stated otherwise, controls consisted of neurologically healthy individuals. Abbreviations: *Mod* Moderate, *TRP* Tryptophan, *KYN* Kynurenine, *KA* Kynurenic acid, *AA* Anthranilic acid, *HK* 3-Hydroxy-Kynurenine, *HAA* 3-Hydroxyanthranilic acid, *XA* Xanthurenic acid, *QA* Quinolinic acid, *PIC* Picolinic acid, *Neop* Neopterin.

**Table S3** Differences in plasma and serum kynurenine ratios and neopterin between cases and controls.

| **Study** | **Cases** | | | **Controls** | | | **Tissue** | **Cov.**  **in analyses** |  | **Ratios** | | | | | | | | | | | | | **Neop** |
| --- | --- | --- | --- | --- | --- | --- | --- | --- | --- | --- | --- | --- | --- | --- | --- | --- | --- | --- | --- | --- | --- | --- | --- |
|  | **N** | **age** | **%**  **female** | **N** | **age** | **%**  **female** |  |  | **KYN/HK** | **HK/**  **KYN** | **AA/**  **KYN** | **HAA/AA** | **PIC/**  **QA** | **QA/**  **KA** | **KA/QA** | **KA/ KYN** | **XA/HK** | **QA/HK** | **KA/**  **TRP** | **HK/**  **TRP** | **PIC/**  **TRP** | **HK/**  **XA** |  |
| ***Alzheimer’s dementia*** | | | | | | | | | | | | | | | | | | | | | | | |
| Klatt (2021)^[48]^ | 28 | Men  75.5^a^ ± 7.9  Women  73^a^ ± 8.5 | 71.4 | 93 | Men  76^a^ ± 9.5  Women  74^a^ ± 8.6 | 47.3 | S | Age, sex | ns | - | - | - | - | - | - | - | - | - | - | - | - | - | - |
| Sorgdrager (2019)^[76]^ | 33 | 73.7  ± 6.0 | 54.5 | 39 | 71.3  ± 10.7 | 53.8 | S | Age, sex | - | - | - | - | - | - | ns | - | ↓ | - | - | - | - | - | - |
| Schwarz (2013)^[73]^ | 20 | 74  ± 7.6 | 80.0 | SCD  (19) | 59.5  ± 10.2 | 42.1 | S | Age, sex | - | - | - | - | - | - | - | - | - | - | - | ↑ | ns | - | - |
| Widner (1999)^[91]^ | 24 | unk | unk | unk | unk | unk | S | None | - | - | - | - | - | - | - | - | - | - | - | - | - | - | ↑ |
| González-Sánchez (2020)^[28]^ | Mod  (20) | 73.3  ± 7.2 | 65.0 | 23 | 64.7  ± 10.8 | 34.8 | P | None | - | - | - | - | - | - | - | - | - | - | ns | - | - | - | - |
|  | Mild  (41) | 71.9  ± 8.1 | 53.7 |  |  |  |  |  | - | - | - | - | - | - | - | - | - | - | ns | - | - | - | - |
| Jacobs (2019)^[42]^ | 20) | 77.9  ± 7.5 | 55.0 | 18 | 73.1  ± 7.9 | 16.7 | P | Sex | - | ns | - | ns | ns | ns | - | - | - | - | - | - | - | - | ns |
| Giil (2017)^[23]^ | 42 | 78.5  ± 6.3 | unk | 42 | 78.6  ± 6.8 | unk | P | Age, sex, creatinine | - | - | - | - | - | - | - | - | - | - | - | - | - | - | ns |
| Gulaj (2010)^[31]^ | 34 | 78.8  ± 5.7 | 70.6 | 18 | 76.17  ± 7.3 | 72.2 | P | None | - | ↓ | ↓ | - | ­- | - | - | ↓ | - | ↑ | ­- | - | ­- | - | - |
| ***Frontotemporal dementia*** | | | | | | | | | | | | | | | | | | | | | | | |
| Janssens (2020) ^[43]^ | 39 | 67.4  ± 11.6 | 48.7 | 26 | 67.0  ± 8.0 | 46.2 | S | None | - | - | - | - | - | - | - | - | - | - | - | - | - | ns | - |
| ***All type dementia*** | | | | | | | | | | | | | | | | | | | | | | | |
| Bakker (2023)^[8]^ | 24 | 67.2 ± 6.4 | 50.0 | SCD (66) | 60.4 ± 8.6 | 22.7 | P | Age, sex, educational level. eGFR | - | - | - | - | - | - | ns | - | - | - | - | - | - | - | ns |
| ***Mild cognitive impairment + Alzheimer’s dementia*** | | | | | | | | | | | | | | | | | | | | | | | |
| Rommer (2016)^[70]^ | 16 | 63.3  ± 13.7 | 56.3 | 15 | 62.8  ± 3.6 | 73.3 | P | None | - | - | - | - | - | - | - | - | - | - | - | - | - | - | ns |
| Greilberger (2010)^[30]^ | 16 | 63.3  ± 13.7 | 56.3 | 15 | 62.8  ± 3.6 | 73.3 | P | None | - | - | - | - | - | - | - | - | - | - | - | - | - | - | ns |
| ***Mild cognitive impairment*** | | | | | | | | | | | | | | | | | | | | | | | |
| Ramos-Chavez (2018)^[69]^ | 23 | unk | unk | 54 | unk | unk | S | Age, TRP | - | - | - | - | - | - | - | - | - | - | ↑ | ↑ | - | - | - |
| Bakker (2023)^[8]^ | 47 | 65.8 ± 8.0 | 34.0 | SCD (66) | 60.4 ± 8.6 | 22.7 | P | Age, sex, educational level. eGFR | - | - | - | - | - | - | ns | - | - | - | - | - | - | - | ns |
| Bakker (2021)^[7]^ | 440 | 61.2 ± 7.9 | 38.4 | 1854 | 60.4 ± 8.0 | 47.2 | P | None | - | - | - | - | - | - | ns | - | - | - | - | - | - | - | ↑ |
| González-Sánchez (2020)^[28]^ | 24 | 72.0  ± 7.1 | 58.4 | 23 | 64.7  ± 10.8 | 34.8 | P | None | - | - | - | - | - | - | - | - | - | - | ns | - | - | - | - |
| ***Post-stroke cognitive impairment*** | | | | | | | | | | | | | | | | | | | | | | | |
| Cogo (2021)^[17]^ | 13 | 69.4  ± 17.8 | 38.5 | PSNCI  (10) | 64.7  ± 13.3 | 40.0 | S | None | - | - | - | - | - | ↑ | - | - | - | - | - | - | - | - | - |
| Bakker (2023)^[9]^ | 127 | 65.0 ± 10.6 | 31.5 | PSNCI (67) | 66.0 ± 11.4 | 29.8 | P | None | - | - | - | - | - | - | ns | - | - | - | - | - | - | - | ns |

↑ higher in cases, ↓ lower in cases, *ns* non-significant, *unk* unknown, - metabolite not measured. If not stated otherwise, controls consisted of neurologically healthy individuals. Abbreviations: *SCD* Subjective cognitive Decline, *PSCNI* Post-stroke no cognitive impairment, *S* Serum, *P* Plasma, *TRP* Tryptophan, *KYN* Kynurenine, *KA* Kynurenic acid, *AA* Anthranilic acid, *HK* 3-Hydroxy-Kynurenine, *HAA* 3-Hydroxyanthranilic acid, *XA* Xanthurenic acid, *QA* Quinolinic acid, *PIC* Picolinic acid, *KTR* Kynurenine-Tryptophan ratio, *Neop* Neopterin.

**Table S4** Differences in kynurenines between cases and controls in other biological materials.

| **Study** | **Cases** | | | **Controls** | | | **Cov. in analyses** | **Metabolites** | | | | | | | | | |
| --- | --- | --- | --- | --- | --- | --- | --- | --- | --- | --- | --- | --- | --- | --- | --- | --- | --- |
|  | **N** | **age** | **%**  **female** | **N** | **age** | **%**  **female** |  | **TRP** | **KYN** | **3-HK** | **KA** | **XA** | **AA** | **3-HAA** | **QA** | **PIC** | **KTR** |
| ***Red blood cells*** | | | | | | | | | | | | | | | | | |
| Hartai (2007)^[34]^ | AD (28) | 77.0 ± 6.3 | 78.6 | 31 | 73.0 ± 8.3 | 67.7 | None | - | ns | - | ↓ | - | - | - | - | - | - |
| ***Saliva*** | | | | | | | | | | | | | | | | | |
| Liang (2016)^[53]^ | AD (660) | 78.6 ± 5.7 | 50.3 | MCI (583) | 78.9 ± 4.9 | 50.4 | None | ↑ | - | - | - | - | - | - | - | - | - |
| Tsuruoka (2013)^[85]^ | AD (3) | 64.3 ± 16.9 | 0.0 | 9 | 68.1 ± 13.7 | 100 | None | ns | - | - | - | - | - | - | - | - | - |
|  | FTD (4) | 72.0 ± 2.9 | 0.0 |  |  |  |  | ns | - | - | - | - | - | - | - | - | - |
|  | LBD (3) | 75.3 ± 4.9 | 33.3 |  |  |  |  | ns | - | - | - | - | - | - | - | - | - |
| ***Urine*** | | | | | | | | | | | | | | | | | |
| Whiley (2021)^[90]^ | AD (103) | 76.5 ± 6.0 | 51.5 | 86 | 75.9 ± 5.2 | 48.8 | None | ↓ | ns | ns | ↓ | ↓ | - | ns | - | - | ↓ |
|  | MCI (165) | 76.3 ± 6.0 | 57.0 |  |  |  |  | ns | ns | ns | ↓ | ↓ | - | ns | - | - | ↓ |
| Fakhruddin (2020)^[20]^ | MCI (9) | unk | unk | 9 | unk | unk | None | ↓ | ↓ | - | - | - | - | - | - | - | - |
| Yilmaz (2020)^[98]^ | AD (20) | 79.9 ± 9.1 | 55.0 | 29 | 79.1 ± 6.3 | 55.2 | None | ns | - | - | - | - | - | - | - | - | - |
|  | MCI (10) | 76.6 ± 9.4 | 50.0 |  |  |  |  | ns | - | - | - | - | - | - | - | - | - |
| Fonteh (2007)^[22]^ | AD (8) | 77.9 ± 7.4 | 50.0 | 8 | 79.5 ± 5.5 | 50.0 | None | ns | - | - | - | - | - | - | - | - | - |
| ***Faeces*** | | | | | | | | | | | | | | | | | |
| Wu (2021)^[95]^ | AD (27) | 74.2 ± 11.2 | 44.4 | 28 | 74.3 ± 9.0 | 50.0 | None | - | ns | - | ns | - | - | - | - | - | - |
|  | MCI (22) | 70.0 ± 11.3 | 59.1 |  |  |  |  | - | ns | - | ns | - | - | - | - | - | - |
| ***Post-mortem brain tissue*** | | | | | | | | | | | | | | | | | |
| Heylen (2023)^[37]^ | AD early onset (23)  Premotor/ SMA (A6)  Medial/ prefrontal (A10)  PST (A22)  Substantia nigra  Hippocampus  Neostriatum | 56.4 ± 6.4 | 39.1 | 20  Premotor/ SMA (A6)  Medial/ prefrontal (A10)  PST (A22)  Substantia nigra  Hippocampus  Neostriatum | 56.0 ± 8.7 | 40.0 | None | ns ns  ns  ns  ns  ns | ns  ns  ns  ns  ns  ns | ns  ↓  ↓  ns  ns  ns | ns  ns  ns  ns  ns  ns | ns  ns  ns  ns  ns  ns | ↑  ↑  ns  ↑  ns  ns | - | ns  ns  ns  ns  ns  ns | ns  ns  ns  ns  ns  ns | ns  ns  ns  ns  ns  ns |
|  | FTD (23)  Premotor/ SMA (A6)  Medial/ prefrontal (A10)  PST (A22)  Substantia nigra  Hippocampus  Neostriatum | 50.6 ± 8.5 | 41.7 |  |  |  |  | ns ns  ns  ns  ns  ns | ns  ns  ns  ns  ns  ns | ns  ns  ns  ns  ns  ns | ns  ns  ns  ns  ns  ns | ns  ns  ns  ns  ns  ns | ns  ns  ns  ns  ns  ns | - | ns  ns  ns  ns  ns  ns | ns  ns  ns  ns  ns  ns | ns  ns  ns  ns  ns  ns |
| Paglia (2016)^[64]^ | AD (21)  Frontal cortex | 82.4 ± 6.7 | 57.1 | 19  Frontal cortex | 83.5 ± 6.4 | 36.8 | None | ns | - | - | - | - | - | - | - | - | - |
| Xu (2016)^[96]^ | AD (9)  Hippocampus  Entorhinal cortex  Middle temporal gyrus  Sensory cortex  Motor cortex  Cingulate gyrus  Cerebellum | 70.3 ± 7.1 | 44.4 | 9  Hippocampus  Entorhinal cortex  Middle temporal gyrus  Sensory cortex  Motor cortex  Cingulate gyrus  Cerebellum | 70.1 ± 6.7 | 44.4 | None | ↑  ↑  ↑  ns  ↑  ↑  ns | - | - | - | - | - | - | - | - | - |
| Storga (1996)^[77]^ | AD (8)  Globus pallidus  Putamen  Nucleus amygdalae  Nucleus caudatus  Substantia nigra  Gyrus cingula  Raphe | 61.8 ± 12.9 | 37.5 | 6  Globus pallidus  Putamen  Nucleus amygdalae  Nucleus caudatus  Substantia nigra  Gyrus cingula  Raphe | 69.8 ± 5.4 | 50.0 | None | ns  ns  ns  ns  ns  ns  ns | - | - | - | - | - | - | - | - | - |
| Baker (1989)^[6]^ | AD  Hippocampus (n=12)  Substantia innominata (n=24) | 80.6 ± 7.3  81.0 ± 7.5 | 66.67  70.83 | Hippocampus (n=13)  Substantia innominata (n=21) | 82.5 ± 7.1  80.0 ± 7.4 | 69.23  76.19 | None | ns  ns | - | - | - | - | - | - | - | - | - |
| Mourdian (1989)^[61]^ | AD  Frontal (A4) (n=12)  Frontal (A9) (n=10)  Parietal (A39) (n=4)  Temporal (A22) (n=9)  Temporal (A38) (n=11)  Occipital (A17) (n=11)  Hippocampus (n=9)  Caudate (n=18) | 75.0 ± 9.4 | unk | Frontal (A4) (n=6)  Frontal (A9) (n=11)  n=  Temporal (A22) (n=14)  Temporal (A38) (n=10)  Occipital (A17) (n=16)  Hippocampus (n=11)  Caudate (n=10) | 73.0 ± 9.2 | unk | None | - | - | - | - | - | - | - | ns  ns  ns  ns  ns  ns  ns  ns | - | - |
| Arai (1985)^[4]^ | AD (5)  Superior frontal  Orbital  Cingulate  Inferior temporal (n=4)  Insular | 61.6 ± 16.1 | unk | 5  Superior frontal  Orbital  Cingulate  Inferior temporal (n=4)  Insular | 69.6 ± 11.8 | unk | None | ns  ns  ns  ns  ns | - | - | - | - | - | - | - | - | - |
| Arai (1984)^[3]^ | AD (4)  Temporal | 59.5 ± 17.7 | unk | 8  Temporal | 69.6 ± 11.8 | unk | None | ns | - | - | - | - | - | - | - | - | - |
| Tarbit (1980)^[78]^ | AD (8)  Hippocampus | 75 ± 22.6 | unk | 7  Hippocampus | 78 ± 29.1 | unk | None | ns | - | - | - | - | - | - | - | - | - |

↑ Sig. higher in cases, ↓ Sig. lower in cases, *ns* non-significant, *unk* unknown, - metabolite not measured. If not stated otherwise, controls consisted of neurologically healthy individuals. Abbreviations: *AD* Alzheimer’s dementia, *FTD* Frontotemporal dementia, *LBD* , Lewy body dementia, *MCI* mild cognitive impairment, *Dem* dementia, *CTRL* controls, *PSCI* post-stroke cognitive impairment, *PSNCI* post-stroke no cognitive impairment, *P* plasma, *S* Serum, *SMA* supplementary motor area, *PST* posterior superior temporal cortex, *med* medication, *supp* supplements, *TRP* tryptophan, *KYN* kynurenine, *KA* kynurenic acid, *AA* anthranilic acid, *3-HK* 3-hydroxykynurenine, *3-HAA* 3-hydroxyanthranilic acid, *XA* xanthurenic acid, *QA* quinolinic acid, *PIC* picolinic acid, *KTR* kynurenine-tryptophan ratio, *Neop* neopterin.

**Table S5** Effect sizes and Egger’s bias coefficients of MCI-control studies.

|  | | **Effect size** | | **Heterogeneity** | | **Publication bias** | |
| --- | --- | --- | --- | --- | --- | --- | --- |
|  | **N** | **SMD (95% CI)^a^** | ***p*-value** | ***I^2^* (%)** | ***p*-value** | **Egger’s bias coefficient** | ***p*-value** |
| **Tryptophan** | | | | | | | |
| Overall | 12 | -0.13 (-0.21, -0.06) | **0.001** | 57.8 | **0.006** | -0.57 | 0.463 |
| CSF | 4 | -0.20 (-0.41, 0.02) | 0.076 | 72.1 | **0.013** | -1.58 | 0.680 |
| Blood | 8 | -0.12 (-0.21, -0.04) | **0.003** | 53.1 | **0.037** | -0.40 | 0.668 |
| Plasma | 6 | -0.12 (-0.21, -0.03) | **0.006** | 40.9 | 0.133 | 0.01 | 0.995 |
| Serum | 2 | - | - | - | - | - | - |
| **Kynurenine** | | | | | | | |
| Overall | 7 | 0.02 (-0.07, 0.11) | 0.647 | 55.6 | **0.035** | 0.23 | 0.846 |
| CSF | 3 | 0.04 (-0.18, 0.25) | 0.724 | 75.6 | **0.016** | 10.87 | **0.095** |
| Blood | 4 | 0.02 (-0.08, 0.11) | 0.729 | 43.1 | 0.153 | -0.33 | 0.839 |
| Plasma | 2 | - | - | - | - | - | - |
| Serum | 2 | - | - | - | - | - | - |
| **KTR** | | | | | | | |
| Overall | 8 | 0.12 (0.03, 0.20) | **0.007** | 67.8 | **0.003** | 0.19 | 0.870 |
| CSF | 3 | 0.23 (0.01, 0.44) | **0.039** | 41.5 | 0.181 | 6.86 | 0.155 |
| Blood | 5 | 0.10 (0.00, 0.19) | **0.042** | 76.7 | **0.002** | -0.46 | 0.802 |
| Plasma | 3 | 0.11 (0.01, 0.21) | **0.030** | 72.3 | **0.027** | -1.36 | 0.624 |
| Serum | 2 | - | - | - | - | - | - |
| **3-Hydroxykynurenine** | | | | | | | |
| Overall | 5 | 0.06 (-0.04, 0.16) | 0.238 | 18.9 | 0.294 | -0.30 | 0.789 |
| CSF | 2 | - | - | - | - | - | - |
| Blood | 3 | 0.09 (-0.02, 0.19) | 0.106 | 22.2 | 0.277 | 0.44 | 0.826 |
| Plasma | 2 | - | - | - | - | - | - |
| Serum | 1 | - | - | - | - | - | - |
| **Kynurenic acid** | | | | | | | |
| Overall | 7 | 0.09 (0.00, 0.18) | **0.048** | 55.8 | **0.035** | 0.78 | 0.441 |
| CSF | 3 | 0.29 (0.07, 0.52) | **0.012** | 74.3 | **0.020** | 6.34 | 0.153 |
| Blood | 4 | 0.05 (-0.05, 0.15) | 0.287 | 0.0 | 0.531 | -0.47 | 0.589 |
| Plasma | 3 | 0.07 (-0.03, 0.17) | 0.186 | 0.0 | 0.968 | 0.23 | 0.116 |
| Serum | 1 | - | - | - | **-** | - | - |
| **Anthranilic acid** | | | | | | | |
| Overall | 4 | 0.00 (-0.10, 0.10) | 0.985 | 80.0 | **0.002** | 0.94 | 0.748 |
| CSF | 2 | - | - | - | - | - | - |
| Blood | 2 | - | - | - | - | - | - |
| Plasma | 2 | - | - | - | - | - | - |
| Serum | 0 | - | - | - | - | - | - |
| **3-Hydroxyanthranilic acid** | | | | | | | |
| Overall | 3 | -0.04 (-0.15, 0.06) | 0.403 | 66.2 | **0.052** | 2.45 | 0.261 |
| CSF | 1 | - | - | - | - | - | - |
| Blood | 2 | - | - | - | - | - | - |
| Plasma | 2 | - | - | - | - | - | - |
| Serum | 0 | - | - | - | - | - | - |
| **Picolinic acid** | | | | | | | |
| Overall | 3 | 0.07 (-0.14, 0.27) | 0.506 | 0.0 | 0.816 | -3.35 | 0.509 |
| CSF | 2 | - | - | - | - | - | - |
| Blood | 1 | - | - | - | - | - | - |
| Plasma | 1 | - | - | - | - | - | - |
| Serum | 0 | - | - | - | - | - | - |
| **Quinolinic acid** | | | | | | | |
| Overall | 4 | 0.01 (-0.09, 0.10) | 0.870 | 29.0 | 0.238 | 0.29 | 0.847 |
| CSF | 2 | - | - | - | - | - | - |
| Blood | 2 | - | - | - | - | - | - |
| Plasma | 2 | - | - | - | - | - | - |
| Serum | 0 | - | - | - | - | - | - |

^a^SMD > 0 trend towards higher in MCI, SMD < 0 trend towards lower in MCI. Overall represents levels in CSF and blood combined and Blood represent levels in plasma and serum combined. Statistically significant *p*-values are in bold. Abbreviations: *SMD* standardized mean difference, *CSF* cerebrospinal fluid, *CI* confidence interval.

**Table S6** Summary of Meta-regression on studies investigating differences between patients with MCI and controls.

|  | **Residual variation (I^2^_res_) (%)** | **Adj. R^2^ (%)** | **τ^2^** | **P > \|t\|** | **Prob. > F** |
| --- | --- | --- | --- | --- | --- |
| **Tryptophan** | | | | | |
| Overall (n = 12) | | | | | |
| Year of publication | 59.69 | -4.73 | 0.048 | 0.329 | - |
| Sample size | 61.05 | -67.74 | 0.078 | 0.713 | - |
| Age *(n = 11)* | 56.23 | - | 0.039 | 0.860 | - |
| Sex (% female) | 51.34 | 71.24 | 0.013 | 0.116 | - |
| MCI severity *(n = 10)* | 52.90 | - | 0.006 | 0.577 | - |
| Recruitment of controls | 54.26 | 100.00 | 0.000 | - | 0.337 |
| Biomaterial |  |  |  |  |  |
| CSF/ blood | 61.09 | -54.25 | 0.071 | 0.845 | - |
| CSF/ plasma/ serum | 64.90 | -122.79 | 0.103 | - | 0.862 |
| Analytical technique | 64.70 | -77.55 | 0.082 | - | 0.830 |
|  |  |  |  |  |  |
| CSF (n = 4) | | | | | |
| Year of publication | 79.10 | -55.99 | 0.281 | 0.570 | - |
| Sample size | 81.07 | -94.38 | 0.351 | 0.726 | - |
| Age | 41.54 | 78.06 | 0.040 | 0.204 | - |
| Sex (% female) | 75.74 | -107.22 | 0.374 | 0.906 | - |
| MCI severity *(n = 3)* | 79.71 | -40.03 | 0.505 | 0.552 | - |
| Recruitment of controls | - | - | - | - | - |
| Analytical technique | - | - | - | - | - |
|  | | | | | |
| Blood (n = 8) | | | | | |
| Year of publication | 21.78 | - | 0.000 | 0.054 | - |
| Sample size | 59.38 | - | 0.071 | 0.755 | - |
| Age *(n = 7)* | 44.89 | - | 0.000 | 0.754 | - |
| Sex (% female) | 41.17 | - | 0.000 | 0.146 | - |
| MCI severity *(n = 7)* | 46.00 | - | 0.000 | 0.937 | - |
| Recruitment of controls | 18.86 | - | 0.000 | - | 0.180 |
| Biomaterial |  |  |  |  |  |
| Plasma/ serum | 59.67 | - | 0.067 | 0.643 | - |
| Analytical technique | 66.10 | - | 0.102 | - | 0.986 |
|  |  |  |  |  |  |
| **Kynurenine** | | | | | |
| Overall (n = 7) | | | | | |
| Year of publication | 49.82 | 44.54 | 0.020 | 0.218 | - |
| Sample size | 62.96 | -78.53 | 0.063 | 0.845 | - |
| Age *(n = 6)* | 68.54 | -59.52 | 0.069 | 0.706 | - |
| Sex (% female) | 62.97 | -41.96 | 0.050 | 0.984 | - |
| MCI severity *(n = 5)* | 71.48 | -80.31 | 0.084 | 0.793 | - |
| Recruitment of controls | 70.79 | -136.99 | 0.084 | **-** | 0.723 |
| Biomaterial |  |  |  |  |  |
| CSF/ blood | 62.93 | -56.18 | 0.055 | 0.704 | - |
| CSF/ plasma/ serum | 61.32 | -51.72 | 0.054 | - | 0.538 |
| Analytical technique | 36.58 | 82.34 | 0.006 | - | 0.184 |
|  |  |  |  |  |  |
| CSF (n = 3) | | | | | |
| Year of publication | 0.00 | 100.00 | 0.000 | 0.222 | - |
| Sample size | 0.00 | 100.00 | 0.000 | 0.216 | - |
| Age | 79.24 | 5.43 | 0.125 | 0.477 | - |
| Sex (% female) | 78.61 | 9.38 | 0.120 | 0.468 | - |
| MCI severity | - | - | - | - | - |
| Recruitment of controls | - | - | - | - | - |
| Analytical technique | - | - | - | - | - |
|  |  |  |  |  |  |
| **KTR** | | | | | |
| Overall (n = 8) | | | | | |
| Year of publication | 67.40 | 20.60 | 0.065 | 0.370 | - |
| Sample size | 72.31 | -59.18 | 0.131 | 0.845 | - |
| Age *(n = 7)* | 68.68 | -106.83 | 0.085 | 0.903 | - |
| Sex (% female) *(n = 7)* | 37.81 | 71.93 | 0.013 | 0.072 | - |
| MCI severity *(n = 5)* | 68.30 | -86.89 | 0.070 | 0.991 | - |
| Recruitment of controls | 81.26 | -254.38 | 0.292 | - | 0.983 |
| Biomaterial |  |  |  |  |  |
| CSF/ blood | 70.82 | -35.42 | 0.112 | 0.553 | - |
| CSF/ plasma/ serum | 75.02 | -110.93 | 0.174 | - | 0.705 |
| Analytical technique | 56.51 | 84.43 | 0.013 | - | 0.177 |
|  |  |  |  |  |  |
| Blood (n = 5) |  |  |  |  |  |
| Year of publication | 82.37 | -67.49 | 0.339 | 0.998 | - |
| Sample size | 82.19 | -78.55 | 0.361 | 0.914 | - |
| Age *(n = 4)* | 62.84 | -5.54 | 0.102 | 0.416 | - |
| Sex (% female) *(n = 4)* | 64.08 | 56.29 | 0.037 | 0.252 | - |
| MCI severity *(n = 3)* | 47.38 | -0.91 | 0.024 | 0.468 | - |
| Recruitment of controls | 86.95 | -215.40 | 0.638 | - | 0.985 |
| Biomaterial |  |  |  |  |  |
| Plasma/ serum | 81.92 | -58.43 | 0.321 | 0.589 | - |
| Analytical technique |  |  |  |  |  |
| HPLC/ ELISA | 73.27 | 52.13 | 0.097 | 0.234 | - |
|  |  |  |  |  |  |
| Plasma (n = 3) |  |  |  |  |  |
| Year of publication | 74.34 | 57.20 | 0.114 | 0.339 | - |
| Sample size | 85.97 | -151.49 | 0.671 | 0.737 | - |
| Age | 70.61 | 73.92 | 0.070 | 0.304 | - |
| Sex (% female) | - | - | - | - | - |
| MCI severity | - | - | - | - | - |
| Recruitment of controls | - | - | - | - | - |
| Analytical technique | - | - | - | - | - |
| Anticoagulant | - | - | - | - | - |
|  |  |  |  |  |  |
| **Kynurenic acid** | | | | | |
| Overall (n = 7) | | | | | |
| Year of publication | 61.34 | -112.26 | 0.081 | 0.817 | - |
| Sample size | 60.11 | -160.75 | 0.100 | 0.727 | - |
| Age *(n = 6)* | 52.79 | -95.59 | 0.046 | 0.435 | - |
| Sex (% female) | 56.53 | 12.98 | 0.033 | 0.384 | - |
| MCI severity *(n = 5)* | 27.43 | 100.00 | 0.000 | 0.114 | - |
| Recruitment of controls | 72.21 | -265.57 | 0.140 | - | 0.672 |
| Biomaterial |  |  |  |  |  |
| CSF/ blood | 49.97 | 1.28 | 0.038 | 0.221 | - |
| CSF/ plasma/ serum | 49.07 | 30.92 | 0.026 | - | 0.338 |
| Analytical technique | 12.54 | 100.00 | 0.000 | - | 0.114 |
|  | | | | | |
| CSF (n = 3) | | | | | |
| Year of publication | 0.00 | 100.00 | 0.000 | 0.219 | - |
| Sample size | 59.41 | 67.85 | 0.062 | 0.321 | - |
| Age | 87.12 | -156.54 | 0.494 | 0.880 | - |
| Sex (% female) | 86.30 | -109.21 | 0.403 | 0.714 | - |
| MCI severity | - | - | - | - | - |
| Recruitment of controls | - | - | - | - | - |
| Analytical technique | - | - | - | - | - |
|  |  |  |  |  |  |
| **Anthranilic acid** | | | | | |
| Overall (n = 4) | | | | | |
| Year of publication | 86.44 | -68.84 | 0.197 | 0.852 | - |
| Sample size | 86.39 | -67.58 | 0.196 | 0.832 | - |
| Age | 81.02 | 25.72 | 0.087 | 0.288 | - |
| Sex (% female) | 57.36 | 77.60 | 0.026 | 0.126 | - |
| MCI severity *(n = 3)* | 0.00 | 100.00 | 0.000 | 0.229 | - |
| Recruitment of controls | - | - | - | - | - |
| Biomaterial |  |  |  |  |  |
| CSF/ blood | 86.16 | -52.32 | 0.178 | 0.728 | - |
| CSF/ plasma/ serum | - | - | - | - | - |
| Analytical technique | - | - | - | - | - |
|  |  |  |  |  |  |
| **3-Hydroxyanthranilic acid** | | | | | |
| Overall (n = 3) |  |  |  |  |  |
| Year of publication | 0.00 | 100.00 | 0.000 | 0.269 | - |
| Sample size | 0.00 | 100.00 | 0.000 | 0.269 | - |
| Age | 0.00 | 100.00 | 0.000 | 0.269 | - |
| Sex (% female) | 0.00 | 100.00 | 0.000 | 0.269 | - |
| MCI severity | 0.00 | 100.00 | 0.000 | 0.269 | - |
| Recruitment of controls | - | - | - | - | - |
| Biomaterial |  |  |  |  |  |
| CSF/ blood | 0.00 | 100.00 | 0.000 | 0.269 | - |
| CSF/ plasma/ serum | - | - | - | - | - |
| Analytical technique | - | - | - | - | - |

- = insufficient observations or not applicable. Meta regression with Knapp-Hartung modification was done on metabolites with higher than 50% heterogeneity (*I^2^*). Overall presents kynurenines in CSF and blood combined and Blood presents kynurenines in plasma and serum combined. MMSE scores were used as a proxy for severity of cognitive impairment. Statistically significant *p*-values (2-tailed or F test) are in bold. Abbrevations: *CSF* cerebrospinal fluid, *LC-MS/MS* liquid chromatography with tandem mass spectrometry, *ELISA* enzyme-linked immunosorbent assasy, *HPLC* high performance liquid chromatography, *CSF* cerebrospinal fluid, *adj.* adjusted, *τ^2^,* tau squared.

**Table S7** Cross-sectional associations between kynurenines and MMSE scores in cases and controls.

| **Author** | **Group** | | **Tissue** | **Cov. in analyses** | **Metabolites** | | | | | | | | | |
| --- | --- | --- | --- | --- | --- | --- | --- | --- | --- | --- | --- | --- | --- | --- |
|  | **N** | **Age** |  |  | **TRP** | **KYN** | **3-HK** | **KA** | **XA** | **AA** | **3-HAA** | **PIC** | **QA** | **KTR** |
| Park (2020)^[65]^ | CTRL (40) | 73.9 ± 5.1 | S | None | ns | ns | - | - | - | ns | - | - | - | - |
| Sorgdrager (2019)^[76]^ | AD (33) | 73.7 ± 6.0 | CSF | None | ns | ns | ns | ns | ns | - | - | - | ns | - |
| Sorgdrager (2019)^[76]^ | AD (33) | 73.7 ± 6.0 | S | None | ↑ | ↑ | ns | ns | ns | - | - | - | ↑ | - |
| Wissmann (2013)^[94]^ | AD (43) | 81.7 ± 10.5 | S | None | - | - | - | - | - | - | - | - | - | ↓ |
| Widner (2000)^[92]^ | AD (21) | 74.4 ± 5.4 | S | None | ns | ↓ | - | - | - | - | - | - | - | ↓ |
| Shao (2020)^[74]^ | AD (30) | 71.6 ± 8.8 | P | None | ns | - | - | - | - | - | - | - | - | - |
| Gulaj (2010)^[31]^ | AD (34) | 78.8 ± 5.7 | P | None | ns | ns | ns | ↑ | - | ns | - | - | ns | ns |
| Wennström (2014)^[89]^ | AD (19)  LBD (18)  CTRL (20) | 76.0 | CSF | None | - | - | - | ns | - | - | - | - | - | - |
| Tohgi (1992)^[81]^ | AD (14)  CTRL (10) | 68.4 | CSF | None | ns | ns | ns | - | - | - | - | - | - | ns |
| Whiley (2021)^[90]^ | AD (103)  MCI (165)  CTRL (86) | 77.0 ± 61.3 | S | None | ns | ns | - | - | ↑ | - | - | - | - | - |
| Willette (2021)^[93]^ | AD (112)  MCI (396)  CTRL (58) | unk | S | Age, sex. education | - | - | - | - | - | - | - | - | - | ns |
| Widner (1999)^[91]^ | AD (24)  HD (12)  CTRL (unk) | unk | S | None | ↑ | ns | - | - | - | - | - | - | - | ns |
| Basun (1990)^[11]^ | AD (22)  CTRL (11) | unk | P | None | ns | - | - | - | - | - | - | - | - | - |
| Leblhuber (1998)^[51]^ | HD (12) | 42.4 ± 11.7 | S | None | ↑ | ns | - | - | - | - | - | - | - | ns |
| Gold (2011)^[24]^ | IS (41) | 72.3 ± 12.2 | P | Age | - | - | - | - | - | - | - | - | - | ↓ |

↑ Sig. positive association, ↓ Sig. negative association, *ns* non-significant, *unk* unknown, - metabolite not measured. Abbreviations: *AD* Alzheimer’s dementia, *MCI* Mild Cognitive Impairment, *CTRL* Controls, *LBD* Lewy-body dementia, *HD* Huntington’s disease, *IS* Ischemic stroke patients, *CSF* cerebrospinal fluid, *P* Plasma, *S* Serum, *eGFR* estimated Glomerular Filtration Rate, *PLP* Pyridoxal phosphate, *TRP* Tryptophan, *KYN* Kynurenine, *3-HK* 3-Hydroxykynurenine, *KA* Kynurenic acid, *XA* Xanthurenic acid, *AA* Anthranilic acid, *3-HAA* 3-Hydroxyanthranilic acid, *PIC* Picolinic acid, *QA* Quinolinic acid, *KTR* Kynurenine-Tryptophan ratio.

**Table S8** Cross-sectional associations between kynurenines and other cognitive scores in cases and controls.

| **Author** | **Group** | | **Tissue** | **Cov. in analyses** | **Cognitive test/ domain** | **Metabolites** | | | | | | | | | |
| --- | --- | --- | --- | --- | --- | --- | --- | --- | --- | --- | --- | --- | --- | --- | --- |
|  | **N** | **Age** |  |  |  | **TRP** | **KYN** | **3-HK** | **KA** | **XA** | **AA** | **3-HAA** | **PIC** | **QA** | **KTR** |
| ***Cognitively healthy controls*** | | | | | | | | | | | | | | | |
| Vints (2022)^[87]^ | 74 | 69.4 ± 6.2 | S | Age, education | MoCA | - | ↑ | - | - | - | - | - | - | - | - |
| Huang (2021)^[38]^ | 70 | 39.7 ± 11.8 | S | Age, sex | MCCB total score | - | - | - | ns | - | - | - | - | ns | - |
|  |  |  |  |  | MCCB - Working memory | - | - | - | ns | - | - | - | - | ns | - |
|  |  |  |  |  | MCCB - Processing speed | - | - | - | ns | - | - | - | - | ns | - |
|  |  |  |  |  | MCCB - Attention and vigilance | - | - | - | ns | - | - | - | - | ns | - |
|  |  |  |  |  | MCCB - Verbal Learning | - | - | - | ns | - | - | - | - | ns | - |
|  |  |  |  |  | MCCB - Reasoning and problem solving | - | - | - | ns | - | - | - | - | ns | - |
|  |  |  |  |  | MCCB - Social cognition | - | - | - | ns | - | - | - | - | ns | - |
| Parker (2023)^[66]^ | 301 | 74.8 ± 8.7 | P | Age, sex | MoCA | ↑ | ns | - | - | - | - | - | - | - | ns |
| Chatterjee (2020)^[15]^ | 100 | 78.2 ± 5.5 | P | Age, sex, APOE ε4 status, BMI | Global cognition^b^ | ns | ns | - | - | - | - | - | - | - | - |
| Kindler (2020)^[47]^ | 81 | 31.7 | P | None | Working memory | - | - | - | - | - | - | - | - | - | ns |
|  |  |  |  |  | Attention | - | - | - | - | - | - | - | - | - | ns |
| Hafstad Solvang (2019)^[33]^ | 2174 | 71^a^ | P | Age, sex, BMI, education, eGFR, smoking, diabetes, hypertension, myocardial infarction, stroke, PLP, CRP, NSAIDS | COWAT | ns | ns | ns | ns | ns | ns | ns | ns | ns | ↓ |
|  |  |  |  |  | DST | ns | ns | ns | ns | ns | ns | ns | ns | ns | ns |
|  |  |  |  |  | KOLT | ns | ns | ns | ns | ns | ns | ns | ns | ns | ↓ |
| ***Dementia (all types)*** | | | | | | | | | | | | | | | |
| Gulaj (2010)^[31]^ | AD (34) | 78.8 ± 5.7 | P | None | CDT | ns | ns | ns | ns |  | ns | - | - | ↓ | ns |
| Mourdian (1989)^[61]^ | AD (35) | 64.0 ± 5.9 | CSF | None | DRS | - | - | - | - | - | - | - | - | ns | - |
|  |  |  |  |  | WMS | - | - | - | - | - | - | - | - | ns | - |
| Nho (2021)^[62]^ | AD (304)  Late MCI (491)  Early MCI (271)  SCD (95)  CTRL (370) | 74.8 ± 7.8  74.0 ± 7.6  71.3 ± 7.6  72.3 ± 5.7  74.6 ± 5.8 | S | Age, sex, BMI, APOE ε4 status, study phase | Memory compound | - | ↑ | - | - | - | - | - | - | - | - |
|  |  |  |  |  | Executive funct. compound | - | ↑ | - | - | - | - | - | - | - | - |
| Toledo (2017)^[83]^ | AD (175)  MCI (356)  CTRL (199) | 75.3 | S | Age, gender, education, APOE ε4 status | ADAS-Cog13 | ns | ns | - | - | - | - | - | - | - | - |
| Basun (1990)^[11]^ | AD (22)  CTRL (11) | unk | P | None | Memory (CDR) | ns | - | - | - | - | - | - | - | - | - |
|  |  |  |  |  | Orientation (CDR) | ns | - | - | - | - | - | - | - | - | - |
| Küster (2017)^[50]^ | DEM (4)  MCI (32)  SCD (11) | 71.2 ± 6.0 | S | Age, education, alcohol consumption | Global cognition^d^ | - | ns | ns | ns | - | - | - | - | ns | - |
|  |  |  |  |  | Memory compound | - | ns | ns | ns | - | - | - | - | ns | - |
|  |  |  |  |  | Executive funct. compound | - | ns | ns | ns | - | - | - | - | ↓ | - |
| Willette (2021)^[93]^ | AD (112)  MCI (396)  CTRL (58) | unk | S | Age, sex. education | RAVLT Trials 1-5 | - | - | - | - | - | - | - | - | - | ↓ |
|  |  |  |  |  | Short delay memory | - | - | - | - | - | - | - | - | - | ↓ |
|  |  |  |  |  | Items forgotten during long delay | - | - | - | - | - | - | - | - | - | ↑ |
|  |  |  |  |  | Memory factor | - | - | - | - | - | - | - | - | - | ↓ |

↑ Sig. positive association, ↓ Sig. negative association, *ns* non-significant, *unk* unknown, - metabolite not measured. ^a^Age in median. ^b^Composite score of verbal and visual episodic memory, working memory and executive functioning. ^d^Average of two component scores (memory functions and attention/ executive functions) as determined by principal component analysis. Abbreviations: *CTRL* Controls, *AD* Alzheimer’s dementia, *MCI* Mild Cognitive Impairment, *DEM* Dementia, *SCD* Subjective Cognitive Decline, *CSF* cerebrospinal fluid, *P* Plasma, *S* Serum, *MoCA* the Montreal Cognitive Assessment, *MCCB* MATRICS Consensus Cognitive Battery, *eGFR* estimated Glomerular Filtration Rate, *PLP* Pyridoxal phosphate, *CRP* C-reactive protein, *NSAIDS* Non-Steroidal Anti-inflammatory Drugs, *CDT* Clock Drawing Test, *DRS* Mattis Dementia Rating Scale, *WMS* Wechsler Memory Scale, *ADAS-Cog13* Alzheimer’s Disease Assessment Scale cognitive subscale, *CDR* Clinical Dementia Rating Scale, *COWAT* Controlled Oral Word Association Test, *DST* Digit Symbol Test, *KOLT* Kendrick Object Learning Test, *RAVLT* Rey Auditory Verbal Learning Test, *TRP* Tryptophan, *KYN* Kynurenine, *3-HK* 3-Hydroxykynurenine, *KA* Kynurenic acid, *XA* Xanthurenic acid, *AA* Anthranilic acid, *3-HAA* 3-Hydroxyanthranilic acid, *PIC* Picolinic acid, *QA* Quinolinic acid, *KTR* Kynurenine-Tryptophan ratio.

**Table S9** Cross-sectional associations between kynurenine ratios or neopterin and cognitive scores in samples of patients and controls.

| **Author** | **Group** | | **Tissue** | **Cov. in analyses** | **Cognitive test/ domain** | **Ratios** | | | | | | | | | | **Neop** |
| --- | --- | --- | --- | --- | --- | --- | --- | --- | --- | --- | --- | --- | --- | --- | --- | --- |
|  | **N** | **Age** |  |  |  | **HK/**  **TRP** | **HK/**  **KYN** | **HK/KA** | **KYN/ HK** | **AA/**  **KYN** | **KYN/ KA** | **KA/**  **KYN** | **KA/QA** | **QA/KA** | **QA/**  **HK** |  |
| ***Cognitively healthy controls*** | | | | | | | | | | | | | | | | |
| Anderson (2021)^[1]^ | 74 | 36.2 ± 9.4 | S | None | ANAM – Reaction time | - | - | - | - | - | - | - | - | ns | - | - |
|  |  |  |  |  | ANAM – Visuospatial processing | - | - | - | - | - | - | - | - | ns | - | - |
|  |  |  |  |  | ANAM – Working memory and visuospatial processing | - | - | - | - | - | - | - | - | ns | - | - |
|  |  |  |  |  | ANAM – Attention and vigilance | - | - | - | - | - | - | - | - | ns | - | - |
|  |  |  |  |  | ANAM – Visuospatial skills | - | - | - | - | - | - | - | - | ns | - | - |
|  |  |  |  |  | Working memory | - | - | - | - | - | - | - | - | ns | - | - |
|  |  |  |  |  | Spatial memory | - | - | - | - | - | - | - | - | ns | - | - |
| Huang (2021)^[38]^ | 70 | 39.7 ± 11.8 | S | Age, sex | MCCB total score | - | - | - | - | - | - | - | - | ns | - | - |
|  |  |  |  |  | MCCB - Working memory | - | - | - | - | - | - | - | - | ns | - | - |
|  |  |  |  |  | MCCB - Processing speed | - | - | - | - | - | - | - | - | ns | - | - |
|  |  |  |  |  | MCCB - Attention and vigilance | - | - | - | - | - | - | - | - | ns | - | - |
|  |  |  |  |  | MCCB - Verbal Learning | - | - | - | - | - | - | - | - | ns | - | - |
|  |  |  |  |  | MCCB - Reasoning and problem solving | - | - | - | - | - | - | - | - | ns | - | - |
|  |  |  |  |  | MCCB - Social cognition | - | - | - | - | - | - | - | - | ns | - | - |
| Platzer (2017)^[68]^ | Men (36) | 38.1 ± 15.1 | S | Age, BMI, education, smoking, CVD | MWTB | - | - | ns | ns | - | ns | - | - | - | - | - |
|  |  |  |  |  | TMT A | - | - | ns | ns | - | ns | - | - | - | - | - |
|  |  |  |  |  | TMT B | - | - | ns | ns | - | ns | - | - | - | - | - |
|  |  |  |  |  | d2 test | - | - | ns | ns | - | ns | - | - | - | - | - |
|  |  |  |  |  | Stroop 1 | - | - | ns | ns | - | ns | - | - | - | - | - |
|  |  |  |  |  | Stroop 2 | - | - | ns | ns | - | ns | - | - | - | - | - |
|  |  |  |  |  | Stroop interference | - | - | ns | ns | - | ns | - | - | - | - | - |
|  |  |  |  |  | CVLT Trial 1-5 | - | - | ns | ns | - | ns | - | - | - | - | - |
|  |  |  |  |  | CVLT short delay recall | - | - | ns | ns | - | ns | - | - | - | - | - |
|  |  |  |  |  | CVLT long delayed recall | - | - | ns | ns | - | ns | - | - | - | - | - |
|  | Women (57) | 39.4 ± 16.9 |  |  | MWTB | - | - | ns | ns | - | ns | - | - | - | - | - |
|  |  |  |  |  | TMT A | - | - | ns | ns | - | ns | - | - | - | - | - |
|  |  |  |  |  | TMT B | - | - | ns | ns | - | ns | - | - | - | - | - |
|  |  |  |  |  | d2 test | - | - | ns | ns | - | ns | - | - | - | - | - |
|  |  |  |  |  | Stroop 1 | - | - | ns | ns | - | ns | - | - | - | - | - |
|  |  |  |  |  | Stroop 2 | - | - | ns | ns | - | ns | - | - | - | - | - |
|  |  |  |  |  | Stroop interference | - | - | ns | ns | - | ns | - | - | - | - | - |
|  |  |  |  |  | CVLT Trial 1-5 | - | - | ns | ns | - | ns | - | - | - | - | - |
|  |  |  |  |  | CVLT short delay recall | - | - | ns | ns | - | ↓ | - | - | - | - | - |
|  |  |  |  |  | CVLT long delayed recall | - | - | ns | ns | - | ns | - | - | - | - | - |
| Parker (2023)^[66]^ | 301 | 74.8 ± 8.7 | P | Age, sex | MoCA | - | - | - | - | - | - | ns | - | - | - | - |
| Hafstad Solvang (2019)^[33]^ | 2174 | 71^a^ | P | Age, sex, BMI, education, eGFR, smoking, diabetes, hypertension, myocardial infarction, stroke, PLP, CRP, NSAIDS | COWAT | - | - | - | - | - | - | - | - | - | - | ↓ |
|  |  |  |  |  | DST | - | - | - | - | - | - | - | - | - | - | ns |
|  |  |  |  |  | KOLT | - | - | - | - | - | - | - | - | - | - | ↓ |
| ***Dementia (all types)*** | | | | | | | | | | | | | | | | |
| Wissmann (2013)^[94]^ | AD (43) | 81.7 ± 10.5 | S | None | MMSE | - | - | - | - | - | - | - | - | - | - | ↓ |
|  |  |  |  |  | CDT | - | - | - | - | - | - | - | - | - | - | ↓ |
| Gulaj (2010)^[31]^ | AD (34) | 78.8 ± 5.7 | P | None | MMSE | - | ns | - | - | ns | - | ↑ | - | - | ns | - |
|  |  |  |  |  | CDT | - | ns | - | - | ns | - | ns | - | - | ns | - |
| Widner (1999)^[91]^ | AD (24)  HD (12)  CTRL (unk) | unk | S | None | MMSE | - | - | - | - | - | - | - | - | - | - | ns |
| Leblhuber (1998)^[51]^ | HD (12) | 42.4 ± 11.7 | S | None | MMSE | - | - | - | - | - | - | - | - | - | - | ns |
| Tohgi (1992)^[81]^ | AD (14)  CTRL (10) | 68.4 | CSF | None | MMSE | ns | - | - | - | - | - | - | - | - | - | - |

↑ positive association, ↓ negative association, *ns* non-significant, *unk* unknown, ↑↓ non-linear association, - metabolite not measured. ^a^Age in median. Abbreviations: *AD* Alzheimer’s dementia, *CTRL* Normal Controls, *CSF* cerebrospinal fluid, *P* Plasma, *S* Serum, *eGFR* estimated Glomerular Filtration Rate, *PLP* Pyridoxal phosphate, *CRP* C-reactive protein, *NSAIDS* Non-Steroidal Anti-inflammatory Drugs, *MMSE* Mini Mental State Examination, *ANAM* Automated Neuropsychological Assessment Metrics, *MCCB* MATRICS Consensus Cognitive Battery, *MWTB* Multiple Choice Word test, *TMT* Trail Making test, *CVLT* California Verbal Learning test, *MoCA* the Montreal Cognitive Assessment, *CDT* Clock Drawing Test, *COWAT* Controlled Oral Word Association Test, *DST* Digit Symbol Test, *KOLT* Kendrick Object Learning Test, *TRP* Tryptophan, *KYN* Kynurenine, *HK* 3-Hydroxy-Kynurenine, *KA* Kynurenic acid, *AA* Anthranilic acid, *QA* Quinolinic acid, *Neop* Neopterin.

**Table S10** Quality assessment of data included in systematic review.

| **Study**^a^ | **Selection** | | | | **Comparability** | | | **Exposure** | | | **Total** |
| --- | --- | --- | --- | --- | --- | --- | --- | --- | --- | --- | --- |
|  | **1** | **2** | **3** | **4** | **1a** | **1b** | **1** | | **2** | **3** |  |
| *Case-control data* |  | | | |  | | |  | | |  |
| Aquilani (2023)^[2]^ | 1 | 0 | 0 | 1 | 1 | 0 | 1 | | 1 | 0 | 5 |
| Arai (1984)^[3]^ | 1 | 0 | 0 | 0 | 1 | 1 | 1 | | 1 | 0 | 5 |
| Arai (1985)^[4]^ | 1 | 0 | 0 | 0 | 1 | 1 | 1 | | 1 | 0 | 5 |
| Atukeren (2017)^[5]^ | 1 | 0 | 0 | 0 | 1 | 1 | 1 | | 1 | 0 | 5 |
| Baker (1989)^[6]^ | 1 | 0 | 0 | 1 | 1 | 1 | 1 | | 1 | 0 | 6 |
| Bakker (2021)^[7]^ | 1 | 0 | 1 | 1 | 1 | 1 | 1 | | 1 | 0 | 7 |
| Bakker (2023)^[8]^ | 1 | 0 | 0 | 1 | 1 | 1 | 1 | | 1 | 0 | 6 |
| Baran (1999)^[10]^ | 0 | 0 | 0 | 0 | 1 | 0 | 0 | | 1 | 0 | 2 |
| Basun (1990)^[11]^ | 1 | 0 | 1 | 1 | 1 | 0 | 1 | | 1 | 0 | 6 |
| Beal (1990)^[12]^ | 1 | 0 | 0 | 1 | 0 | 0 | 1 | | 1 | 0 | 4 |
| Bonaccorso (1998)^[13]^ | 1 | 0 | 0 | 1 | 1 | 1 | 1 | | 1 | 0 | 6 |
| Cogo (2021) ^[17]^ | 0 | 0 | 0 | 0 | 0 | 0 | 1 | | 1 | 0 | 2 |
| Czech (2012)^[18]^ | 1 | 0 | 0 | 0 | 1 | 0 | 1 | | 0 | 0 | 3 |
| de Leeuw (2017)^[19]^ | 1 | 0 | 0 | 0 | 1 | 1 | 1 | | 1 | 0 | 5 |
| Fakhruddin (2020^)[20]^ | 1 | 0 | 1 | 1 | 1 | 0 | 0 | | 1 | 0 | 5 |
| Fekkes (1998)^[21]^ | 1 | 1 | 0 | 1 | 1 | 0 | 1 | | 0 | 0 | 5 |
| Fonteh (2007)^[22]^ | 1 | 0 | 0 | 0 | 1 | 1 | 1 | | 1 | 0 | 5 |
| Giil (2017)^[23]^ | 1 | 1 | 1 | 0 | 1 | 1 | 1 | | 1 | 0 | 7 |
| Gonzalez-Dominguez (2014)^[25]^ | 1 | 0 | 0 | 0 | 1 | 1 | 1 | | 0 | 0 | 4 |
| Gonzalez-Dominguez (2015a)^[26]^ | 1 | 1 | 0 | 0 | 0 | 0 | 1 | | 1 | 0 | 4 |
| Gonzalez-Dominguez (2015b)^[27]^ | 1 | 0 | 0 | 0 | 0 | 0 | 1 | | 1 | 0 | 3 |
| González-Sánchez (2020)^[28]^ | 1 | 1 | 1 | 1 | 0 | 0 | 1 | | 0 | 0 | 5 |
| Graham (2015)^[29]^ | 1 | 0 | 1 | 0 | 1 | 0 | 1 | | 1 | 0 | 5 |
| Greilberger (2010)^[30]^ | 1 | 1 | 0 | 0 | 1 | 0 | 1 | | 0 | 0 | 4 |
| Gulaj (2010)^[31]^ | 1 | 0 | 0 | 0 | 1 | 1 | 1 | | 1 | 0 | 5 |
| Hartai (2007)^[34]^ | 1 | 0 | 0 | 0 | 1 | 1 | 1 | | 0 | 0 | 4 |
| Heyes (1992)^[36]^ | 0 | 0 | 1 | 0 | 0 | 0 | 0 | | 0 | 0 | 1 |
| Heylen (2023)^[37]^ | 1 | 0 | 0 | 0 | 1 | 0 | 1 | | 0 | 0 | 3 |
| Ibáñez (2013)^[40]^ | 1 | 0 | 0 | 0 | 0 | 0 | 1 | | 1 | 0 | 3 |
| Ikeuchi (2022)^[41]^ | 1 | 0 | 1 | 1 | 1 | 0 | 1 | | 1 | 0 | 6 |
| Jacobs (2019)^[42]^ | 1 | 1 | 0 | 0 | 1 | 0 | 1 | | 1 | 0 | 5 |
| Janssens (2020^)[43]^ | 0 | 0 | 0 | 0 | 1 | 0 | 1 | | 1 | 0 | 3 |
| Kaddurah-Daouk (2011)^[44]^ | 1 | 0 | 0 | 0 | 1 | 1 | 1 | | 1 | 0 | 5 |
| Kaddurah-Daouk (2013)^[45]^ | 1 | 0 | 0 | 0 | 1 | 1 | 1 | | 1 | 0 | 5 |
| Klatt (2021)^[48]^ | 0 | 0 | 0 | 0 | 1 | 0 | 0 | | 0 | 0 | 1 |
| Knapskog (2023)^[49]^ | 1 | 1 | 0 | 0 | 1 | 1 | 1 | | 1 | 0 | 6 |
| Li (2010)^[52]^ | 0 | 0 | 0 | 0 | 1 | 1 | 1 | | 1 | 0 | 4 |
| Liang (2016)^[53]^ | 1 | 0 | 0 | 0 | 0 | 0 | 1 | | 1 | 0 | 3 |
| Lin (2019)^[54]^ | 1 | 0 | 0 | 0 | 0 | 0 | 1 | | 1 | 0 | 3 |
| Liu (2015)^[55]^ | 1 | 0 | 0 | 1 | 0 | 0 | 1 | | 1 | 0 | 4 |
| Liu (2023)^[56]^ | 1 | 0 | 0 | 0 | 1 | 0 | 1 | | 0 | 0 | 3 |
| Martinez (1993)^[57]^ | 1 | 0 | 0 | 1 | 1 | 0 | 1 | | 1 | 0 | 5 |
| Molina (1998)^[60]^ | 1 | 1 | 0 | 0 | 1 | 1 | 1 | | 0 | 0 | 5 |
| Mourdian (1989)^[61]^ | 1 | 0 | 0 | 0 | 1 | 0 | 1 | | 0 | 0 | 3 |
| Oxenkrug (2017)^[63]^ | 0 | 0 | 0 | 0 | 1 | 1 | 0 | | 0 | 0 | 2 |
| Paglia (2016)^[64]^ | 1 | 0 | 0 | 0 | 1 | 1 | 0 | | 0 | 0 | 3 |
| Peña-Bautista (2020)^[67]^ | 1 | 0 | 0 | 0 | 1 | 1 | 1 | | 1 | 0 | 5 |
| Ramos-Chavez (2018)^[69]^ | 1 | 0 | 0 | 1 | 1 | 1 | 1 | | 1 | 0 | 6 |
| Rommer (2016)^[70]^ | 1 | 1 | 0 | 0 | 1 | 0 | 1 | | 0 | 0 | 4 |
| Rudman (1989)^[71]^ | 1 | 0 | 1 | 0 | 1 | 0 | 0 | | 0 | 0 | 3 |
| Santos (2020)^[72]^ | 1 | 0 | 1 | 0 | 1 | 1 | 1 | | 0 | 0 | 5 |
| Schwarz (2013)^[73]^ | 1 | 1 | 0 | 0 | 1 | 1 | 1 | | 1 | 0 | 6 |
| Shao (2020)^[74]^ | 1 | 0 | 0 | 0 | 0 | 1 | 1 | | 0 | 0 | 3 |
| Shaw (1981)^[75]^ | 0 | 0 | 0 | 0 | 0 | 1 | 0 | | 0 | 0 | 1 |
| Sorgdrager (2019)^[76]^ | 1 | 0 | 0 | 0 | 1 | 1 | 1 | | 0 | 0 | 4 |
| Storga (1996)^[77]^ | 1 | 0 | 0 | 1 | 0 | 0 | 1 | | 1 | 0 | 4 |
| Tarbit (1980)^[78]^ | 0 | 0 | 0 | 0 | 0 | 0 | 1 | | 0 | 0 | 1 |
| Teruya (2021)^[79]^ | 1 | 0 | 1 | 0 | 0 | 0 | 1 | | 0 | 0 | 3 |
| Thomas (1986)^[80]^ | 1 | 0 | 1 | 0 | 1 | 1 | 1 | | 1 | 0 | 6 |
| Tohgi (1992)^[81]^ | 1 | 0 | 0 | 0 | 1 | 0 | 1 | | 0 | 0 | 3 |
| Tohgi (1995)^[82]^ | 1 | 0 | 0 | 0 | 1 | 0 | 1 | | 0 | 0 | 3 |
| Trushina (2013)^[84]^ | 1 | 0 | 1 | 0 | 0 | 0 | 1 | | 1 | 0 | 4 |
| Tsuruoka (2013)^[85]^ | 1 | 1 | 0 | 0 | 1 | 0 | 1 | | 0 | 0 | 4 |
| Van der Velpen (2019)^[86]^ | 1 | 1 | 1 | 0 | 0 | 0 | 1 | | 1 | 0 | 5 |
| Watkins (1989)^[88]^ | 1 | 0 | 1 | 0 | 1 | 1 | 1 | | 1 | 0 | 6 |
| Wennström (2014)^[89]^ | 1 | 1 | 1 | 0 | 1 | 0 | 1 | | 1 | 0 | 6 |
| Whiley (2021)^[90]^ | 1 | 0 | 0 | 0 | 1 | 0 | 1 | | 1 | 0 | 4 |
| Widner (1999)^[91]^ | 1 | 0 | 0 | 0 | 0 | 0 | 0 | | 1 | 0 | 2 |
| Widner (2000)^[92]^ | 1 | 0 | 0 | 0 | 1 | 0 | 1 | | 0 | 0 | 3 |
| Willette (2021)^[93]^ | 1 | 0 | 0 | 0 | 1 | 1 | 1 | | 1 | 0 | 5 |
| Wu (2021)^[95]^ | 1 | 0 | 1 | 0 | 1 | 1 | 1 | | 1 | 0 | 6 |
| Xu (2016)^[96]^ | 1 | 0 | 0 | 0 | 1 | 1 | 1 | | 0 | 0 | 4 |
| Xu (2021)^[97]^ | 1 | 0 | 0 | 0 | 1 | 1 | 1 | | 1 | 0 | 5 |
| Yilmaz (2020)^[98]^ | 1 | 0 | 1 | 0 | 1 | 1 | 1 | | 1 | 0 | 6 |
|  |  |  |  |  |  |  |  | |  |  |  |
| *Different patient populations*^b^ |  | | | |  | | |  | | |  |
| Hafstad Solvang (2019)^[32]^ | 1 | 0 | 1 | 0 | 1 | 1 | 1 | | 1 | 0 | 6 |
| Kaiser (2010)^[46]^ | 1 | 0 | 1 | 0 | 0 | 0 | 1 | | 1 | 0 | 4 |
| Mashige (1993)^[58]^ | 0 | 0 | 0 | 0 | 0 | 0 | 1 | | 1 | 0 | 2 |
|  |  |  |  |  |  |  |  | |  |  |  |
| *Cross-sectional data*^b^ |  | | | |  | | |  | | |  |
| Anderson (2021)^[1]^ | 1 | 0 | 0 | 1 | 1 | 1 | 1 | | 1 | 1 | 7 |
| Chatterjee (2020)^[15]^ | 1 | 0 | 1 | 1 | 0 | 1 | 1 | | 1 | 1 | 7 |
| Gold (2011)^[24]^ | 1 | 0 | 1 | 1 | 1 | 1 | 1 | | 1 | 0 | 7 |
| Hafstad Solvang (2019)^[33]^ | 1 | 1 | 1 | 1 | 1 | 1 | 1 | | 1 | 1 | 9 |
| Huang (2021)^[38]^ | 1 | 0 | 1 | 1 | 1 | 1 | 1 | | 1 | 1 | 8 |
| Kindler (2020)^[47]^ | 1 | 0 | 1 | 1 | 1 | 1 | 1 | | 0 | 1 | 7 |
| Küster (2017)^[50]^ | 1 | 0 | 1 | 1 | 0 | 0 | 1 | | 0 | 0 | 4 |
| Leblhuber (1998)^[51]^ | 1 | 0 | 0 | 1 | 0 | 0 | 1 | | 1 | 1 | 5 |
| Park (2020)^[65]^ | 1 | 0 | 1 | 1 | 0 | 0 | 1 | | 1 | 1 | 6 |
| Platzer (2017)^[68]^ | 1 | 0 | 1 | 1 | 1 | 1 | 1 | | 1 | 1 | 8 |
| Vints (2022)^[87]^ | 1 | 0 | 1 | 1 | 1 | 1 | 1 | | 1 | 1 | 8 |
| Wissmann (2013)^[94]^ | 0 | 0 | 1 | 1 | 0 | 0 | 1 | | 1 | 1 | 5 |
|  |  |  |  |  |  |  |  | |  |  |  |
| *Cohort data* |  | | | |  | | |  | | |  |
| Bakker (2023)^[9]^ | 1 | 1 | 1 | 0 | 1 | 1 | 1 | | 1 | 0 | 7 |
| Cespedes (2022)^[14]^ | 1 | 1 | 1 | 1 | 1 | 1 | 1 | | 1 | 1 | 9 |
| Chouraki (2017)^[16]^ | 1 | 1 | 1 | 1 | 1 | 1 | 1 | | 1 | 1 | 9 |
| Hebbrecht (2022)^[35]^ | 1 | 1 | 1 | 0 | 1 | 1 | 1 | | 0 | 0 | 6 |
| Huo (2020)^[39]^ | 1 | 1 | 1 | 1 | 1 | 1 | 1 | | 1 | 1 | 9 |
| McCann (2021)^[59]^ | 1 | 1 | 1 | 0 | 1 | 1 | 1 | | 1 | 0 | 7 |
| Nho (2021)^[62]^ | 0 | 1 | 1 | 0 | 1 | 1 | 1 | | 1 | 0 | 6 |
| Parker (2023)^[66]^ | 1 | 1 | 1 | 0 | 1 | 1 | 1 | | 1 | 1 | 8 |
| Toledo (2017)^[83]^ | 1 | 1 | 1 | 0 | 1 | 1 | 1 | | 1 | 0 | 7 |

*n/a* not applicable. ^a^Indicates the design of the data included in this systematic review and does not per definition correspond to the design of the original study to which these data belong. ^b^Assessed by using an adapted scale of the NOS for case-control studies, see appendix S3 and S4.

**Table S11** PRISMA 2020 Checklist

| **Section and Topic** | **Item #** | **Checklist item** | **Location where item is reported** |
| --- | --- | --- | --- |
| **TITLE** | | |  |
| Title | 1 | Identify the report as a systematic review. | 1 |
| **ABSTRACT** | | |  |
| Abstract | 2 | See the PRISMA 2020 for Abstracts checklist. | 3 |
| **INTRODUCTION** | | |  |
| Rationale | 3 | Describe the rationale for the review in the context of existing knowledge. | 5, 6 |
| Objectives | 4 | Provide an explicit statement of the objective(s) or question(s) the review addresses. | 5, 6 |
| **METHODS** | | |  |
| Eligibility criteria | 5 | Specify the inclusion and exclusion criteria for the review and how studies were grouped for the syntheses. | 7, 8 |
| Information sources | 6 | Specify all databases, registers, websites, organisations, reference lists and other sources searched or consulted to identify studies. Specify the date when each source was last searched or consulted. | 6, 7 |
| Search strategy | 7 | Present the full search strategies for all databases, registers and websites, including any filters and limits used. | 6, 7 |
| Selection process | 8 | Specify the methods used to decide whether a study met the inclusion criteria of the review, including how many reviewers screened each record and each report retrieved, whether they worked independently, and if applicable, details of automation tools used in the process. | 7, 8 |
| Data collection process | 9 | Specify the methods used to collect data from reports, including how many reviewers collected data from each report, whether they worked independently, any processes for obtaining or confirming data from study investigators, and if applicable, details of automation tools used in the process. | 7 |
| Data items | 10a | List and define all outcomes for which data were sought. Specify whether all results that were compatible with each outcome domain in each study were sought (e.g. for all measures, time points, analyses), and if not, the methods used to decide which results to collect. | 7, 8 |
|  | 10b | List and define all other variables for which data were sought (e.g. participant and intervention characteristics, funding sources). Describe any assumptions made about any missing or unclear information. | 7, 8 |
| Study risk of bias assessment | 11 | Specify the methods used to assess risk of bias in the included studies, including details of the tool(s) used, how many reviewers assessed each study and whether they worked independently, and if applicable, details of automation tools used in the process. | 9, 10 |
| Effect measures | 12 | Specify for each outcome the effect measure(s) (e.g. risk ratio, mean difference) used in the synthesis or presentation of results. | 8, 9 |
| Synthesis methods | 13a | Describe the processes used to decide which studies were eligible for each synthesis (e.g. tabulating the study intervention characteristics and comparing against the planned groups for each synthesis (item #5)). | 7, 8 |
|  | 13b | Describe any methods required to prepare the data for presentation or synthesis, such as handling of missing summary statistics, or data conversions. | 8, 9 |
|  | 13c | Describe any methods used to tabulate or visually display results of individual studies and syntheses. | 8, 9 |
|  | 13d | Describe any methods used to synthesize results and provide a rationale for the choice(s). If meta-analysis was performed, describe the model(s), method(s) to identify the presence and extent of statistical heterogeneity, and software package(s) used. | 8, 9 |
|  | 13e | Describe any methods used to explore possible causes of heterogeneity among study results (e.g. subgroup analysis, meta-regression). | 9 |
|  | 13f | Describe any sensitivity analyses conducted to assess robustness of the synthesized results. | N/A |
| Reporting bias assessment | 14 | Describe any methods used to assess risk of bias due to missing results in a synthesis (arising from reporting biases). | 8, 9 |
| Certainty assessment | 15 | Describe any methods used to assess certainty (or confidence) in the body of evidence for an outcome. | 9, 10 |
| **RESULTS** | | |  |
| Study selection | 16a | Describe the results of the search and selection process, from the number of records identified in the search to the number of studies included in the review, ideally using a flow diagram. | 10, 11 |
|  | 16b | Cite studies that might appear to meet the inclusion criteria, but which were excluded, and explain why they were excluded. | 10, 11 |
| Study characteristics | 17 | Cite each included study and present its characteristics. | Supp. Table 1 |
| Risk of bias in studies | 18 | Present assessments of risk of bias for each included study. | 20 |
| Results of individual studies | 19 | For all outcomes, present, for each study: (a) summary statistics for each group (where appropriate) and (b) an effect estimate and its precision (e.g. confidence/credible interval), ideally using structured tables or plots. | 18-20 |
| Results of syntheses | 20a | For each synthesis, briefly summarise the characteristics and risk of bias among contributing studies. | 18-20 |
|  | 20b | Present results of all statistical syntheses conducted. If meta-analysis was done, present for each the summary estimate and its precision (e.g. confidence/credible interval) and measures of statistical heterogeneity. If comparing groups, describe the direction of the effect. | 18-20 |
|  | 20c | Present results of all investigations of possible causes of heterogeneity among study results. | 18-20 |
|  | 20d | Present results of all sensitivity analyses conducted to assess the robustness of the synthesized results. | N/A |
| Reporting biases | 21 | Present assessments of risk of bias due to missing results (arising from reporting biases) for each synthesis assessed. | 18-20 |
| Certainty of evidence | 22 | Present assessments of certainty (or confidence) in the body of evidence for each outcome assessed. | 18-20 |
| **DISCUSSION** | | |  |
| Discussion | 23a | Provide a general interpretation of the results in the context of other evidence. | 20-25 |
|  | 23b | Discuss any limitations of the evidence included in the review. | 26, 27 |
|  | 23c | Discuss any limitations of the review processes used. | 26, 27 |
|  | 23d | Discuss implications of the results for practice, policy, and future research. | 27 |
| **OTHER INFORMATION** | | |  |
| Registration and protocol | 24a | Provide registration information for the review, including register name and registration number, or state that the review was not registered. | 6, 7, 28 |
|  | 24b | Indicate where the review protocol can be accessed, or state that a protocol was not prepared. | 6, 7 |
|  | 24c | Describe and explain any amendments to information provided at registration or in the protocol. | 6, 7 |
| Support | 25 | Describe sources of financial or non-financial support for the review, and the role of the funders or sponsors in the review. | 28 |
| Competing interests | 26 | Declare any competing interests of review authors. | 28 |
| Availability of data, code and other materials | 27 | Report which of the following are publicly available and where they can be found: template data collection forms; data extracted from included studies; data used for all analyses; analytic code; any other materials used in the review. | 28, Supp. Material |

*From:*  Page MJ, McKenzie JE, Bossuyt PM, Boutron I, Hoffmann TC, Mulrow CD, The PRISMA 2020 statement: an updated guideline for reporting systematic reviews. BMJ 2021;372:n71. doi: 10.1136/bmj.n71

For more information, visit: <http://www.prisma-statement.org/>

A B

**
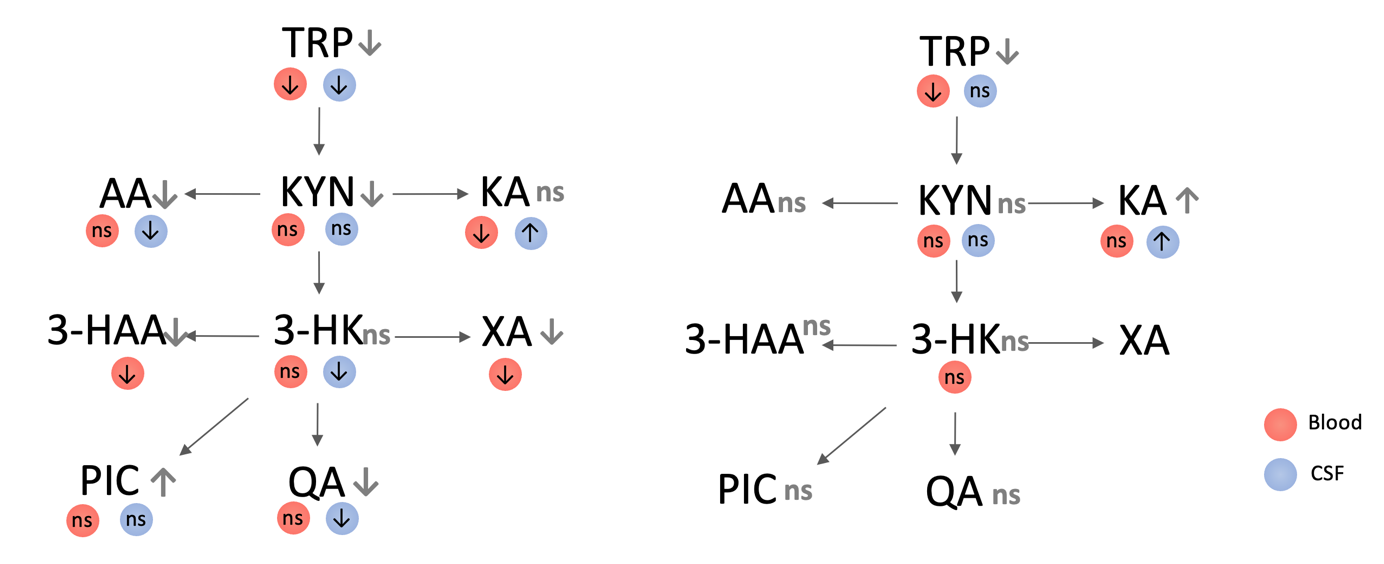
**

**Figure S1** Results of meta-analyses comparing kynurenines between cases and controls.

Overall differences are shown in grey.

A) AD dementia versus controls, B) MCI versus controls. ↑ Sig. higher in cases, ↓ Sig. lower in cases, ns non-significant.

| A  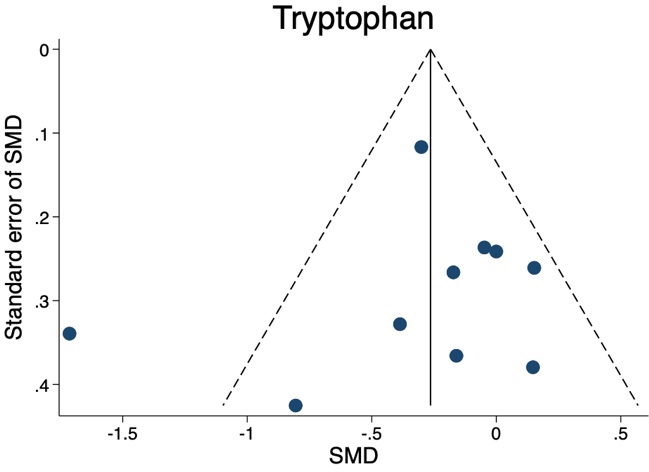 | B  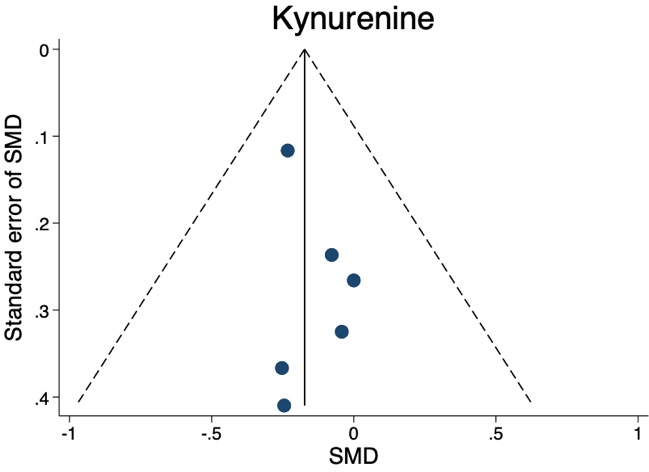 |
| --- | --- |
| C  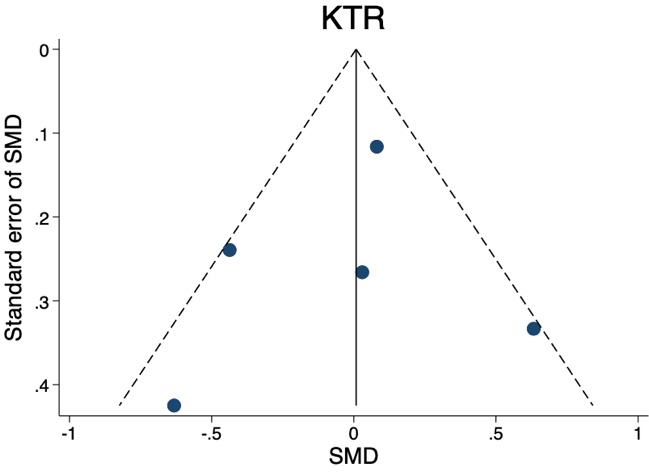 | D  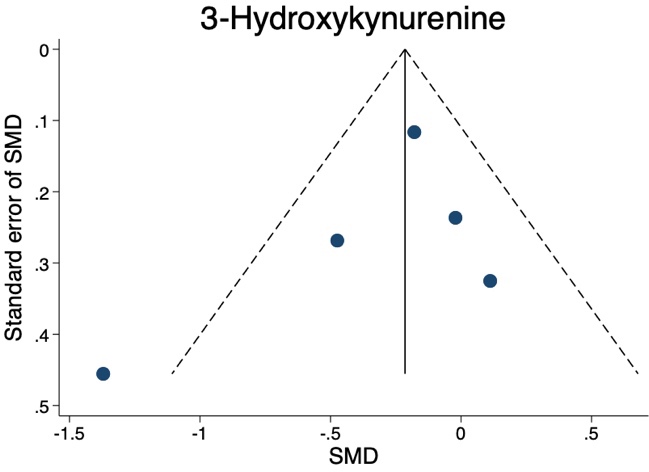 |
| E  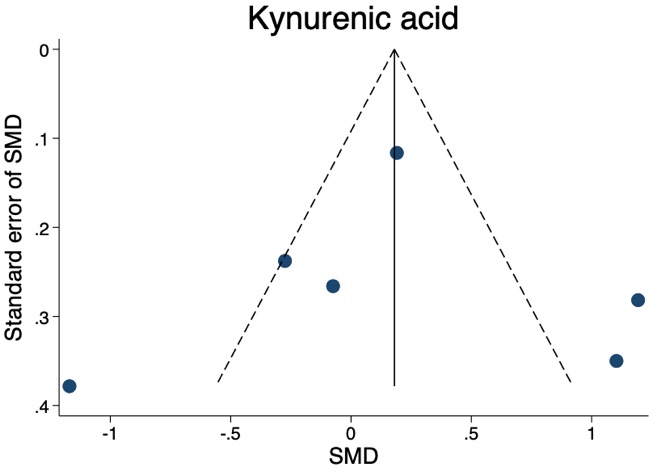 | F  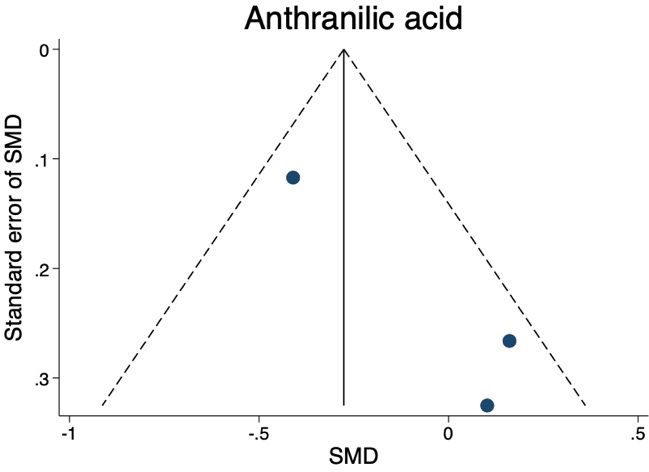 |

| G  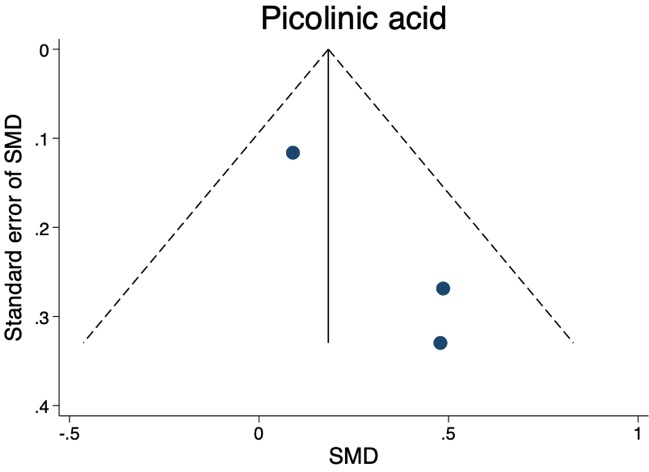 | H  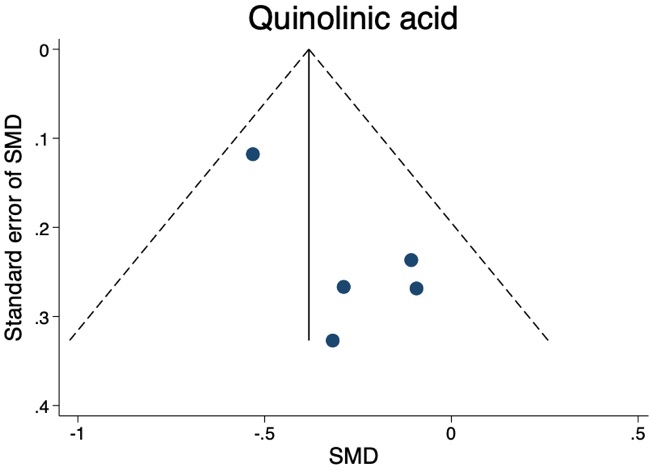 |
| --- | --- |

**Figure S2** Funnel plots of AD-control studies, separately in CSF.

| A  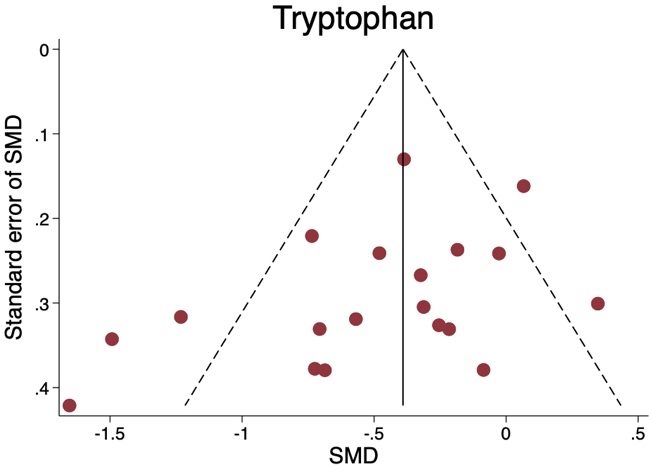 | B  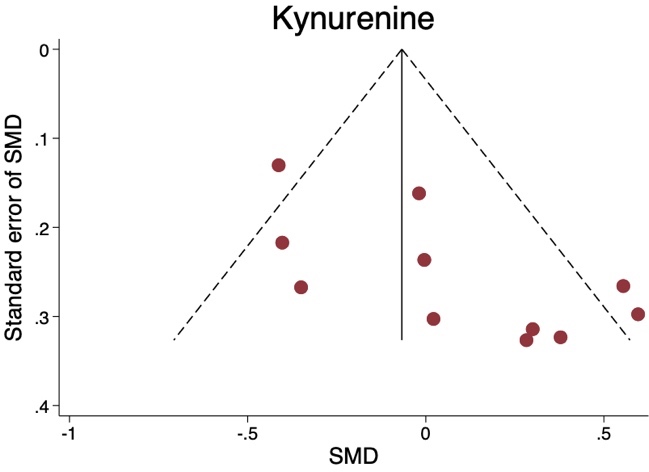 |
| --- | --- |
| C  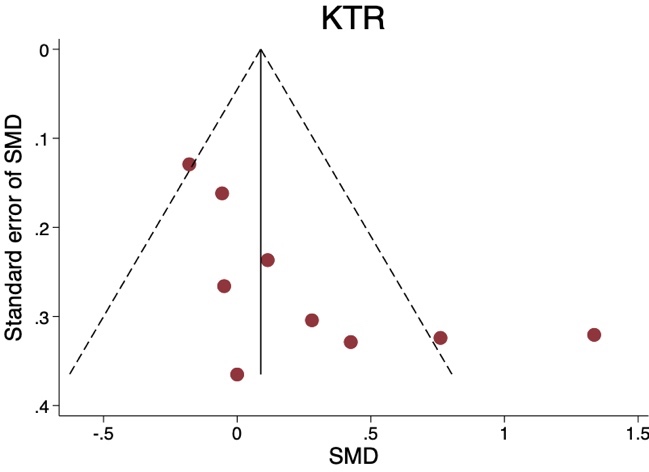 | D  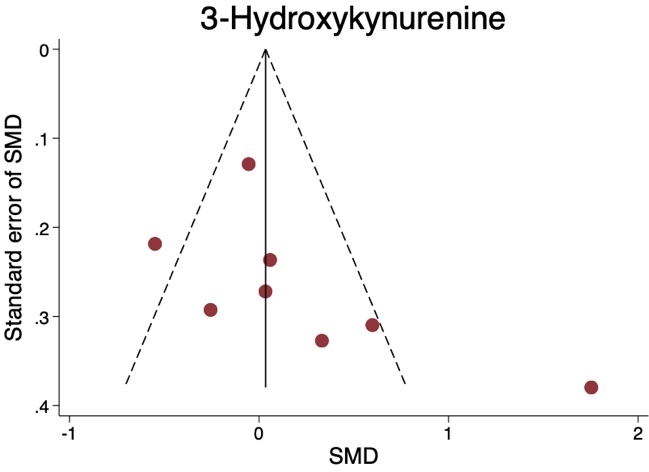 |
| E  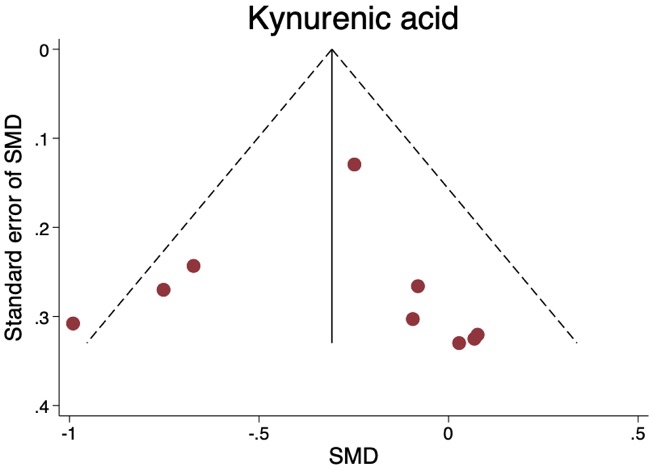 | F  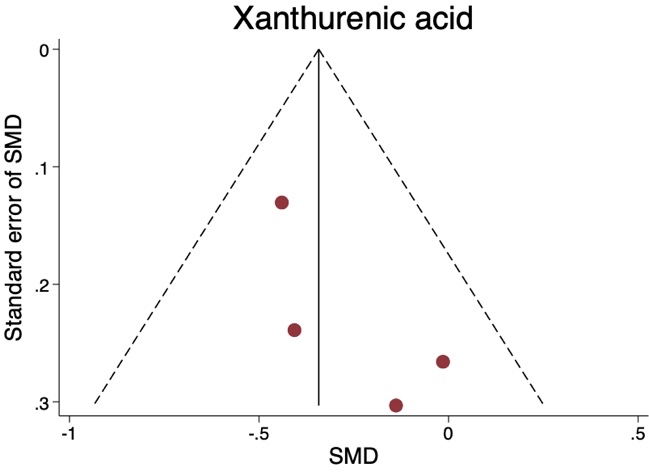 |

| G  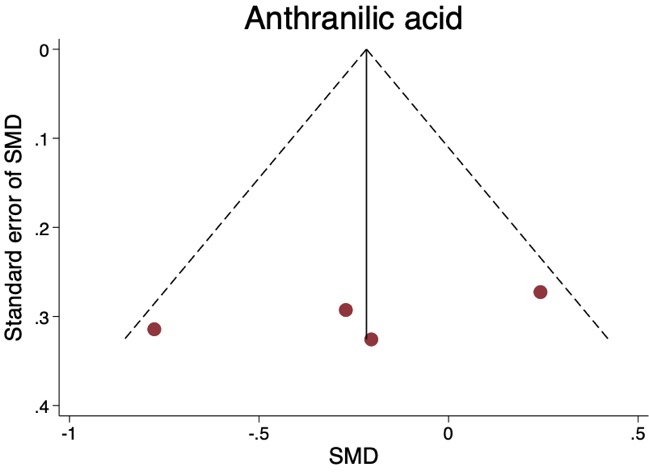 | H  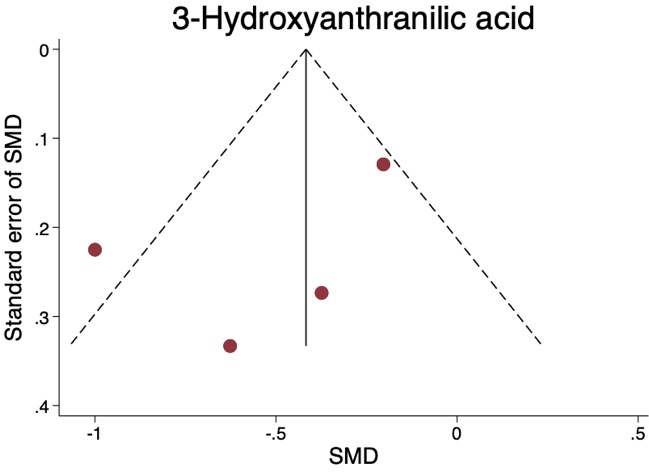 |
| --- | --- |
| I  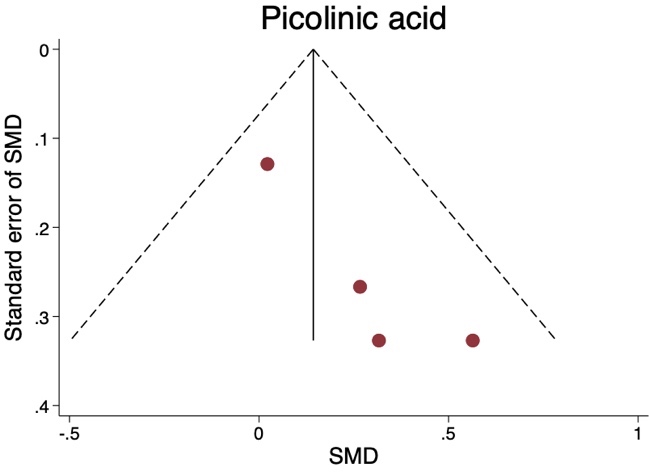 | J  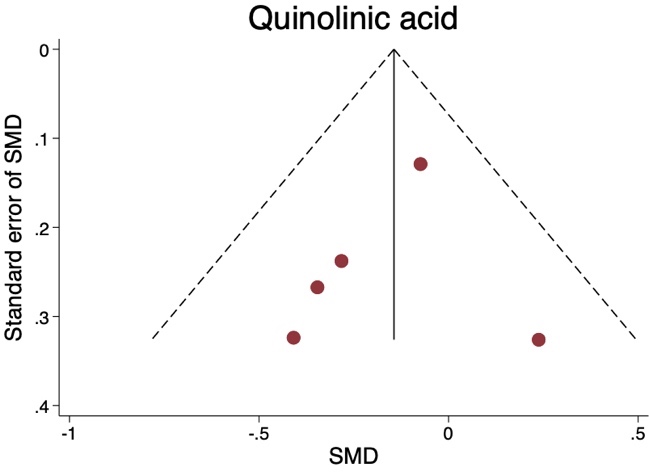 |

**Figure S3** Funnel plots of AD-control studies, separately in blood.

| A  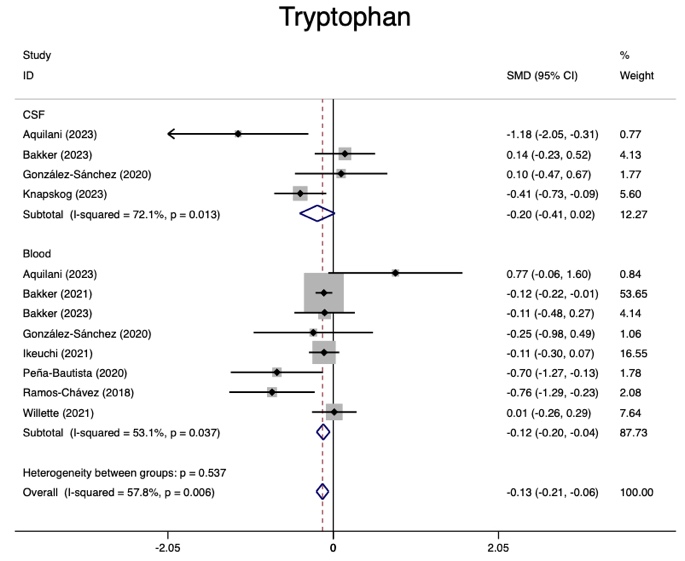 | B  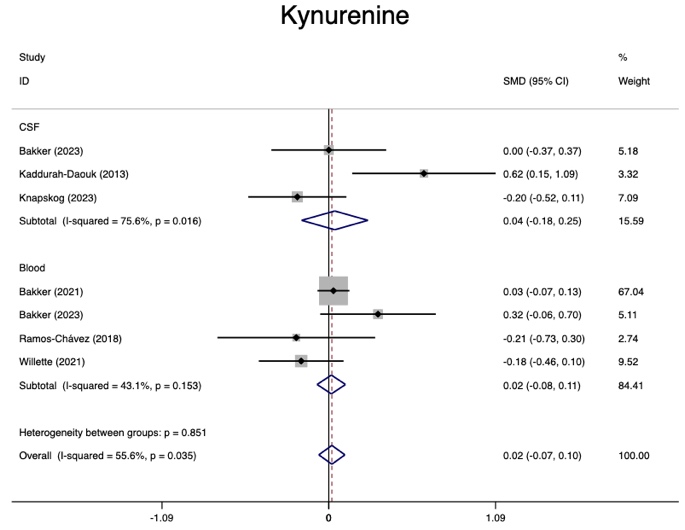 |
| --- | --- |
| C  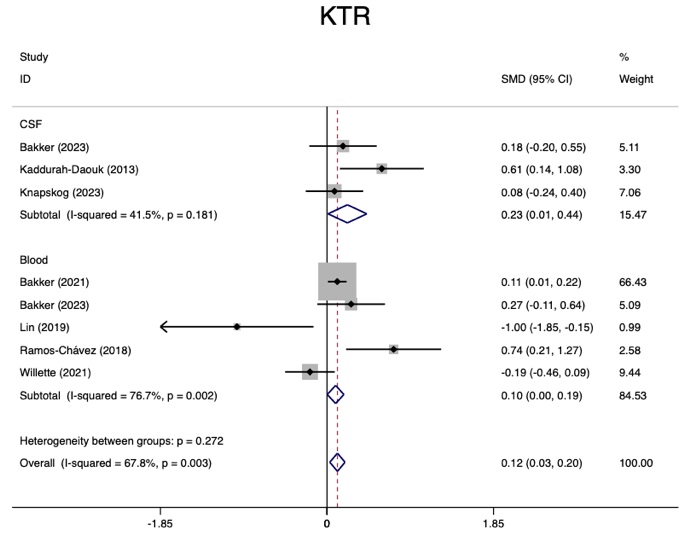 | D  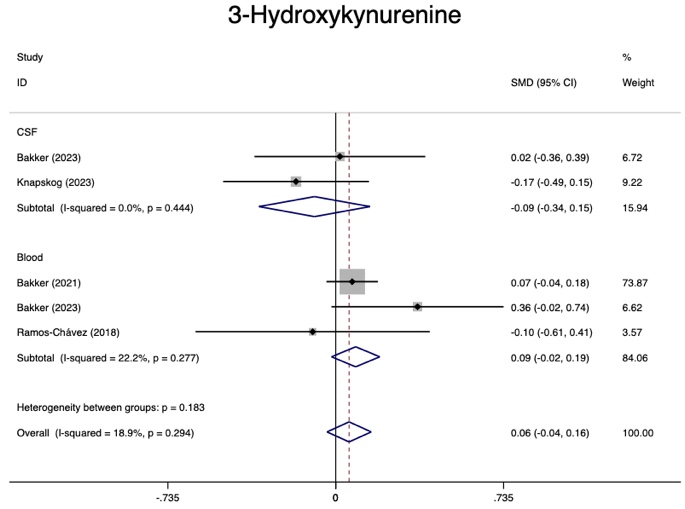 |
| E  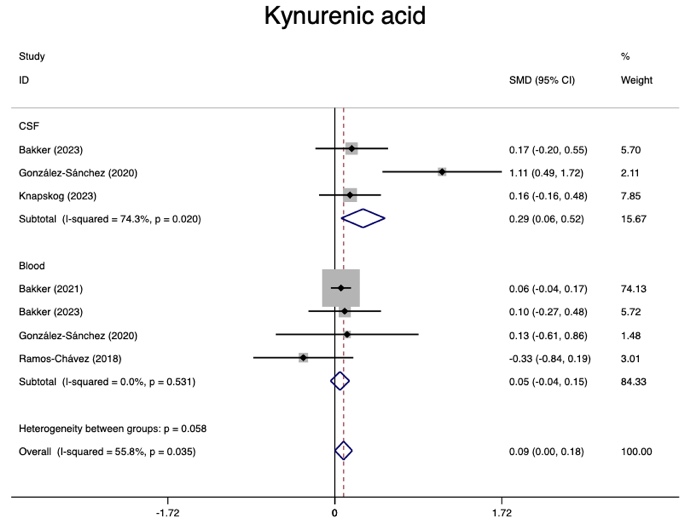 | F  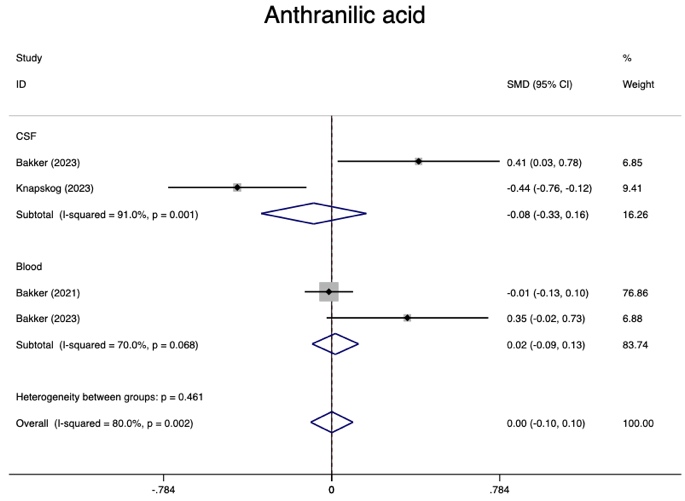 |

| G  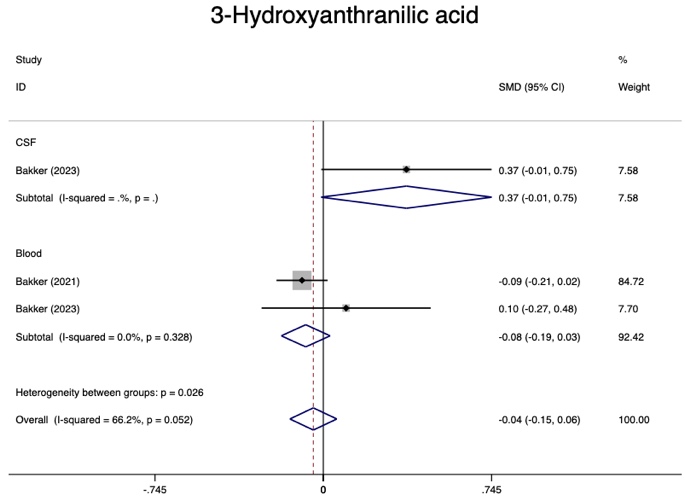 | H  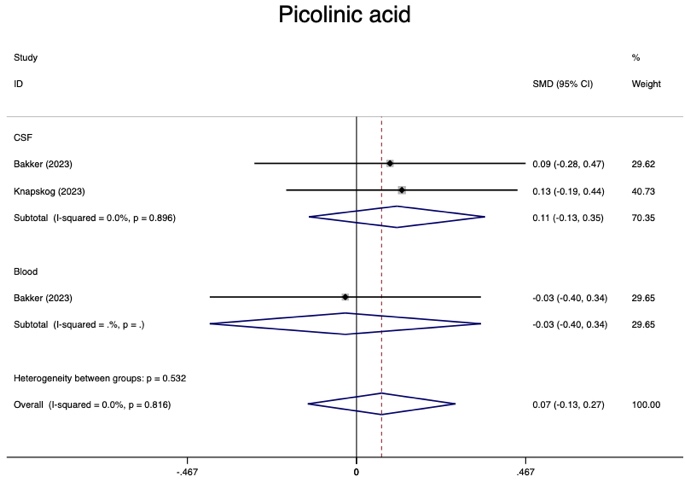 |
| --- | --- |
| I  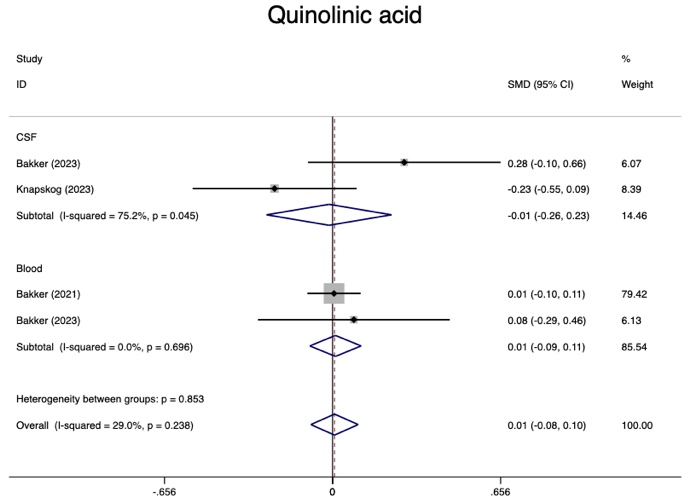 |  |

**Figure S4** Forest plots of MCI-control studies

| A  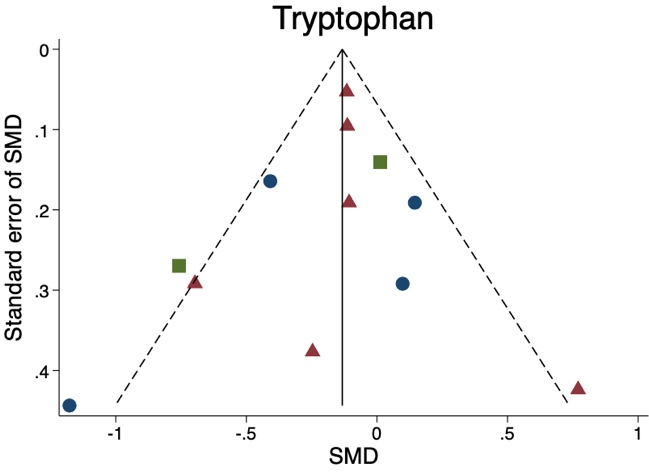 | B  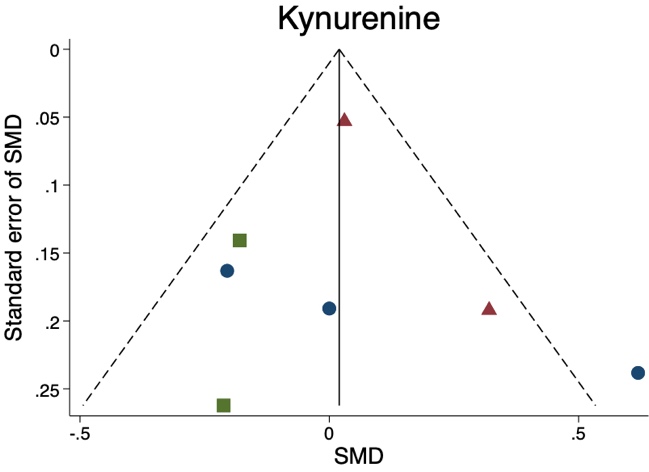 |
| --- | --- |
| C  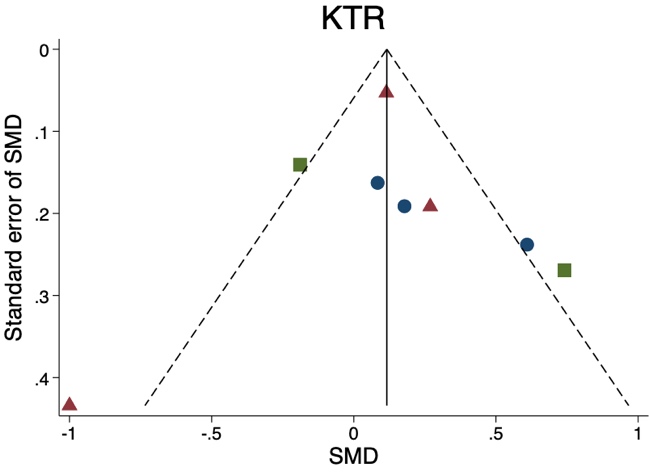 | D  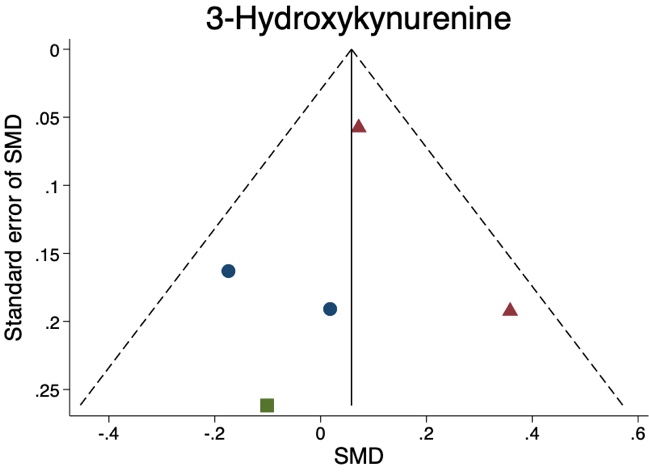 |
| E  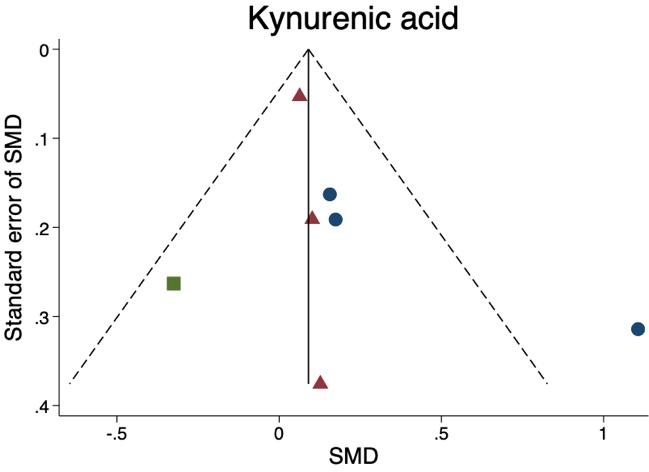 | F  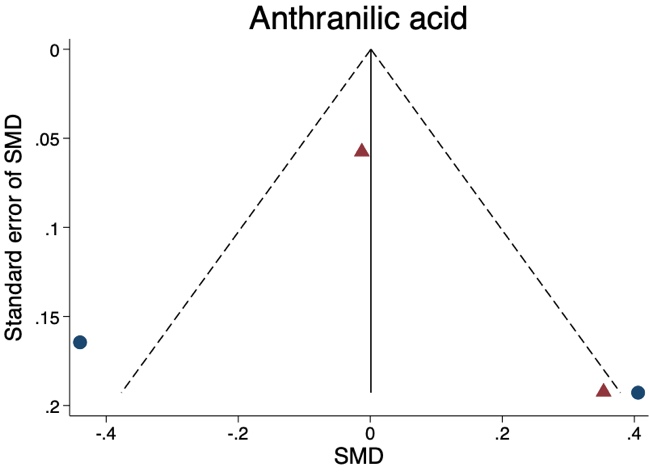 |

| G  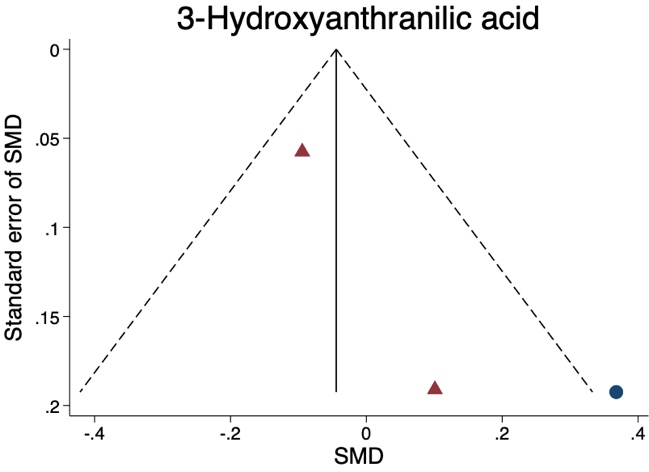 | H  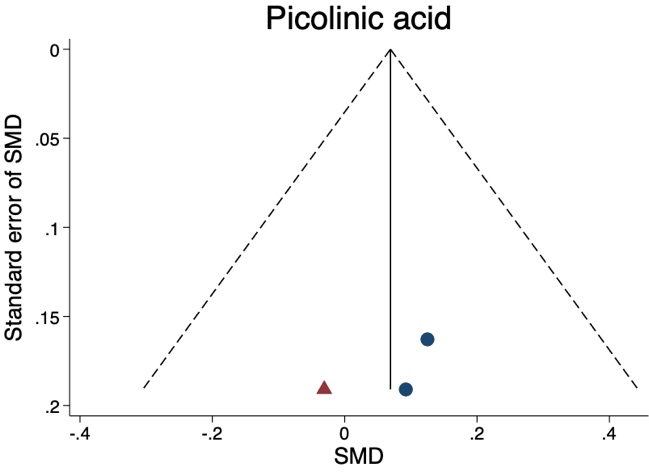 |
| --- | --- |
| I  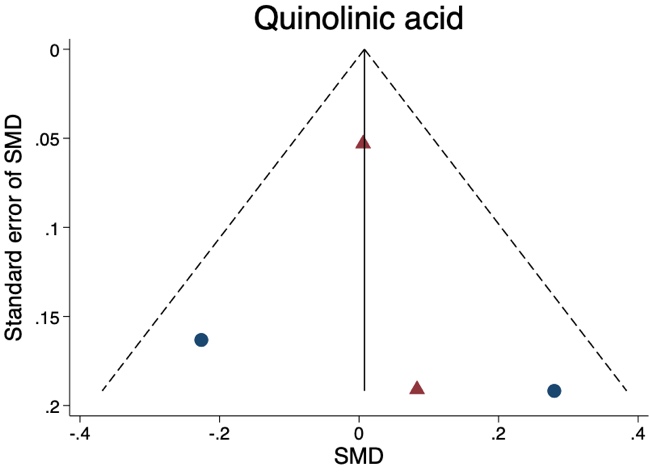 | 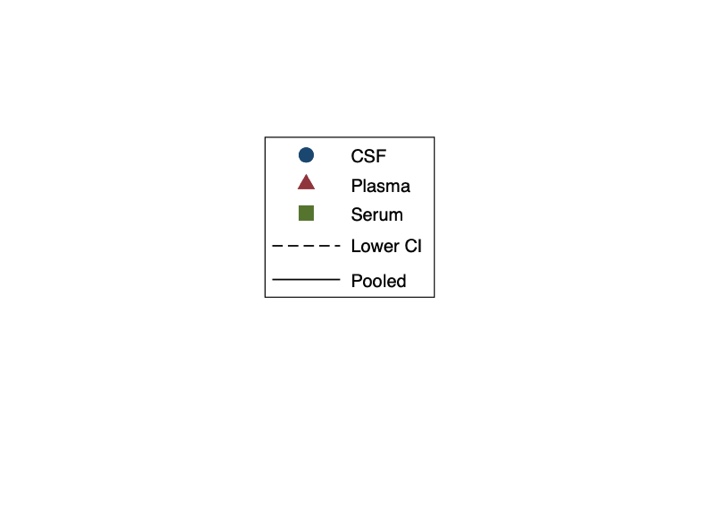 |

**Figure S5** Funnel plots of MCI-control studies.

| A  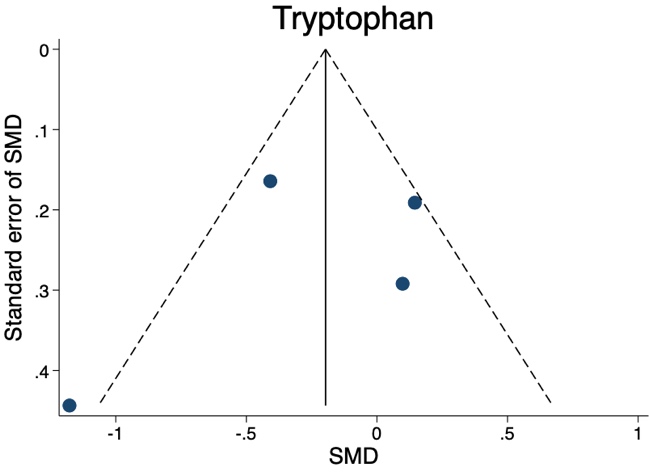 | B  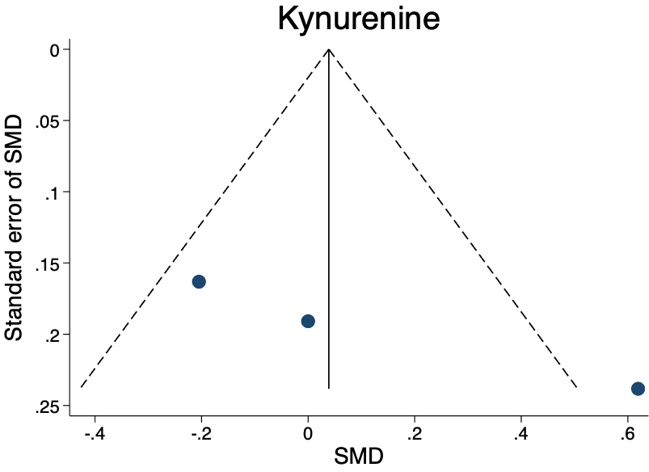 |
| --- | --- |
| C  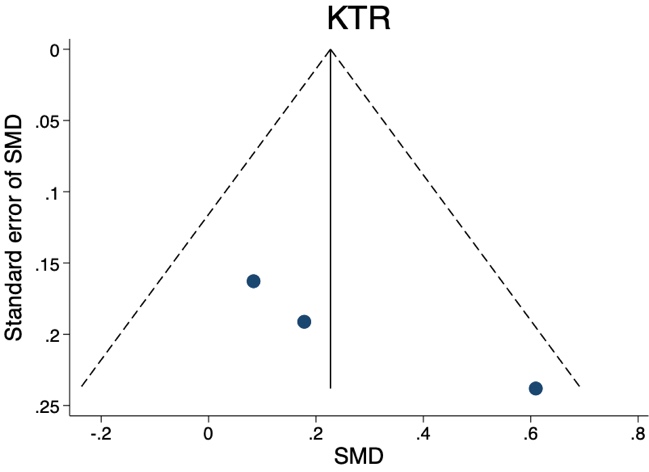 | D  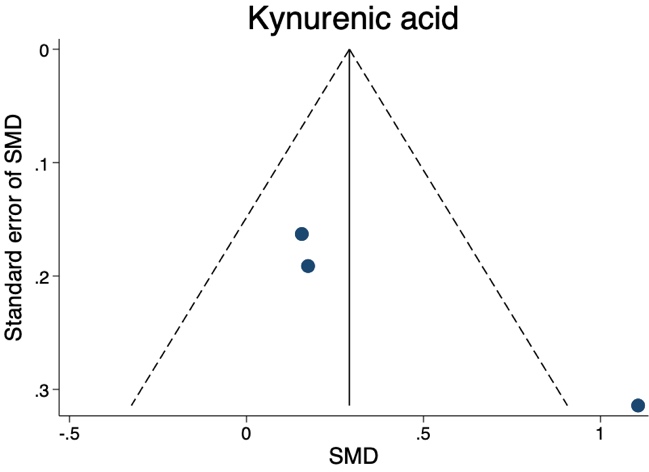 |

**Figure S6** Funnel plots of MCI-control studies, separately in CSF.

| A  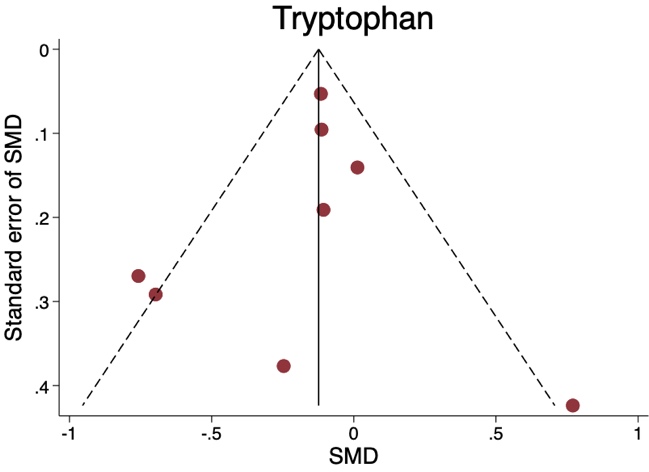 | B  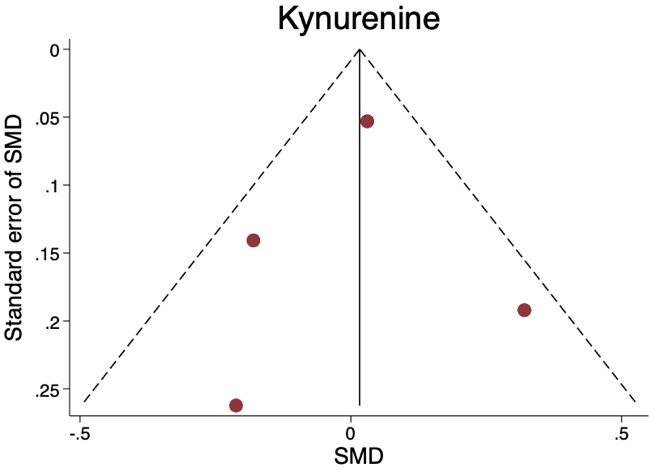 |
| --- | --- |
| C  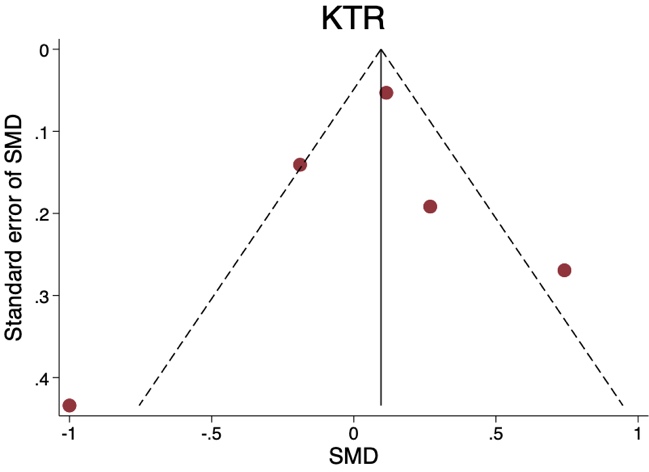 | D  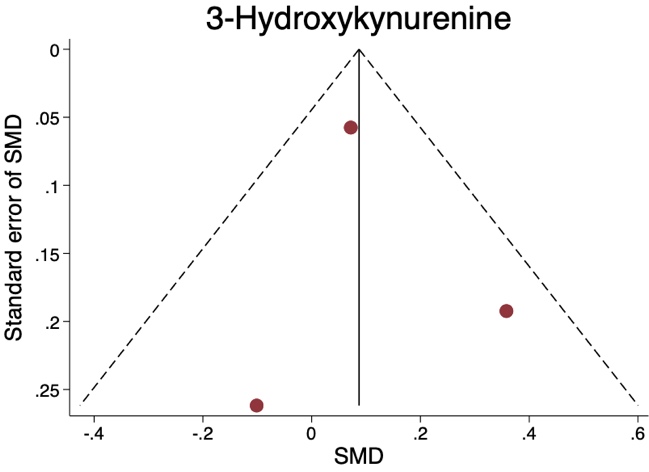 |
| E  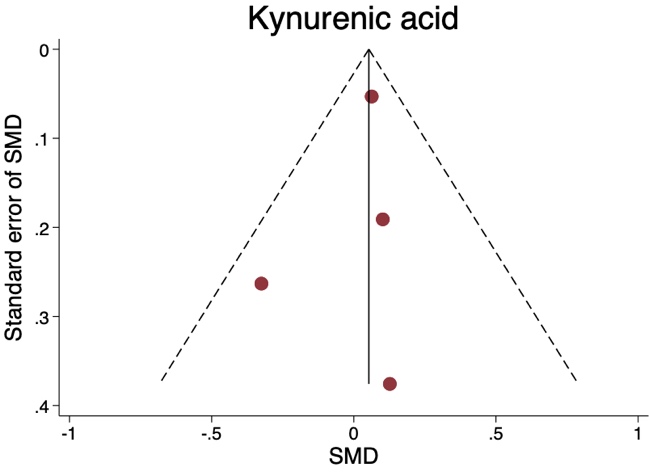 |  |

**Figure S7** Funnel plots of MCI-control studies, separately in blood.

**Appendix S1** Data extraction form.

**Study Characteristics**

| **Study ID**  *(surname of first author and year first full report of study was published e.g. Smith 2001)* |  |
| --- | --- |
| **Title** |  |

**Methods**

| **Type of study**  *(please tick)* | Case-control  Cross-sectional  Prospective  Retrospective  Other, namely ______ | | |
| --- | --- | --- | --- |
| **Participant characteristics**  *(overall)* | N:  Mean age:  Age range:  N (%) Female: | | |
| **Population description**  *(specify the groups studied (e.g. inpatient, outpatient) and/or*  *from which study participants are drawn)* |  | | |
| **Person measuring**  *(e.g. psychiatrist, researcher, study nurse. If multiple persons were involved, specify tasks per person)* |  | | |
| **Diagnosis**  *(please tick)* | Normal aging/ cognition  Cognitive impairment   - Subjective cognitive impairment (SCD) - Mild cognitive impairment (MCI) - Cognitive impairment-no dementia (CIND) - Vascular cognitive impairment (VCI) - ___ standard deviation below comparison group   Dementia   - All-cause dementia - Alzheimer’s disease dementia - Vascular dementia - Huntington’s disease - Creutzfeldt-Jakob disease - Lewy body dementia - Down syndrome - Frontotemporal dementia - Mixed dementia - Normal pressure hydrocephalus - Posterior cortical atrophy - Parkinson’s disease - Korsakoff syndrome - Unknown | | |
| **Diagnosis of dementia** *(DSM-IV, ICD-10, research criteria, unknown)* **or definition of cognitive impairment** *(e.g. Peterson criteria, 2 standard deviations below control group)* |  | | |
| **Comparator(s)/ control**  *(please tick)* | Control (no neurological disorder)  Different stages/ severity of dementia, namely ____________  Different ages of controls, namely ____________ | | |
| **Characteristics**  *(per group)* | Group:  N:  Mean age:  Range:  N (%) Female: | Group:  N:  Mean age:  Range:  N (%) Female: | Group:  N:  Mean age:  Range:  N (%) Female: |
| **Comorbidities?**  *(please tick)* | Yes, namely ____________  No  Unclear | | |
| **Setting**  *(including location and social context)* |  | | |
| **Inclusion criteria** |  | | |
| **Exclusion criteria** |  | | |
| **Method of recruitment of participants**  *(e.g. phone, mail, clinic patients)* |  | | |
| **Exclusions**  *(Number of exclusions and reason for exclusion)* |  | | |
| **Repeated assessment**  *(please tick)* | Yes, namely ____________  No  Unclear | | |
| **Neuropsychological test used**  *(include sub-domains, e.g. TMT-A, TMT-B, immediate recall CVLT, delayed recall CVLT, delayed recognition CVLT)* |  | | |

**Exposure**

| **Measured metabolites** | Tryptophan  N-formyl kynurenine  Kynurenine  3-hydroxykynurenine  Kynurenic acid  Xanthurenic acid  Anthranilic acid  3-hydroxyanthranilic acid  Cinnabarinic acid  Picolinic acid  Quinolinic acid  Other, namely ____________ |
| --- | --- |
| **Measurement technique** |  |
| **Biomaterial(s)**  *(please tick)* | Blood  Plasma  Serum  CSF  Urine  Faecal material  Saliva  Post-mortem tissues  Peripheral blood mononuclear cell  Human primary neuron  Human induced pluripotent stem cell |

| **Metabolite**  *(fill in separate form per metabolite)* |  |
| --- | --- |
| **Intra assay variations**  **Inter assay variations** |  |
| **Biomaterial(s)** |  |
| **Outcome**  *(e.g. cognitive impairment, dementia, depression)* |  |
| **Concentrations** |  |
| **Statistical methods used** *(e.g. logistic regression, mixed effects regression, Cox regression)* |  |
| **Interaction analyses?**  *(please tick)* | Yes, namely ____________  No  Unclear |
| **Adjustment for potential confounders?**  *(please tick)* | Yes, namely ____________  No  Unclear |
| **Other findings** |  |

**Other information**

| **Key conclusions of study authors** |  |
| --- | --- |
| **Correspondence for further study information** |  |
| **Notes:** | |

**Appendix S2** Newcastle-Ottawa quality assessment scale adapted for studies with different patient populations.

Note: A study can be awarded a maximum of one star for each numbered item within the Selection and Outcome categories, with the exception for ‘Assessment for outcome’ from the Outcome category. A maximum of two stars can be given for Comparability.

**Selection** (Maximum 4 stars)

**Case 1:**

1. Is the case definition adequate?

a) Yes, with independent validation *****

b) Yes, e.g. record linkage or based on self-reports

c) No description

1. Sample size, response rate, and comparability between respondent and non-respondents

a) Consecutive or obviously representative series of cases *****

b) Potential for selection biases or not stated

**Case 2:**

1. Is the case definition adequate?

a) Yes, with independent validation *****

b) Yes, e.g. record linkage or based on self-reports

c) No description

1. Sample size, response rate, and comparability between respondent and non-respondents

a) Consecutive or obviously representative series of cases *****

b) Potential for selection biases or not stated

**Comparability** (Maximum 2 stars)

Comparability of cohorts on the basis of the design or analysis

a) Study controls for the most important factor *****

b) Study controls for any additional factor *****

c) No control for any important factor

**Outcome** (Maximum 3 stars)

1. Ascertainment of exposure

a) Secure record (e.g. surgical records) *****

b) Structured interview where blind to case/control status *****

c) Interview not blinded to case/control status

d) Written self-report or medical record only

e) No description

1. Same method of ascertainment for both groups?

a) Yes *****

b) No

1. Non-Response rate

a) Same rate for both groups *****

b) Non respondents described

c) Rate different and no designation

**Appendix S3** Newcastle-Ottawa quality assessment scale adapted for cross-sectional studies.

Note: A study can be awarded a maximum of one star for each numbered item within the Selection and Outcome categories, with the exception for ‘Assessment for outcome’ from the Outcome category. A maximum of two stars can be given for Comparability.

**Selection** (Maximum 4 stars)

1. Representativeness of the exposed cohort

a) Truly representative of the general population (random sampling) *****

b) Somewhat representative of general population (non-random sampling) *****

c) Selected group of users (e.g. nurses, volunteers)

d) No description of the sampling strategy

1. Sample size, response rate, and comparability between respondent and non-respondents

a) Sample size is justified, response rate AND the comparability between respondents and non-respondents characteristics are described *****

b) Sample size is justified and the response rate OR the comparability between respondents and non-respondents characteristics is described *****

c) Sample size is justified, but no description of the response rate and the characteristics of the responders and non-responders

d) Sample size is not justified, and there is no description of the response rate or the characteristics of the responders and non-responders

1. Selection of the non-exposed cohort

a) Drawn from the same community as the exposed cohort *****

b) Drawn from a different source

c) No description of the derivation of the non-exposed cohort

1. Ascertainment of exposure

a) Validated measurement tool *****

b) Non-validated measurement tool, but the tool is available or described *****

c) Self-report

d) No description

**Comparability** (Maximum 2 stars)

Comparability of cohorts on the basis of the design or analysis

a) Study controls for the most important factor *****

b) Study controls for any additional factor *****

c) No control for any important factor

**Outcome** (Maximum 3 stars)

1. Assessment of outcome

a) Independent blind assessment ******

b) Record linkage ******

c) Self-report

d) No description

1. Statistical test

a) The statistical test used to analyse the data is clearly described and appropriate, and the measurement of the association is presented, including confidence intervals and the probability level (p value) *****

b) The statistical test is not appropriate, not described or incomplete

**References**

1. Anderson, E.W., et al., *Quinolinic acid, a kynurenine/tryptophan pathway metabolite, associates with impaired cognitive test performance in systemic lupus erythematosus.* Lupus Science and Medicine, 2021. **8**(1).

2. Aquilani, R., et al., *Several dementia subtypes and mild cognitive impairment share brain reduction of neurotransmitter precursor amino acids, impaired energy metabolism, and lipid hyperoxidation.* Frontiers in Aging Neuroscience, 2023. **15**.

3. Arai, H., et al., *A preliminary study of free amino acids in the postmortem temporal cortex from Alzheimer-type dementia patients.* Neurobiol Aging, 1984. **5**(4): p. 319-21.

4. Arai, H., et al., *Free amino acids in post-mortem cerebral cortices from patients with Alzheimer-type dementia.* Neuroscience Research, 1985. **2**(6): p. 486-490.

5. Atukeren, P., et al., *The efficacy of donepezil administration on acetylcholinesterase activity and altered redox homeostasis in Alzheimer's disease.* Biomedicine and Pharmacotherapy, 2017. **90**: p. 786-795.

6. Baker, G.B. and G.P. Reynolds, *Biogenic amines and their metabolites in Alzheimer's disease: Noradrenaline, 5-hydroxytryptamine and 5-hydroxyindole-3-acetic acid depleted in hippocampus but not in substantia innominata.* Neuroscience Letters, 1989. **100**(1-3): p. 335-339.

7. Bakker, L., et al., *Associations between plasma kynurenines and cognitive function in individuals with normal glucose metabolism, prediabetes and type 2 diabetes: the Maastricht Study.* Diabetologia, 2021. **64**(11): p. 2445-2457.

8. Bakker, L., et al., *Correlations between kynurenines in plasma and CSF, and their relation to markers of Alzheimer's disease pathology.* Brain, Behavior, and Immunity, 2023. **111**: p. 312-319.

9. Bakker, L., et al., *The role of the kynurenine pathway in cognitive functioning after stroke: A prospective clinical study.* J Neurol Sci, 2023. **454**: p. 120819.

10. Baran, H., K. Jellinger, and L. Deecke, *Kynurenine metabolism in Alzheimer's disease.* Journal of Neural Transmission, 1999. **106**(2): p. 165-181.

11. Basun, H., et al., *Amino acid concentrations in cerebrospinal fluid and plasma in Alzheimer's disease and healthy control subjects.* J Neural Transm Park Dis Dement Sect, 1990. **2**(4): p. 295-304.

12. Beal, M.F., et al., *Kynurenine pathway measurements in Huntington's disease striatum: evidence for reduced formation of kynurenic acid.* J Neurochem, 1990. **55**(4): p. 1327-39.

13. Bonaccorso, S., et al., *Serotonin-immune interactions in elderly volunteers and in patients with Alzheimer's disease (DAT): Lower plasma tryptophan availability to the brain in the elderly and increased serum interleukin-6 in DAT.* Aging - Clinical and Experimental Research, 1998. **10**(4): p. 316-323.

14. Cespedes, M., et al., *Systemic perturbations of the kynurenine pathway precede progression to dementia independently of amyloid-beta.* Neurobiol Dis, 2022. **171**: p. 105783.

15. Chatterjee, P., et al., *Plasma metabolites associated with biomarker evidence of neurodegeneration in cognitively normal older adults.* J Neurochem, 2020.

16. Chouraki, V., et al., *Association of amine biomarkers with incident dementia and Alzheimer's disease in the Framingham Study.* Alzheimer's and Dementia, 2017. **13**(12): p. 1327-1336.

17. Cogo, A., et al., *Increased serum QUIN/KYNA is a reliable biomarker of post-stroke cognitive decline.* Mol Neurodegener, 2021. **16**(1): p. 7.

18. Czech, C., et al., *Metabolite profiling of Alzheimer's disease cerebrospinal fluid.* PLoS One, 2012. **7**(2): p. e31501.

19. de Leeuw, F.A., et al., *Blood-based metabolic signatures in Alzheimer's disease.* Alzheimers Dement (Amst), 2017. **8**: p. 196-207.

20. Nik Mohd Fakhruddin, N.N.I., et al., *Urine Untargeted Metabolomic Profiling Is Associated with the Dietary Pattern of Successful Aging among Malaysian Elderly.* Nutrients, 2020. **12**(10).

21. Fekkes, D., et al., *Abnormal amino acid metabolism in patients with early stage Alzheimer dementia.* J Neural Transm (Vienna), 1998. **105**(2-3): p. 287-94.

22. Fonteh, A.N., et al., *Free amino acid and dipeptide changes in the body fluids from Alzheimer's disease subjects.* Amino Acids, 2007. **32**(2): p. 213-24.

23. Giil, L.M., et al., *Kynurenine Pathway Metabolites in Alzheimer's Disease.* Journal of Alzheimer's Disease, 2017. **60**(2): p. 495-504.

24. Gold, A.B., et al., *The relationship between indoleamine 2,3-dioxygenase activity and post-stroke cognitive impairment.* Journal of Neuroinflammation, 2011. **8 (no pagination)**.

25. Gonzalez-Dominguez, R., T. Garcia-Barrera, and J.L. Gomez-Ariza, *Metabolomic study of lipids in serum for biomarker discovery in Alzheimer's disease using direct infusion mass spectrometry.* Journal of Pharmaceutical and Biomedical Analysis, 2014. **98**: p. 321-326.

26. Gonzalez-Dominguez, R., T. Garcia-Barrera, and J.L. Gomez-Ariza, *Metabolite profiling for the identification of altered metabolic pathways in Alzheimer's disease.* Journal of Pharmaceutical and Biomedical Analysis, 2015. **107**: p. 75-81.

27. González-Domínguez, R., T. García-Barrera, and J.L. Gómez-Ariza, *Application of a novel metabolomic approach based on atmospheric pressure photoionization mass spectrometry using flow injection analysis for the study of Alzheimer׳s disease.* Talanta, 2015. **131**: p. 480-489.

28. González-Sánchez, M., et al., *Kynurenic Acid Levels are Increased in the CSF of Alzheimer's Disease Patients.* Biomolecules, 2020. **10**(4).

29. Graham, S.F., et al., *Untargeted metabolomic analysis of human plasma indicates differentially affected polyamine and L-arginine metabolism in mild cognitive impairment subjects converting to Alzheimer's disease.* PLoS One, 2015. **10**(3): p. e0119452.

30. Greilberger, J., et al., *Carbonyl proteins as a clinical marker in Alzheimer's disease and its relation to tryptophan degradation and immune activation.* Clinical Laboratory, 2010. **56**(9-10): p. 441-448.

31. Gulaj, E., et al., *Kynurenine and its metabolites in Alzheimer's disease patients.* Advances in Medical Sciences, 2010. **55**(2): p. 204-211.

32. Hafstad Solvang, S.E., et al., *Kynurenines, Neuropsychiatric Symptoms, and Cognitive Prognosis in Patients with Mild Dementia.* Int J Tryptophan Res, 2019. **12**: p. 1178646919877883.

33. Solvang, S.E.H., et al., *The kynurenine pathway and cognitive performance in community-dwelling older adults. The Hordaland Health Study.* Brain, Behavior, and Immunity, 2019. **75**: p. 155-162.

34. Hartai, Z., et al., *Decreased serum and red blood cell kynurenic acid levels in Alzheimer's disease.* Neurochemistry International, 2007. **50**(2): p. 308-313.

35. Hebbrecht, K., et al., *The Role of Kynurenines in Cognitive Dysfunction in Bipolar Disorder.* Neuropsychobiology, 2022. **81**(3): p. 184-191.

36. Heyes, M.P., et al., *Quinolinic acid and kynurenine pathway metabolism in inflammatory and non-inflammatory neurological disease.* Brain, 1992. **115 ( Pt 5)**: p. 1249-73.

37. Heylen, A., et al., *Brain Kynurenine Pathway Metabolite Levels May Reflect Extent of Neuroinflammation in ALS, FTD and Early Onset AD.* Pharmaceuticals, 2023. **16**(4).

38. Huang, J., et al., *Effects of neuroactive metabolites of the tryptophan pathway on working memory and cortical thickness in schizophrenia.* Translational Psychiatry, 2021. **11**(1).

39. Huo, Z., et al., *Brain and blood metabolome for Alzheimer's dementia: findings from a targeted metabolomics analysis.* Neurobiol Aging, 2020. **86**: p. 123-133.

40. Ibáñez, C., et al., *A new metabolomic workflow for early detection of Alzheimer's disease.* J Chromatogr A, 2013. **1302**: p. 65-71.

41. Ikeuchi, T., et al., *Development of a Novel Nutrition-Related Multivariate Biomarker for Mild Cognitive Impairment Based on the Plasma Free Amino Acid Profile.* Nutrients, 2022. **14**(3).

42. Jacobs, K.R., et al., *Correlation between plasma and CSF concentrations of kynurenine pathway metabolites in Alzheimer's disease and relationship to amyloid-β and tau.* Neurobiology of Aging, 2019. **80**: p. 11-20.

43. Janssens, J., et al., *Monoaminergic and Kynurenergic Characterization of Frontotemporal Dementia and Amyotrophic Lateral Sclerosis in Cerebrospinal Fluid and Serum.* Neurochem Res, 2020. **45**(5): p. 1191-1201.

44. Kaddurah-Daouk, R., et al., *Metabolomic changes in autopsy-confirmed Alzheimer's disease.* Alzheimer's and Dementia, 2011. **7**(3): p. 309-317.

45. Kaddurah-Daouk, R., et al., *Alterations in metabolic pathways and networks in Alzheimer's disease.* Translational Psychiatry, 2013. **3 (no pagination)**.

46. Kaiser, E., et al., *Cerebrospinal fluid concentrations of functionally important amino acids and metabolic compounds in patients with mild cognitive impairment and Alzheimer's disease.* Neurodegenerative Diseases, 2010. **7**(4): p. 251-259.

47. Kindler, J., et al., *Dysregulation of kynurenine metabolism is related to proinflammatory cytokines, attention, and prefrontal cortex volume in schizophrenia.* Mol Psychiatry, 2020. **25**(11): p. 2860-2872.

48. Klatt, S., et al., *A six-metabolite panel as potential blood-based biomarkers for Parkinson’s disease.* npj Parkinson's Disease, 2021. **7**(1).

49. Knapskog, A.B., et al., *Higher concentrations of kynurenic acid in CSF are associated with the slower clinical progression of Alzheimer's disease.* Alzheimer's and Dementia, 2023.

50. Kuster, O.C., et al., *Novel Blood-Based Biomarkers of Cognition, Stress, and Physical or Cognitive Training in Older Adults at Risk of Dementia: Preliminary Evidence for a Role of BDNF, Irisin, and the Kynurenine Pathway.* Journal of Alzheimer's Disease, 2017. **59**(3): p. 1097-1111.

51. Leblhuber, F., et al., *Activated immune system in patients with Huntington's disease.* Clinical Chemistry and Laboratory Medicine, 1998. **36**(10): p. 747-750.

52. Li, N.J., et al., *Plasma metabolic profiling of Alzheimer's disease by liquid chromatography/mass spectrometry.* Clinical Biochemistry, 2010. **43**(12): p. 992-997.

53. Liang, Q., et al., *High-throughput metabolomics analysis discovers salivary biomarkers for predicting mild cognitive impairment and Alzheimer's disease.* RSC Advances, 2016. **6**(79): p. 75499-75504.

54. Lin, C.N., et al., *A metabolomic approach to identifying biomarkers in blood of Alzheimer's disease.* Ann Clin Transl Neurol, 2019. **6**(3): p. 537-545.

55. Liu, M., et al., *Potential of serum metabolites for diagnosing post-stroke cognitive impairment.* Molecular bioSystems, 2015. **11**(12): p. 3287-3296.

56. Liu, M., et al., *Chiral Amino Acid Profiling in Serum Reveals Potential Biomarkers for Alzheimer's Disease.* Journal of Alzheimer's Disease, 2023. **94**(1): p. 291-301.

57. Martinez, M., et al., *Amino acid concentrations in cerebrospinal fluid and serum in Alzheimer's disease and vascular dementia.* J Neural Transm Park Dis Dement Sect, 1993. **6**(1): p. 1-9.

58. Mashige, F., et al., *Development of a high-performance liquid chromatography system with multi- electrode electrochemical detectors for determination of levels of neurotransmitters in cerebrospinal fluid.* Japanese Journal of Clinical Chemistry, 1993. **22**(3): p. 147-155.

59. McCann, A., et al., *Serum tyrosine is associated with better cognition in Lewy body dementia.* Brain Research, 2021. **1765**.

60. Molina, J.A., et al., *Cerebrospinal fluid levels of non-neurotransmitter amino acids in patients with Alzheimer's disease.* J Neural Transm (Vienna), 1998. **105**(2-3): p. 279-86.

61. Mourdian, M.M., et al., *No changes in central quinolinic acid levels in Alzheimer's disease.* Neurosci Lett, 1989. **105**(1-2): p. 233-8.

62. Nho, K., et al., *Serum metabolites associated with brain amyloid beta deposition, cognition and dementia progression.* Brain Commun, 2021. **3**(3): p. fcab139.

63. Oxenkrug, G., et al., *Peripheral Tryptophan - Kynurenine Metabolism Associated with Metabolic Syndrome is Different in Parkinson's and Alzheimer's Diseases.* Endocrinol Diabetes Metab J, 2017. **1**(4).

64. Paglia, G., et al., *Unbiased Metabolomic Investigation of Alzheimer’s Disease Brain Points to Dysregulation of Mitochondrial Aspartate Metabolism.* Journal of Proteome Research, 2016. **15**(2): p. 608-618.

65. Park, S.A., et al., *Metabolite Profiling Revealed That a Gardening Activity Program Improves Cognitive Ability Correlated with BDNF Levels and Serotonin Metabolism in the Elderly.* Int J Environ Res Public Health, 2020. **17**(2).

66. Parker, D.C., et al., *Tryptophan Metabolism and Neurodegeneration: Longitudinal Associations of Kynurenine Pathway Metabolites with Cognitive Performance and Plasma Alzheimer's Disease and Related Dementias Biomarkers in the Duke Physical Performance Across the LifeSpan Study.* Journal of Alzheimer's Disease, 2023. **91**(3): p. 1141-1150.

67. Peña-Bautista, C., et al., *Plasma alterations in cholinergic and serotonergic systems in early Alzheimer Disease: Diagnosis utility.* Clin Chim Acta, 2020. **500**: p. 233-240.

68. Platzer, M., et al., *Tryptophan breakdown and cognition in bipolar disorder.* Psychoneuroendocrinology, 2017. **81**: p. 144-150.

69. Ramos-Chavez, L.A., et al., *Low serum tryptophan levels as an indicator of global cognitive performance in nondemented women over 50 years of age.* Oxidative Medicine and Cellular Longevity, 2018. **2018 (no pagination)**.

70. Rommer, P.S., et al., *Lowered levels of carbonyl proteins after Vitamin B supplementation in patients with mild cognitive impairment and Alzheimer's disease.* Neurodegenerative Diseases, 2016. **16**(3-4): p. 284-289.

71. Rudman, D., et al., *Fasting plasma amino acids in elderly men.* Am J Clin Nutr, 1989. **49**(3): p. 559-66.

72. Santos, A.L.M., et al., *Frontotemporal dementia: Plasma metabolomic signature using gas chromatography-mass spectrometry.* J Pharm Biomed Anal, 2020. **189**: p. 113424.

73. Schwarz, M.J., et al., *Increased 3-Hydroxykynurenine serum concentrations differentiate Alzheimer's disease patients from controls.* European Archives of Psychiatry and Clinical Neuroscience, 2013. **263**(4): p. 345-352.

74. Shao, Y., et al., *Alteration of Metabolic Profile and Potential Biomarkers in the Plasma of Alzheimer's Disease.* Aging Dis, 2020. **11**(6): p. 1459-1470.

75. Shaw, D.M., et al., *Pilot study of amino acids in senile dementia.* Br J Psychiatry, 1981. **139**: p. 580-2.

76. Sorgdrager, F.J.H., et al., *Age- and disease-specific changes of the kynurenine pathway in Parkinson's and Alzheimer's disease.* Journal of Neurochemistry., 2019.

77. Storga, D., et al., *Monoaminergic neurotransmitters, their precursors and metabolites in brains of Alzheimer patients.* Neurosci Lett, 1996. **203**(1): p. 29-32.

78. Tarbit, I., et al., *Hippocampal free amino acids in Alzheimer's disease.* J Neurochem, 1980. **35**(5): p. 1246-9.

79. Teruya, T., et al., *Whole-blood metabolomics of dementia patients reveal classes of disease-linked metabolites.* Proceedings of the National Academy of Sciences of the United States of America, 2021. **118**(37).

80. Thomas, D.E., et al., *Tryptophan and nutritional status of patients with senile dementia.* Psychol Med, 1986. **16**(2): p. 297-305.

81. Tohgi, H., et al., *Concentrations of serotonin and its related substances in the cerebrospinal fluid in patients with Alzheimer type dementia.* Neuroscience Letters, 1992. **141**(1): p. 9-12.

82. Tohgi, H., et al., *Indoleamine concentrations in cerebrospinal fluid from patients with Alzheimer type and Binswanger type dementias before and after administration of citalopram, a synthetic serotonin uptake inhibitor.* Journal of Neural Transmission - Parkinson's Disease and Dementia Section, 1995. **9**(2-3): p. 121-131.

83. Toledo, J.B., et al., *Metabolic network failures in Alzheimer's disease: A biochemical road map.* Alzheimer's and Dementia, 2017. **13**(9): p. 965-984.

84. Trushina, E., et al., *Identification of altered metabolic pathways in plasma and CSF in mild cognitive impairment and Alzheimer's disease using metabolomics.* PLoS One, 2013. **8**(5): p. e63644.

85. Tsuruoka, M., et al., *Capillary electrophoresis-mass spectrometry-based metabolome analysis of serum and saliva from neurodegenerative dementia patients.* Electrophoresis, 2013. **34**(19): p. 2865-72.

86. van der Velpen, V., et al., *Systemic and central nervous system metabolic alterations in Alzheimer's disease.* Alzheimers Res Ther, 2019. **11**(1): p. 93.

87. Vints, W.A.J., et al., *Inflammatory Blood Biomarker Kynurenine Is Linked With Elevated Neuroinflammation and Neurodegeneration in Older Adults: Evidence From Two 1H-MRS Post-Processing Analysis Methods.* Frontiers in Psychiatry, 2022. **13**.

88. Watkins, S.E., et al., *Plasma amino acids in patients with senile dementia and in subjects with Down's syndrome at an age vulnerable to Alzheimer changes.* Journal of Mental Deficiency Research, 1989. **33**(2): p. 159-166.

89. Wennstrom, M., et al., *Kynurenic Acid levels in cerebrospinal fluid from patients with Alzheimer's disease or dementia with lewy bodies.* Int J Tryptophan Res, 2014. **7**: p. 1-7.

90. Whiley, L., et al., *Metabolic phenotyping reveals a reduction in the bioavailability of serotonin and kynurenine pathway metabolites in both the urine and serum of individuals living with Alzheimer's disease.* Alzheimers Res Ther, 2021. **13**(1): p. 20.

91. Widner, B., et al., *Degradation of tryptophan in neurodegenerative disorders.* Adv Exp Med Biol, 1999. **467**: p. 133-8.

92. Widner, B., et al., *Tryptophan degradation and immune activation in Alzheimer's disease.* Journal of Neural Transmission, 2000. **107**(3): p. 343-353.

93. Willette, A.A., et al., *Inflammation, Negative Affect, and Amyloid Burden in Alzheimer's Disease: Insights from the Kynurenine Pathway.* Brain Behav Immun, 2021.

94. Wissmann, P., et al., *Immune activation in patients with Alzheimer's disease is associated with high serum phenylalanine concentrations.* Journal of the Neurological Sciences, 2013. **329**(1-2): p. 29-33.

95. Wu, L., et al., *Altered Gut Microbial Metabolites in Amnestic Mild Cognitive Impairment and Alzheimer's Disease: Signals in Host-Microbe Interplay.* Nutrients, 2021. **13**(1).

96. Xu, J., et al., *Graded perturbations of metabolism in multiple regions of human brain in Alzheimer's disease: Snapshot of a pervasive metabolic disorder.* Biochim Biophys Acta, 2016. **1862**(6): p. 1084-92.

97. Xu, J., et al., *Sex-Specific Metabolic Pathways Were Associated with Alzheimer's Disease (AD) Endophenotypes in the European Medical Information Framework for AD Multimodal Biomarker Discovery Cohort.* Biomedicines, 2021. **9**(11).

98. Yilmaz, A., et al., *Targeted Metabolic Profiling of Urine Highlights a Potential Biomarker Panel for the Diagnosis of Alzheimer's Disease and Mild Cognitive Impairment: A Pilot Study.* Metabolites, 2020. **10**(9).
